# Photoinduced Processes in Rhenium(I) Terpyridine Complexes Bearing Remote Amine Groups: New Insights from Transient Absorption Spectroscopy

**DOI:** 10.3390/molecules27217147

**Published:** 2022-10-22

**Authors:** Joanna Palion-Gazda, Agata Szłapa-Kula, Mateusz Penkala, Karol Erfurt, Barbara Machura

**Affiliations:** 1 Institute of Chemistry, University of Silesia, Szkolna 9, 40-006 Katowice, Poland; 2 Department of Chemical Organic Technology and Petrochemistry, Silesian University of Technology, Krzywoustego 4, 44-100 Gliwice, Poland

**Keywords:** Re(I) chromophore, 2,2′:6′,2″-terpyridine derivatives, intraligand and metal-to-ligand charge-transfer processes, solvent polarity-induced photoluminescence lifetimes

## Abstract

Photophysical properties of two Re(I) complexes [ReCl(CO)_3_(R-C_6_H_4_-terpy-κ^2^N)] with remote amine groups, *N*-methyl-piperazinyl (**1**) and (2-cyanoethyl)methylamine (**2**), were investigated. The complexes show strong absorption in the visible region corresponding to metal-to-ligand charge transfer (^1^MLCT) and intraligand-charge-transfer (^1^ILCT) transitions. The energy levels of ^3^MLCT and ^3^ILCT excited-states, and thus photoluminescence properties of **1** and **2**, were found to be strongly affected by the solvent polarity. Compared to the parent chromophore [ReCl(CO)_3_(C_6_H_5_-terpy-κ^2^N)] (**3**), both designed complexes show significantly prolonged (by 1–2 orders of magnitude) phosphorescence lifetimes in acetonitrile and dimethylformamide, contrary to their lifetimes in less polar chloroform and tetrahydrofuran, which are comparable to those for **3**. The femtosecond transient absorption (fsTA) measurements confirmed the interconversion between the ^3^MLCT and ^3^ILCT excited-states in polar solvents. In contrast, the emissive state of **1** and **2** in less polar environments is of predominant ^3^MLCT nature.

## 1. Introduction

The first report on the photoluminescence of Re(I) carbonyl complexes *fac*-[ReX(CO)_3_(N^∩^N)] with 1,10-phenanthroline and its derivatives in solution at room temperature, ascribed to the ^3^MLCT [d(Re)–π_phen_] excited-state origin, was published in 1974 [1]. Since this groundbreaking work of Wrighton and Morse, neutral and monocationic complexes of type *fac*-[ReX(CO)_3_(N^∩^N)]^n^ (N^∩^N = diamine, X = halides, pseudohalides, pyridine, imidazole, and related N-heterocycles, n = 0 or 1+) have become a topic of intense research due to their intriguing photoluminescent properties [2,3,4,5,6,7,8,9,10,11,12,13,14,15,16,17,18,19] and potential applications for photocatalytic carbon dioxide reduction [20,21,22,23], organic light-emitting diodes [24,25,26,27,28,29], solar energy conversion [30,31,32,33], and medicinal applications [34,35,36,37,38,39,40,41,42,43,44]. Taking advantage of the relatively simple synthetic procedures of *fac*-[ReX(CO)_3_(N^∩^N)]^n^ and wide range of possible structural modification of α-diimine ligands, it has been possible to perform systematic studies of these systems. Consequently, Re(I) carbonyls have highly contributed to an understanding of photophysical and light-induced electron-transfer and electronic energy-transfer processes in transition metal complexes. It has been shown that introduction of suitable substituents into α-diimine core and alteration of the ancillary ligands (X) not only fine-tunes the energy and intensity of the emission from the triplet ^3^MLCT excited state, but also can make changes in the ordering and energetic spacing between the excited states. In the latter case, photophysical properties of resultant complexes *fac*-[ReX(CO)_3_(N^∩^N)]^n^ are determined by excited states other than MLCT, namely LLCT (ligand-to-ligand-charge-transfer), IL (intraligand), and ILCT (intraligand-charge-transfer), or their superposition [2,3,5,6,45,46,47].

In recent years, particular attention has been paid to the development of Re(I) with a small energy gap between two close-lying energy triplet excited states ^3^MLCT and ^3^IL, allowing formation of the excited-state equilibrium between these levels. A typical feature of such systems is an extraordinarily long-emission lifetime, which greatly exceeds the value observed for the parent ^3^MLCT chromophore [1,48,49,50,51,52,53]. One of the methods for introducing the triplet states into equilibrium is to append organic chromophore with the appropriate triplet energetics to interact with the charge-transfer state [54]. The best example is Re(I) carbonyl complex bearing N-(1,10-phenanthroline)-4-(1-piperidinyl)naphthalene-1,8-dicarboximide, for which the photoluminescence lifetime was increased approximately 3000-fold at room temperature relative to that of the parent complex [ReCl(CO)_3_(phen)]. Its excited-state lifetime (τ = 651 μs) is one of the longest, observed for excited-state lifetime for Re(I)-based CT chromophores at room temperature [50].

Prolonged lifetimes were also confirmed for some Re(I) carbonyls with emitting state of ^3^ILCT nature, for example [ReX(CO)_3_(phen-TPA)] (X = Cl, Br) and [ReCl(CO)_3_(Me_2_N-C_6_H_4_-terpy-κ^2^N)] [53,55]. This approach opens possibilities to modulate the properties of these systems, especially that the introduction of donor-π-acceptor type (D-A, D-π-A) ligands into a heavy metal coordination sphere is an effective way for lowering the energy gap of compounds to realize deep red or NIR light emission and offer additional advantages in optoelectronic properties due to efficient hole-transporting abilities of the bipolar systems [13,56,57]. Regarding the convenience of structural modifications of the 2,2′:6′,2″-terpyridine framework [58,59,60,61,62,63,64,65], [ReCl(CO)_3_(R-C_6_H_4_-terpy-κ^2^N)] complexes of bidentate-coordinated 4′-(4-substituted-phenyl)-terpyridine-like ligands with strong electron-donating groups were found to be suitable systems for studies of photoinduced ILCT processes, and they were regarded as promising photoluminescence materials [55,66]. 

This study is focused on the examination of the effect of the aliphatic cyclic amine (*N*-methyl-piperazinyl) and aliphatic amine with an additional electron-withdrawing C≡N unit (-N(CH_3_)(CH_2_CH_2_CN)) attached to the central pyridine ring of the terpy core via phenylene linkage on ground- and excited-state properties of resulting Re(I) complexes. 

**Scheme 1:**
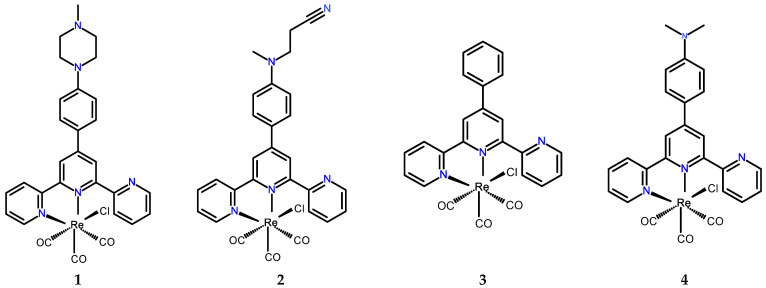
Structural formulas of Re(I) complexes.

The impact of the remote amine groups on photophysical properties of [ReCl(CO)_3_(N-methyl-piperazinyl-C_6_H_4_-terpy-κ^2^N)] (**1**) and [ReCl(CO)_3_((CH_3_)(CH_2_CH_2_CN)N-C_6_H_4_-terpy-κ^2^N)] (**2**) were examined with the use of absorption and emission spectroscopies, transient absorption. It was also stimulated by the density functional theory (DFT) and time-dependent DFT methods. To obtain a deeper understanding of structure-property relationships, the ground- and excited-state properties of **1** and **2** were evaluated in relation to the previously reported [ReCl(CO)_3_(C_6_H_5_-terpy-κ^2^N)] (**3**) and [ReCl(CO)_3_(Me_2_N-C_6_H_4_-terpy-κ^2^N)] (**4**) (Scheme 1). While the parent chromophore (**3**) is a typical ^3^MLCT emitter [67], time-resolved infrared (TR-IR) spectroscopy studies of Re(I) complex with *N*,*N*-dimethylamine remote group (**4**) confirmed the ILCT nature of its lowest triplet state [55]. 

Here, we show that the interplay between ^3^MLCT and ^3^IL/^3^ILCT excited states in complexes **1** and **2** is also strongly affected by solvent polarity. In polar solvents, the incorporation of *N*-methyl-piperazinyl and (2-cyanoethyl)methylamine leads to a significant enhancement of the excited-state lifetime relative to complex **3** due to the interconversion of ^3^MLCT into ^3^ILCT at early time delays. In solvents with a low polarity, a crucial role in determining photophysical properties of **1** and **2** plays ^3^MLCT excited state. 

## 2. Results

### 2.1. Synthesis and Molecular Structures

The complexes **1** and **2** (Scheme 1) were prepared by reacting the respective 4′-(4-substituted-phenyl)-terpyridine ligand with the rhenium(I) complex [Re(CO)_5_Cl] in refluxing acetonitrile under argon atmosphere. The molecular structures of **1** and **2** were established with the aid of ^1^H and ^13^C NMR, and HRMS spectra, elemental analysis, and single-crystal X-ray analysis for **1**. 

**Figure 1:**
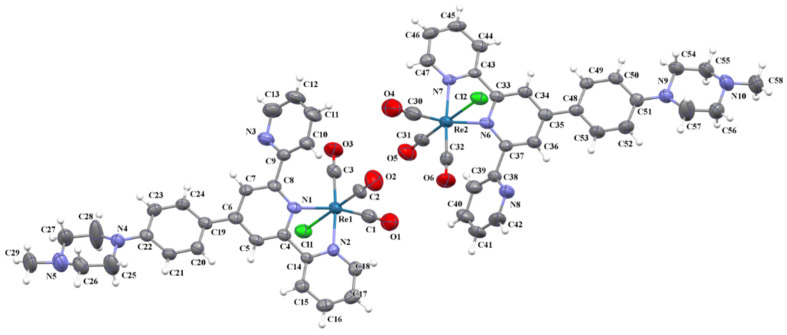
The asymmetric unit of **1** with the atom numbering and thermal ellipsoids set at 50% probability for non-hydrogen atoms. Selected bond lengths (Å) and angles (°) associated with the Re(1) ion: Re(1)–N(1) 2.205(4), Re(1)–N(2) 2.171(4), Re(1)–C(1) 1.893(6), Re(1)–C(2) 1.926(6), Re(1)–C(3) 1.927(6), Re(1)-Cl(1) 2.4889(13), N(1)–Re(1)–N(2) 74.26(14), N(1)–Re(1)–C(1) 168.86(16), N(1)–Re(1)–C(2) 92.7(2), N(1)–Re(1)–C(3) 102.38(19), N(2)–Re(1)–C(1) 96.94(19), N(2)–Re(1)–C(2) 92.7(2), C(3)–Re(1)–N(2) 175.30(19), C(1)–Re(1)–C(2) 89.7(2), C(1)–Re(1)–C(3) 86.0(2), C(2)–Re(1)–C(3) 91.0(2), C(1)–Re(1)–Cl(1) 91.57(18), C(2)–Re(1)–Cl(1) 177.71(18), C(3)–Re(1)–Cl(1) 90.94(17), N(1)–Re(1)–Cl(1) 81.05(10), N(2)–Re(1)–Cl(1) 85.31(10) (see also Table S2).

The complex **1** crystallizes in the *P*2_1_/c space group of the monoclinic system (Table S1). The asymmetric unit of **1** consists of two crystallographically independent molecules (Figure 1), showing negligible variations in bond lengths, bond angles, and conformations.

In each molecule, the Re(I) ion is co-ordinated with two pyridyl nitrogen atoms of *N*-methyl-piperazinyl-C_6_H_4_-terpy ligand, three *facially* arranged carbon atoms of three carbonyl groups and one chloro ligand, giving a distorted octahedral geometry. The uncoordinated pyridine ring is rotated by 49.83° and 62.43° from the plane of the central pyridine ring for Re(1) and Re(2), respectively. All the bond lengths and angles around the Re atom are comparable to those of the reported structurally related compounds [27,28,52,66,68,69,70,71,72,73]. Additional structural data are given in ESI, Tables S3–S5, and Figure S1.

The facial arrangement of carbonyl ligands in **1** and **2** is also corroborated by solid-state IR spectroscopy (Figures S2 and S3). Both complexes display the characteristic pattern of CO-stretching frequencies for *fac*-geometry, represented by an intense band at higher wavenumbers (2018 cm^−1^ for **1** and 2017 cm^−1^ for **2**) and two overlapping lower-energy bands (1922–1879 cm^−1^). 

In agreement with the κ^2^N coordination of 4′-(4-substituted-phenyl)-terpyridine ligand, the ^1^H NMR spectra of **1** and **2** complexes display two sets of signals for protons of the peripheral pyridine rings. Detailed assignment of signals to respective atoms was based on the recorded ^1^H-^1^H COSY, ^1^H-^13^C HMQC, and ^1^H-^13^C HMBC NMR spectra (Figures S4–S17).

### 2.2. Photophysical Behavior—Experimental and Theoretical Insights

**Figure 2:**
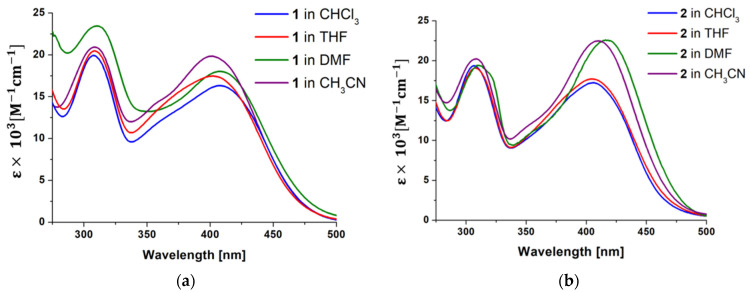
UV-Vis spectra of **1** (a) and **2** (b) in solvents of different polarity.

Absorption spectra of Re(I) complexes were studied in chloroform, tetrahydrofuran, acetonitrile, and dimethylformamide (Table 1 and Figure S22). UV-Vis spectra of **1** and **2** are shown in Figure 2, and their relevant spectral data are summarized in Table 1. 

Both complexes have similar absorption profiles, showing an intraligand ^1^IL absorption band in the high-energy range 280–340 nm along with a broad and asymmetric band in the visible region, not observed in the absorption spectra of the free ligands (Figure S22) and assigned to superposition of transitions ^1^MLCT and ^1^ILCT with the reference to previous studies [17,53,55,66,67,72,74]. 

The contribution of ^1^ILCT excitations in the visible absorption of **1** and **2** is rationalized by a significant increase in the absorption intensity and bathochromic shift of the lowest energy band compared to ^1^MLCT of reference sample(**3**). In acetonitrile, the absorption maximum wavelength of **1** and **2** is shifted to longer wavelengths by 27 and 35 nm, respectively (Table S6). The molar extinction coefficients of **1** and **2** are at least six times higher than that for **3**, reaching up to 22,500 M^−^^1^·cm^−^^1^ (for **2** in CH_3_CN). Moreover, **1** and **2** do not show a negative solvatochromism, evidenced for reference **3** and typically observed for [ReX(CO)_3_(N^∩^N)] with the longest wavelength absorption band of MLCT character [5,75]. Except for **1** in CH_3_CN, the visible absorption maxima of examined Re(I) complexes occur at longer wavelengths compared to those in chloroform solutions. It is also worth emphasizing that **1** and **2** show a less pronounced red-shift of the lowest-energy absorption relative to **3** in less polar chloroform, and the lowest-energy absorption of **1** is less affected by variations in solvent polarity than that of **2**. Relative to [ReCl(CO)_3_(Me_2_N-C_6_H_4_-terpy-κ^2^N)] (**4**), the longest-wavelength absorptions of **1** and **2** are blue-shifted by 9–25 nm (Figures S23 and S24 and Table S6).

**Figure 3:**
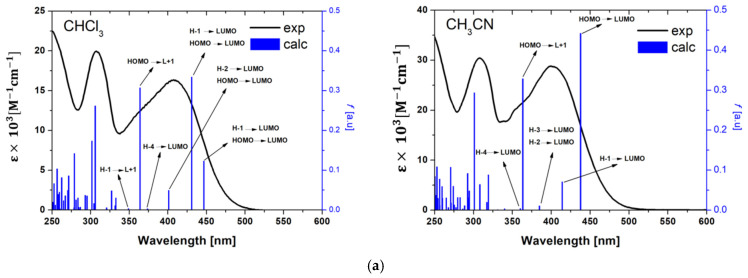

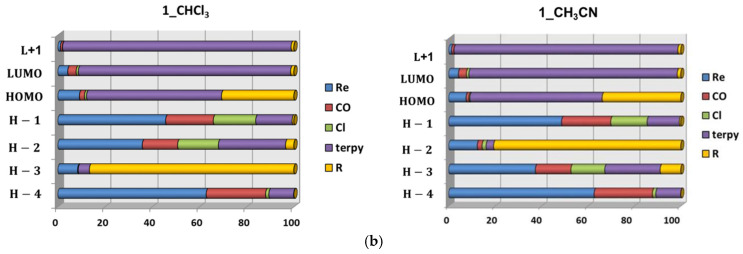
(**a**) Experimental (black line) absorption spectra of **1** alongside blue vertical lines presenting singlet–singlet transitions with corresponding oscillator strengths, computed at TD-DFT/PCM/PBE1PBE/def2-TZVPD/def2-TZVP level with the use of the PCM model at polarities corresponding to CH_3_CN and CHCl_3_; (**b**) percentage composition of molecular orbitals for compound **1** computed at the DFT/PBE1PBE/def2-TZVPD/def2–TZVP level with the use of the PCM model at polarities corresponding to CH_3_CN and CHCl_3_. For complex **2**, see Figures S25–S27 in ESI.

The nature of the electronic transitions underlying the absorption features of **1** and **2** was also investigated theoretically, with the use of the density functional theory (DFT) and time-dependent DFT (TDDFT). The calculations confirmed that the charge-transfer transitions of ILCT and MLCT character contributed to the low-energy absorption of both complexes (Figure 3 and Table S7). The excitations with the largest oscillator strengths HOMO→LUMO and HOMO→LUMO+1 are ascribed to the electron transfer from the electron-rich amine group to the electron-accepting terpy unit (^1^ILCT), admixing with those of π_terpy_→π^*^_terpy_ nature (^1^IL). The HOMO of **1** and **2** is localized on the amine group, phenyl linker, and central pyridine of the ligand, while LUMO and LUMO+1 show the largest contribution of the terpy framework with admixture of the phenyl linker (Figures S26 and S27). MLCT transitions, H-1→LUMO, H-2→LUMO, H-3→LUMO, and H-2→LUMO, are characterized by noticeably weaker oscillator strengths in agreement with poor overlapping of MOs involved in these electronic excitations—{Re(CO)_3_Cl} and π*_terpy_. Close inspection also reveals some differences between the simulations performed with the PCM model at polarities corresponding to CH_3_CN and CHCl_3_. For both complexes, the PCM model in less polar chloroform indicates stronger overlapping of ILCT and MLCT excitations and a slightly larger contribution of ^1^MLCT transitions in comparison to the PCM model in acetonitrile (Figure 3 and Figures S25–S27 and Tables S7–S9). 

**Figure 4:**
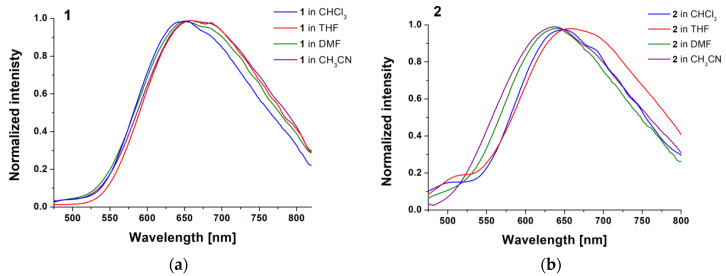
Normalized photoluminescence spectra of **1** (**a**) and **2** (**b**) in different environments.

**Figure 5:**
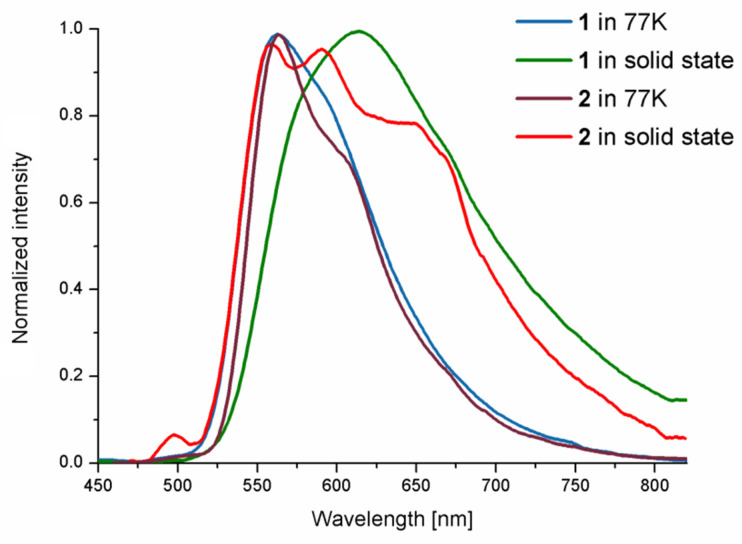
Normalized photoluminescence spectra of **1** and **2** in ethanol–methanol rigid-glass matrix (77 K) and solid state.

Room-temperature emission of **1** and **2** was studied in solvents of different polarities and solid-state (as a powder) upon excitation at the lowest energy absorption band. In addition, photoluminescence properties of **1** and **2** were investigated in an ethanol–methanol rigid-glass matrix at 77 K. Relevant photophysical parameters are summarized in Table 2 and Figure 4, Figure 5 and Figures S28–S33.

In solutions, both Re(I) complexes showed a broad and structureless emission band with maximum in the range of 640–665 nm. In general, the phosphorescence of **2** occurred at slightly shorter wavelengths in comparison to **1**, while the triplet emission of the latter one fell in the range comparable to the model chromophore (**3**). Relative to the reference compound with *N*,*N*-dimethylamine group (**4**), the emission of the designed complexes in polar solvent appeared at higher energies, while in CHCl_3_, the opposite trend was observed (Figure S29).

**Figure 6:**
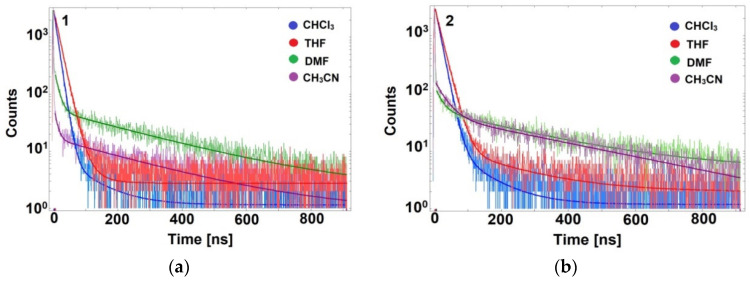
TCSPC decay curves for compounds **1** (**a**) and **2** (**b**) in different solvents.

By analogy to the complex with *N*,*N*-dimethylamine group (**4**), the emission lifetimes of **1** and **2** were strongly affected by solvent polarity, increasing noticeably upon changing the solvent from CHCl_3_ or THF to more polar CH_3_CN and DMF (Figure 6 and Table 2). Most remarkably, the incorporation of *N*-methyl-piperazinyl and (2-cyanoethyl)methylamine led to a significant enhancement of excited-state lifetimes of **1** and **2** relative to the parent chromophore (**3**) in solvents of high polarity. In DMF solution, the complex **2** showed 80-fold enhancement of the excited-state lifetime in comparison to **3** (Figure S30–32, Table S10). 

With reference to previous results [55,66,72], it can be postulated that title complexes possess two closely lying excited states ^3^MLCT and ^3^ILCT/^3^IL, and the second one becomes dominant in polar environments. 

The coexistence of ^3^MLCT and ^3^ILCT/^3^IL was further supported by photoluminescence studies of **1** and **2** in rigid environments (Figure 5). Upon cooling, the emission of **1** and **2** shifted toward higher energy and showed significantly elongated excited-state lifetimes relative to room temperature, which is consistent with the rigidochromic effect and implies the contribution of ^3^MLCT [1,2,5,18,76,77]. On the other hand, typical of the contribution of the ligand-centered emission, the frozen-state emission of **1** and **2** appeared in a noticeably lower energy region and showed weak vibronic structure and noticeably prolonged luminescence lifetime in relation to the emission of the model ^3^MLCT chromophore **3** at 77 K (Figures S31 and 32, Table S10). The average frozen-emission lifetimes of complexes **1** and **2** were two orders of magnitude longer than that for **3** at 77 K, consistent with a larger contribution from the ligand-centered state [2,5,18,76,78,79,80]. The mixed ^3^MLCT/^3^IL character of the excited state at low temperature for **1** and **2** is also supported by the comparison of their emission at 77 K with the phosphorescence spectra of the free ligands. As shown in Figure S33, the frozen-state emission of **1** and **2** overlapped the phosphorescence of the free ligand to some extent. In general, however, the emission of **1** and **2** at 77 K appeared at lower energy relative to phosphorescence measured for appropriate free ligand.

**Figure 7:**
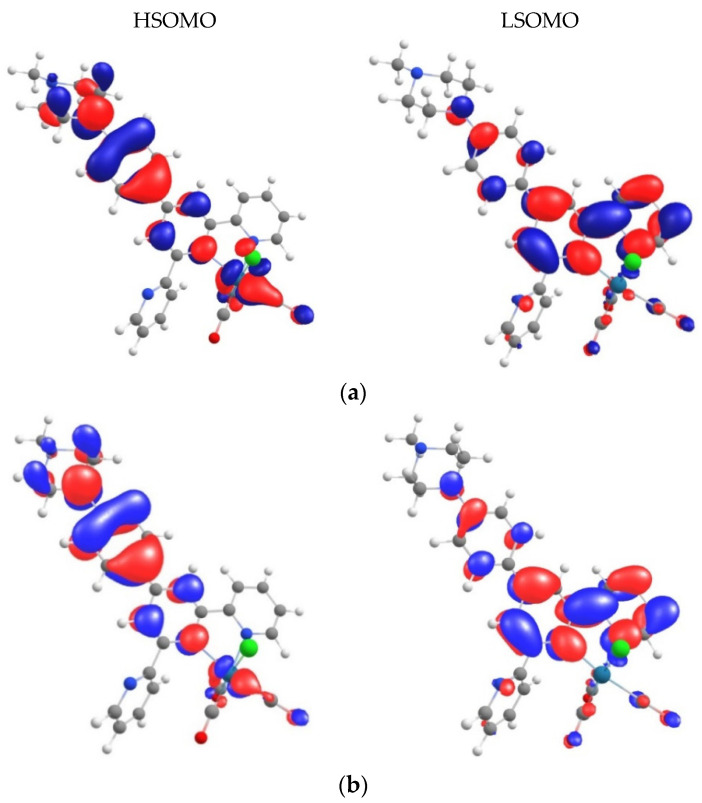
Representative isodensity surface plots of the LSOMO and HSOMO for complex **1** (TD-DFT at T_1_ state geometry, red and blue colors show regions of excess α spin density and excess β spin density values, respectively), simulated with the PCM model in chloroform (**a**) and acetonitrile (**b**). For complex **2**, see Figure S34 in ESI.

To obtain better insight into the nature of the triplet excited state of rhenium(I) complexes bearing *N*-methyl-piperazinyl and (2-cyanoethyl)methylamine, TDDFT calculations based on the optimized triplet excited-state geometries of complexes were performed. The calculations performed with the use of the PCM model in acetonitrile and chloroform provide satisfactory agreement with experimental triplet emission energies (Table S11). The isodensity surface plots of LSOMO and HSOMO and isosurfaces demonstrating the differences between α and β spin densities clearly indicate that the emitting states of **1** and **2** have predominant amine→terpy charge-transfer (ILCT) character (Figure 7 and Figure S34). However, TDDFT results also support some degree of MLCT character in the transition associated with populating the triplet state, and the contribution of ^3^MLCT increases noticeably in less polar chloroform.

### 2.3. Transient Absorption Spectroscopy

To further understand the triplet excited-state characteristics of **1** and **2**, the femtosecond transient absorption (fsTA) spectra at delay times up to 7 ns were recorded and analysed with the use of Surface Xplorer (Ultrafast Systems) and Optimus^TM^ software (version 3.02) [81,82]. The studies were performed for **1** and **2** in CHCl_3_ and CH_3_CN upon excitation 355 nm, leading to population of both ^1^IL/^1^ILCT and ^1^MLCT states. The experimental conditions were optimized on the basis of the fluence dependence tests (Figure S35).

**Figure 8:**
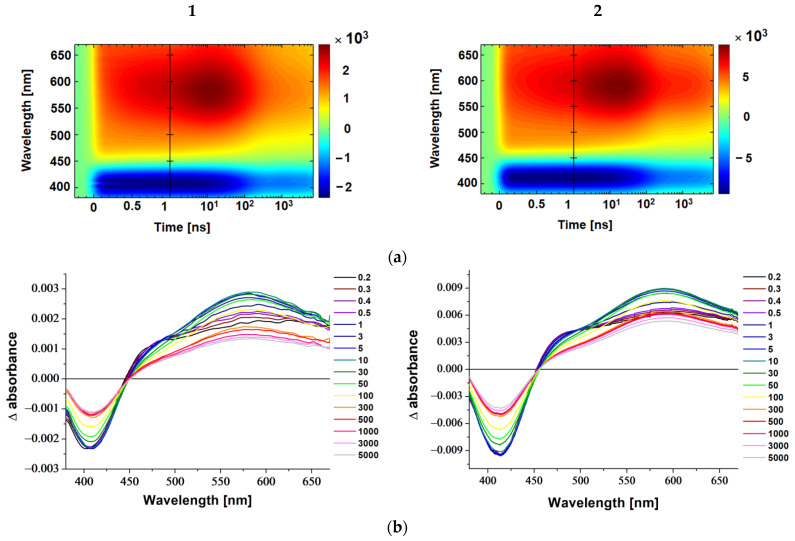
The fsTA 2D maps (**a**) and TA spectra at selected time delays (**b**) for complexes **1** and **2** in acetonitrile.

Laser pulse excitation of **1** and **2** in CH_3_CN led to the instant formation of a ground-state bleaching (GSB) ranging from 380 to 450 nm and an excited-state absorption (ESA) between 450 and 650 nm with a clear maximum at 584 nm for **1** and 589 for **2**. Both negative and positive signals remained visible up to the end of the delay stage. The GSB reflected the shape of the ground-state absorption bands of **1** and **2** well (Figure 8 and Figure S36). Within 0.2–10 ps, the ESA band of both complexes increased and sharpened in the range 450–520 nm. At longer time delays (above 30 ps), TA spectra of **1** and **2** showed strong resemblance to those of the reference compound (**4**) with *N*,*N*-dimethylamine group (Figure S37), for which ^3^ILCT character of the lowest triplet state was confirmed by time-resolved IR spectroscopy [55]. In contrast, the parent complex **3** displayed only positive signals across the entire wavelength range (Figure S38). Therefore, we can safely postulate that the lowest triplet excited state of **1** and **2** is of predominant ILCT nature, and the well-defined isosbestic point at 489 nm for **1** and 498 nm for **2** represents interconversion between the ^3^MLCT and ^3^ILCT excited states.

**Figure 9:**
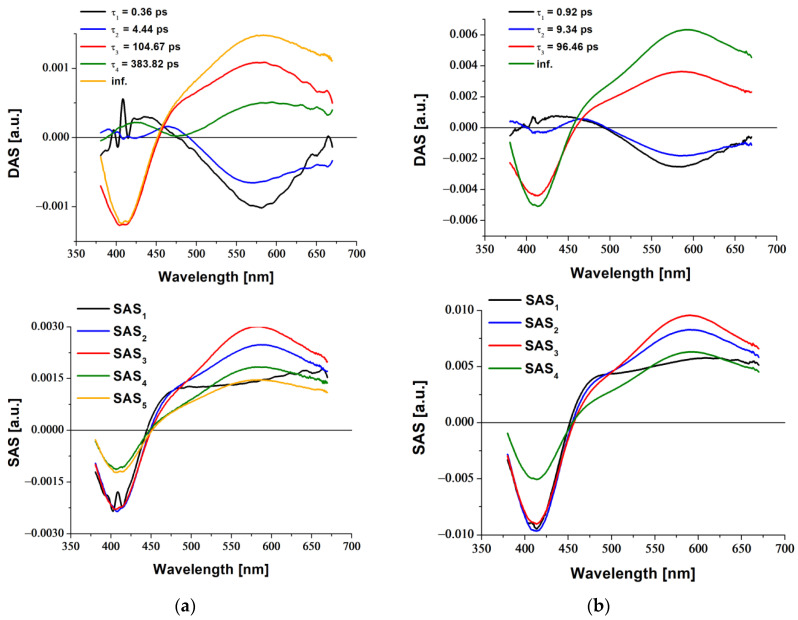
Decay associated spectra (DAS_i_) with corresponding time constants (t_i_) along with species associated spectra (SAS_i_) for complexes **1** (**a**) and **2** (**b**) in acetonitrile.

The global fit analysis of the fsTA data of **1** and **2** in CH_3_CN allows us to assume that the long-lived state ^3^IL/^3^ILCT is populated via two paths represented by decay-associated spectra which are negative in the region corresponding to the relaxed lowest triplet state ^3^IL/^3^ILCT, that are DAS_1_ and DAS_2_ with time constants t_1_ = 0.36 ps and t_1_ = 4.44 ps for **1** and t_1_ = 0.92 ps and t_1_ = 9.34 ps for **2**, respectively (Figure 9). Most likely, the formation of ^3^ILCT is associated with processes ^1^MLCT → ^3^MLCT → ^3^ILCT/^3^IL and ^1^ILCT/^1^IL → ^1^MLCT → ^3^MLCT→ ^3^ILCT/^3^IL. The interconversion ^3^MLCT → ^3^ILCT is represented by DAS_3_. The species associated spectrum SAS_1_ and SAS_2_ intersect with SAS_3_ (Figure 9). The formed hot ^3^ILCT excited state undergoes the vibrational relaxation, which comprises reorganization within the chromophores [ReCl(CO)_3_(N-methyl-piperazinyl-C_6_H_4_-terpy-κ^2^N)] (**1**) and [ReCl(CO)_3_((CH_3_)(CH_2_CH_2_CN)N-C_6_H_4_-terpy-κ^2^N)] (**2**) and between solute and solution molecules. The fully relaxed excited state recovers to the ground state, which is represented by DAS_5_ for **1** and DAS_4_ for **2**, with the infinite lifetimes. An additional component in the decay associated spectra of **1** compared to **2** may indicate the presence of two conformational forms of ^3^ILCT excited states, differing in relative orientation of aliphatic cyclic amine (*N*-methyl-piperazinyl) towards terpy moiety. Additionally, complexes **1** and **2** differ in excited-state dynamics, supporting a crucial role of the remote amine group in controlling photophysical behavior of [ReCl(CO)_3_(R-C_6_H_4_-terpy-κ^2^N)]. Formation of ^3^ILCT was much slower in the case of complex **2**. In turn, the population of ^3^ILCT was the fastest in the reference complex **4**. 

**Figure 10:**
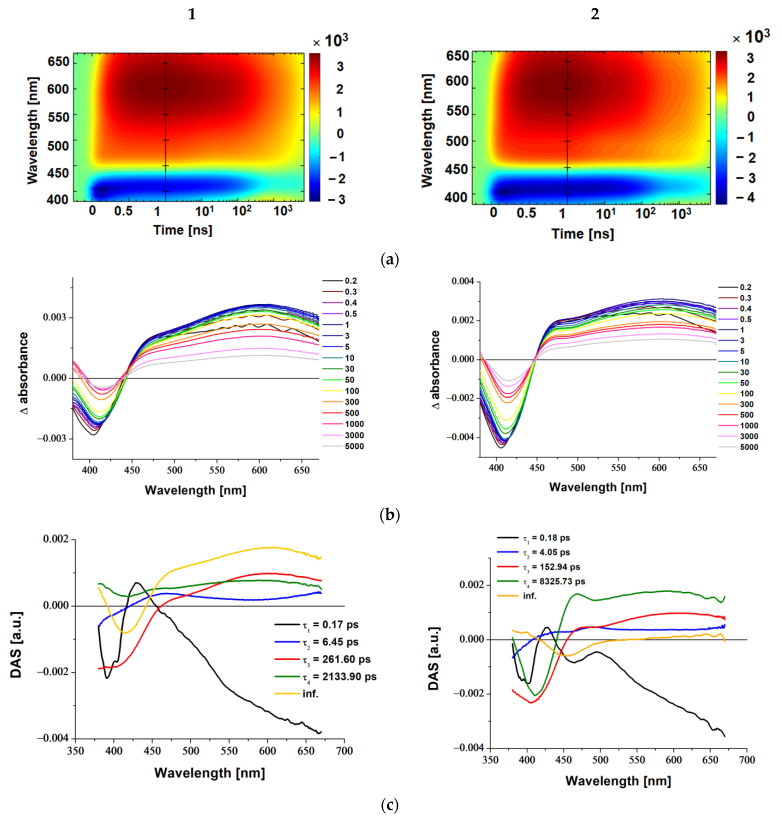

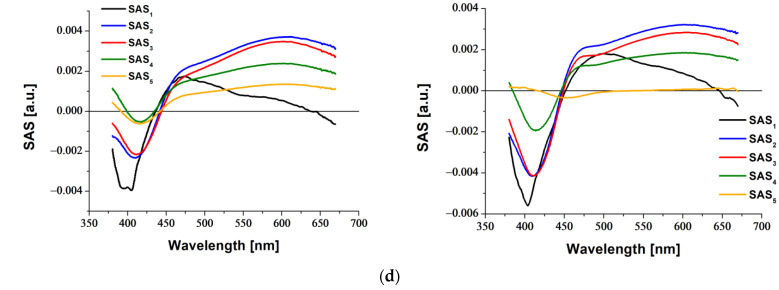
The fsTA 2D maps (**a**) and TA spectra at selected time delays (**b**), decay associated spectra (DAS_i_) (**c**), and species associated spectra (SAS_i_) (**d**) for complexes **1** and **2** in chloroform.

The fsTA 2D maps, TA spectra at selected time delays, decay associated spectra (DAS_i_), and species associated spectra (SAS_i_) of **1** and **2** compounds in CHCl_3_ are shown in Figure 10.

In chloroform, excitation of **1** and **2** at 355 nm results in an instant appearance of a ground-state bleaching (GSB) along with excited-state absorption (ESA). However, close inspection of spectral features indicates that the excited-state dynamics occur differently in less polar CHCl_3_. For both **1** and **2**, there is no isosbestic point representing interconversion between the ^3^MLCT and ^3^ILCT excited states, the transient absorption spectra in the range 450–650 nm remain broad with two distinguishable peaks at around 472 and 601 nm for **1** and 475 and 602 nm for **2**, and the spectral shapes of the corresponding DAS_i_ do not resemble those obtained in CH_3_CN. Furthermore, the global fit analysis of the fsTA data of **1** and **2** in CHCl_3_ confirms the presence of two long-lived components, with lifetimes ~2.1 ns and infinite for **1**, and ~8.3 ns and infinite for **2**. The emissive state of **1** and **2** in less polar environments seems to be dominated by the ^3^MLCT excited-state. 

Remarkably, TA spectra, decay associated spectra (DAS_i_), and species associated spectra (SAS_i_) of the reference complex with *N*,*N*-dimethylamine group (**4**) in CHCl_3_ (Figure S37) have some similar features to those of **1** and **2** in CH_3_CN, indicating some energy transfer between the organic and inorganic components even in less polar chloroform. Therefore, the stronger electron-donating group is incorporated into terpy-based ligands of [ReCl(CO)_3_(R-C_6_H_4_-terpy-κ^2^N)], the more probable switching of ^3^MLCT into ^3^ILCT there is. 

## 3. Materials and Methods

### 3.1. Materials and General Information

Re(CO)_5_Cl, starting materials and solvents (of reagent grade) for ligands synthesis, as well as solvents for spectroscopic studies (of HPLC grade) were commercially available and were used without further purification. The ligands *N*-methyl-piperazinyl-terpy and (2-cyanoethyl)methylamine-terpy were prepared according to literature methodology based on the condensation between 2-acetylpyridine and suitable benzaldehyde, 4-(4-methylpiperazin-1-yl)benzaldehyde for L^1^ and 4-[(2-cyanoethyl)methylamino]benzaldehyde for L^2^, in the presence of KOH and NH_3_ [64]_._ FT-IR, NMR, and HRMS spectra of free ligands L^1^ and L^2^ are provided in Supporting Information (ESI). 

Elemental analysis was recorded on a Vario EL Cube apparatus. FT-IR spectra were collected on Nicolet iS5 FTIR spectrophotometer (4000–400 cm^−1^) in the form of KBr pellets. ^1^H and ^1^H-^1^H COSY NMR spectra were recorded on a Bruker Avance (400 MHz) spectrometer, while ^13^C, ^1^H-^13^C HMQC, and ^1^H-^13^C HMBC spectra were collected on a Bruker Avance (500 MHz) spectrometer. Chemical shifts were referenced with respect to solvent residual signal.

### 3.2. Preparation of [ReCl(CO)_3_(N-Methyl-piperazinyl-C_6_H_4_-terpy-κ^2^N)] (***1***) and [ReCl(CO)_3_((CH_3_)(CH_2_CH_2_CN)N-C_6_H_4_-Terpy-κ^2^N)] (***2***)

Rhenium(I) carbonyl complexes were prepared using Re(CO)_5_Cl (0.10 g, 0.27 mmol) and corresponding L^1^ and L^2^ ligand (0.27 mmol) in the substitution reaction of two carbonyl groups of [Re(CO)_5_Cl] by N-donor atoms of terpy-based ligands [27,66,67,68,72,73,83]. Argon-saturated acetonitrile solution of pentacarbonyl chlororhenium and corresponding ligand was refluxed for 8 h. The resulting solution was left for slow evaporation at room temperature. The formed precipitate was filtered off and washed with diethyl ether, dried in the air, and then purified by repeated recrystallization from acetonitrile. The product purity was confirmed by thin-layer chromatography analyses (SiO_2_, 5–10% MeOH in CH_2_Cl_2_).

**1**: Yield: 85%. IR (KBr, cm^−1^): 2017 (vs.), 1922 (vs.) and 1879 (vs.) ν(C≡O); 1599 (m), 1527 (m) ν (C=N) and ν (C=C). ^1^H NMR (400 MHz, DMSO-*d*_6_) δ 9.10 (d, *J* = 8.3 Hz, 1H), 9.05 (d, *J* = 5.4 Hz, 1H), 8.99 (s, 1H), 8.79 (d, *J* = 4.3 Hz, 1H), 8.38 (t, *J* = 7.9 Hz, 1H), 8.13 (d, *J* = 8.9 Hz, 2H), 8.08 (s, 1H), 8.04 (t, *J* = 8.1 Hz, 1H), 7.88 (d, *J* = 7.7 Hz, 1H), 7.77 (t, *J* = 6.6 Hz, 1H), 7.62 (dd, *J* = 7.7, 5.0 Hz, 1H), 7.11 (d, *J* = 8.6 Hz, 2H), 3.38–3.34 (m, 4H), 2.49–2.45 (m, 4H), 2.25 (s, 3H) ppm. ^13^C NMR (125 MHz, DMSO-*d*_6_) δ 197.89, 194.55, 191.17, 161.12, 158.09, 156.78, 156.52, 152.63, 152.55, 150.12, 149.18, 139.85, 136.86, 128.87, 127.27, 125.29, 125.10, 124.81, 123.12, 122.39, 118.83, 114.57, 54.15, 46.49, 45.40 ppm.

Anal. calc. found: C 47.31; H 3.56; N 9.80; molecular formula [ReCl(CO)_3_(C_26_H_25_N_5_)]·H_2_O requires C 47.63; H 3.72; N 9.58%. HRMS (ESI) (*m*/*z*): [M + H]^+^ calcd for [C_29_H_25_N_5_O_3_Re]^+^ 678.1515. Found 678.1518.

**2**: Yield: 75%. IR (KBr, cm^−1^): 2017 (vs.), 1915 (vs.) and 1880 (vs.) ν (C≡O); 1599(s) and 1532 (m) ν(C=N) and ν (C=C). ^1^H NMR (400 MHz, DMSO-*d*_6_) δ = 9.10 (d, *J* = 8.3 Hz, 1H), 9.04 (d, *J* = 5.5 Hz, 1H), 8.97 (s, 1H), 8.78 (d, *J* = 4.9 Hz, 1H), 8.37 (t, *J* = 7.9 Hz, 1H), 8.13 (d, *J* = 8.6 Hz, 2H), 8.07 (s, 1H), 8.04 (t, *J* = 7.5 Hz, 1H), 7.87 (d, *J* = 7.8 Hz, 1H), 7.76 (t, *J* = 6.6 Hz, 1H), 7.61 (dd, *J* = 7.6, 5.0 Hz, 1H), 6.95 (d, *J* = 8.6 Hz, 2H), 3.82 (t, *J* = 6.8 Hz, 2H), 3.08 (s, 3H), 2.79 (t, *J* = 6.6 Hz, 2H) ppm. ^13^C NMR (125 MHz, DMSO-*d*_6_) δ = 197.90, 194.56, 191.18, 161.05, 158.15, 156.69, 156.57, 152.61, 150.37, 150.27, 149.15, 139.83, 136.83, 129.05, 127.22, 125.24, 125.08, 124.76, 122.09, 121.46, 119.50, 118.59, 112.30, 47.23, 37.92, 15.08 ppm. Anal. calc. found: C 47.41; H 3.48; N 10.27; molecular formula [ReCl(CO)_3_(C_28_H_21_N_5_)]·0.5H_2_O requires C 47.62; H 3.14; N 9.92%. HRMS (ESI) (*m*/*z*): [M + H]^+^ calcd for [C_28_H_21_N_5_O_3_Re]^+^ 662.1202. Found 662.1204.

### 3.3. X-ray Crystallography

Single crystals of **1** were obtained from acetonitrile by slow evaporation of the solvent at room temperature. The X-ray diffraction was collected using Oxford Diffraction four-circle diffractometer Gemini A Ultra with Atlas CCD detector using graphite monochromated MoKα radiation (λ = 0.71073 Å) at room temperature. Data were processed with the aid of CrysAlis^Pro^ software [84], Olex2 software [85], SHELXS, and SHELXL-2014 package [86]. All the non-hydrogen atoms were refined anisotropically, and hydrogen atoms were placed in calculated positions and refined with riding constraints: *d*(C–H) = 0.93 Å, *U*_iso_(H) = 1.2 *U*_eq_(C) (for aromatic) and *d*(C–H) = 0.96 Å, *U*_iso_(H) = 1.5 *U*_eq_(C) (for methyl). Details of the crystallographic data collection, structural determination, and refinement for **1** are given in Table S1, whereas selected bond lengths and angles are listed in Table S2, ESI.

Crystallographic data for **1** were deposited with the Cambridge Crystallographic Data Center, CCDC 2205501. Copies of this information may be obtained free of charge from the Director, CCDC, 12 Union Road, Cambridge CB2 1EZ, UK (Fax: +44 1223 336033; e-mail: deposit@ccdc.cam.ac.uk or www.ccdc.cam.ac.uk (accessed on 6 September 2022)).

### 3.4. Absorption and Emission Spectra

The UV-Vis spectra were measured on ThermoScientific Evolution 220 (Waltham, MA, USA) (solution) and Jasco V570. Steady-state emission spectra of solid state (as powder) and solution samples were recorded on the FLS-980 fluorescence spectrophotometer, equipped with a 450 W Xe lamp and high-gain photomultiplier PMT + 500 nm (Hamamatsu, R928P) detector. The emission spectra at 77 K were registered in an ethanol:methanol (4:1 *v*/*v*) matrix frozen with liquid nitrogen. To determine the PL lifetimes, correlated single photon counting (TCSPC) and multi-channel scaling (MCS) methods were used. The TCSPC measurements were carried out at optically diluted solutions using the diodes (EPLED 375 nm, EPLED 405 nm) with picosecond pulse period as excitation light sources and PMT (Hamamatsu, R928P, Japan) as a detector. The IRF was measured using ludox solution. For samples with long-lived phosphorescence, PL lifetime measurements were performed with a multi-channel scaling (MCS) method in which excitation wavelength was obtained using 60 W microsecond Xe flash lamp. The quantum yields of luminescence were calculated by absolute method using the integrating sphere with solvent (for argon-saturated solution samples) or Spectralon^®^ reflectance standard (for powdered samples) as blanks. 

### 3.5. Femtosecond Transient Absorption

The femtosecond TA spectra were measured using a pump-probe transient absorption spectroscopy system (Ultrafast Systems, Helios, Sarasota, FL, USA) described in our previous works [64,87]. All experiments were carried out for the solution samples (2.5 × 10^−4^ M, in CHCl_3_ and CH_3_CN), stirred during the experiments to avoid photoproduct interference. The absorbance range was about 0.50 in the excitation wavelengths. The samples were pumped with 355 nm (for **1** and **2**). Obtained TA data were analyzed using the Surface Xplorer (Ultrafast Systems) and Optimus software [82]. Corrections for the probe chirp and solvent signal contributions were performed routinely prior to the analysis. The used software allowed us to perform singular value deconvolution of the 3D surface into principal components (spectra and kinetics), global analysis and decay associated spectra, DAS, of the detected transients.

### 3.6. Theoretical Calculations

The theoretical calculations were performed using the GAUSSIAN-09 program package [88] at the DFT level with the PBE1PBE [89,90] hybrid exchange-correlation functional and the def2-TZVPD basis set for rhenium and def2-TZVP basis set for other elements [91] with polarizable continuum model (PCM) and acetonitrile as solvent [92,93]. Following the optimization of the geometry, vibrational frequencies were calculated to verify the minimum on the potential energy surface. Absorption properties were calculated by a TD-DFT method on the basis of the optimized ground state geometries. Emission properties were calculated by means of the TD-DFT method (after optimization of the first triplet excited-state).

## 4. Conclusions

We found that remote electron-rich amine groups *N*-methyl-piperazinyl (**1**) and (2-cyanoethyl)methylamine (**2**) effectively change optical properties of Re(I) complexes [ReCl(CO)_3_(R-C_6_H_4_-terpy-κ^2^N)]. As a result of the contribution of intraligand charge-transfer (ILCT) transitions, both new complexes show a red shift and significant increase in absorption intensity of the lowest energy band compared to ^1^MLCT of the parent compound [ReCl(CO)_3_(C_6_H_5_-terpy-κ^2^N)] (**3**). The photoluminescence properties of **1** and **2** strongly vary depending on the solvent polarity. In polar solvents, phosphorescence lifetimes of both complexes are significantly prolonged in relation to the model chromophore. On the basis of femtosecond transient absorption (fsTA) studies, we found that the ^3^MLCT excite state of **1** and **2** in polar environments undergoes a conversion into ^3^ILCT one. In contrast, the photoluminescence properties of **1** and **2** in less polar environments seem to be determined by ^3^MLCT excited-state. By comparing spectral features of designed complexes with the reference complex [ReCl(CO)_3_(R-C_6_H_4_-terpy-κ^2^N)] bearing *N*,*N*-dimethylamine group (**4**), it can be concluded that the stronger electron-donating group in **4** facilitates the population of the ^3^ILCT excited-state in both polar and less polar solvents. These findings can help us design new luminescent materials and understand the factors that control the excited-state nature in transition metal complexes. 

## Figures and Tables

**Table 1:** The absorption maxima and molar extinction coefficient for **1** and **2** in solvents of different polarity.

Compound	λ/nm (10^4^ ε/dm^3^·mol^−1^·cm^−1^)			
	CHCl_3_	THF	CH_3_CN	DMF
**1**	405 (1.63)	405 (1.75)	402 (1.93)	409 (1.66)
**2**	405 (1.72)	406 (1.78)	405 (2.24)	417 (2.26)

**Table 2:** Relevant photophysical parameters of complexes **1** and **2**.

Compound	Medium	λ_ex_ [nm]	λ_em_ [nm]	τ*_av_*	Φ
**1**	CHCl_3_	405	645	8 ns	0.009
	THF	405	665	6 ns	0.010
	DMF	409	650	248 ns	0.001
	CH_3_CN	405	665	146 ns	<0.001
	77 K (EtOH-MeOH)	420	560	178 μs	––
	solid	475	613	553 ns	0.071
**2**	CHCl_3_	430	643	8 ns	0.026
	THF	445	665	6 ns	0.027
	DMF	420	640	579 ns	0.003
	CH_3_CN	415	640	79 ns	0.003
	77 K (EtOH-MeOH)	410	564	173 μs	–
	solid	450	560,589,649	828 ns	0.003

## Data Availability

Not applicable.

## Supplementary Materials

The following supporting information can be downloaded at: https://www.mdpi.com/article/10.3390/molecules27217147/s1, Table S1: Crystal data and structure refinement for **1**; Table S2: Bond lengths and angles for **1**; Table S3: Short intramolecular contacts detected in the structure of **1**; Table S4: Short π⋯π interactions for **1**; Table S5: C—O⋯Cg(J) (π-ring) interactions of **1**. Figure S1: View of supramolecular packing of **1** arising from weak π⋯π type and C–O⋯π interactions and C–H⋯Cl short contacts; Figure S2: FT-IR spectrum of **1** along with FT-IR spectrum of the free ligand; Figure S3: FT-IR spectrum of **2** along with FT-IR spectrum of the free ligand; Figure S4: ^1^H NMR spectrum of L_1_; Figure S5: ^13^C NMR spectrum of L_1_; Figure S6: ^1^H NMR spectrum of L_2_.; Figure S7: ^13^C NMR spectrum of L_2_.; Figure S8: ^1^H NMR spectrum of **1**.; Figure S9: ^13^C NMR spectrum of **1**; Figure S10: ^1^H-^1^H COSY NMR spectrum of **1**; Figure S11: ^1^H-^13^C HMQC NMR spectrum of **1**; Figure S12: ^1^H-^13^C HMBC NMR spectrum of **1**; Figure S13: ^1^H NMR spectrum of **2**; Figure S14: ^13^C NMR spectrum of **2**; Figure S15: ^1^H-^1^H COSY NMR spectrum of **2**; Figure S16: ^1^H-^13^C HMQC NMR spectrum of **2**; Figure S17: ^1^H-^13^C HMBC NMR spectrum of **2**; Figure S18: HMRS spectrum of L_1_; Figure S19: HMRS spectrum of L_2_; Figure S20: HMRS spectrum of **1**; Figure S21: HMRS spectrum of **2**; Figure S22: UV-Vis spectra of **1** and **2** recorded once every two hours over 12 h at room temperature; Figure S23: UV−Vis spectra of **1** and **2** in comparison to those for free ligands; Figure S24: UV−Vis spectra of **1** and **2** in comparison to those for **3** and **4**; Table S6: The absorption maxima and molar extinction coefficient for **1** and **2** with spectral data for **3** and **4**; Table S7: The energies and characters of spin-allowed electronic transitions assigned to the lowest wavelength absorption bands of **1** and **2**; Figure S25: Experimental absorption spectra of **2** alongside with vertical lines presenting singlet-singlet transitions with corresponding oscillator strengths; Figure S26: Percentage composition of molecular orbitals for **1** and **2** (in CHCl_3_); Figure S27: Percentage composition of molecular orbitals for **1** and **2** (in CH_3_CN); Table S8: Selected molecular orbitals of **1**; Table S9: Selected molecular orbitals of **2**; Figure S28: Normalized emission spectra of **1** and **2** in comparison to those for free ligands; Figure S29: Normalized luminescence spectra of **1–4**; Table S10: Relevant photophysical parameters of **1** and **2** in comparison to those for **3** and **4**; Figure S30: TCSPC decay curves for **3** and **4** in different solvents; Figure S31: Decay curves of **1** in deaerated CHCl_3_, THF, DMF, CH3CN at room temperature, in ethanol-methanol rigid-glass matrix (77 K) and solid state; Figure S32: Decay curves of **2** in deaerated CHCl_3_, THF, DMF, CH_3_CN at room temperature, in ethanol-methanol rigid-glass matrix (77 K) and solid state; Figure S33: Normalized emission spectra of the free ligands and their Re(I) complexes in ethanol-methanol rigid-glass matrix (77 K); Table S11: Calculated phosphorescence emission energies of **1** and **2**, compared to the experimental values recorded in acetonitrile solution; Figure S34: Representative isodensity surface plots of the LSOMO and HSOMO for **2**; Figure S35: The results of fluence dependence tests of **1** and **2**; Figure S36: TA spectra at selected time delays and time traces at several wavelength for **1** and **2** in chloroform and acetonitrile; Figure S37: The fsTA 2D maps (a) and TA spectra at selected time delays; (b and f) decay associated spectra (DASi); (c) species associated spectra (SASi); (d) time traces at several wavelength; (e) for **4** in chloroform and acetonitrile; Figure S38: The fsTA 2D maps (a) and TA spectra at selected time delays (b and f)) decay associated spectra (DASi) (c), species associated spectra (SASi) (d) and time traces at several wavelength (e) for **3** in chloroform and acetonitrile.

## Sample Availability

Samples of the compounds **1** and **2** are available from the authors.
