# Supplementary material for: Photoinduced Processes in Rhenium(I) Terpyridine Complexes Bearing Remote Amine Groups: New Insights from Transient Absorption Spectroscopy
Source: Molecules. 2022 Oct 22;27(21):7147. doi: 10.3390/molecules27217147 (PMC9656794; doi:10.3390/molecules27217147)
Supplement: Supplementary file 1 [file molecules-27-07147-s001.zip › molecules-1933502-supplementary.pdf]

Supplementary Information

Photoinduced processes in rhenium(I) terpyridine complexes bearing remote amine groups. New insights from transient absorption spectroscopy

Joanna Palion-Gazda[a*], Agata Szłapa-Kula[a], Mateusz Penkala[a], Karol Erfurt[b] and Barbara Machura[a*]

[a]*Institute of Chemistry, University of Silesia, Szkolna 9, 40-006 Katowice, Poland. E-mails: joanna.palion-gazda@us.edu.pl and barbara.machura@us.edu.pl*

[b]*Department of Chemical Organic Technology and Petrochemistry, Silesian University of Technology, Krzywoustego 4, 44-100 Gliwice, Poland*

Spis treści

X-Ray analysis 3

Table S1. Crystal data and structure refinement for **1**. 3

Table S2. Bond lengths [Å] and angles [°] for **1**. 3

Table S3. Short intramolecular contacts detected in the structure of **1** 4

Table S4. Short π•••π interactions for **1**. 4

Table S5. C—O•••Cg(J) (π-ring) interactions of **1**. 4

Figure S1. View of supramolecular packing of **1** arising from weak π•••π type and C–O•••π interactions and C–H•••Cl short contacts. 5

IR spectra 6

Figure S2. FT-IR spectrum of **1** along with FT-IR spectrum of the free ligand. 6

Figure S3. FT-IR spectrum of **2** along with FT-IR spectrum of the free ligand. 7

NMR spectroscopy 8

Figure S4. ^{1}H NMR spectrum of L_1. 8

Figure S5. ^{13}C NMR spectrum of L_1. 9

Figure S6. ^{1}H NMR spectrum of L_2. 10

Figure S7. ^{13}C NMR spectrum of L_2. 11

Figure S8. ^{1}H NMR spectrum of **1**. 12

Figure S9. ^{13}C NMR spectrum of **1** 12

Figure S10. ^{1}H-^{1}H COSY NMR spectrum of **1.** 13

Figure S11. ^{1}H-^{13}C HMQC NMR spectrum of **1**. 14

Figure S12. ^{1}H-^{13}C HMBC NMR spectrum of **1.** 15

Figure S13. ^{1}H NMR spectrum of **2.** 16

Figure S14. ^{13}C NMR spectrum of **2.** 16

Figure S15. ^{1}H-^{1}H COSY NMR spectrum of **2.** 17

Figure S16. ^{1}H-^{13}C HMQC NMR spectrum of **2**. 18

Figure S17. ^{1}H-^{13}C HMBC NMR spectrum of **2.** 19

HMRS spectroscopy 20

Figure S18. HMRS spectrum of **L_1.** 20

Figure S19. HMRS spectrum of **L_2.** 20

Figure S20. HMRS spectrum of **1.** 21

Figure S21. HMRS spectrum of **2.** 21

UV-VIS studies 22

Figure S22. UV-Vis spectra of **1** and **2** recorded once every two hours over 12h at room temperature. 23

Figure S23. UV−Vis spectra of **1** and **2** in comparison to those for free ligands. 23

Figure S24. UV−Vis spectra of **1** and **2** in comparison to those for [ReCl(CO)$_3$(C$_6$H$_5$-terpy-κ^{2}N)] (**3**) and [ReCl(CO)$_3$(Me$_2$N-C$_6$H$_4$-terpy-κ^{2}N)] (**4**) in CHCl$_3$ (a) and CH$_3$CN (b). 24

Table S6. The absorption maxima and molar extinction coefficient for **1** and **2** with spectral data for [ReCl(CO)$_3$(C$_6$H$_5$-terpy-κ^{2}N)] (**3**) and [ReCl(CO)$_3$(Me$_2$N-C$_6$H$_4$-terpy-κ^{2}N)] (**4**) chloroform (a), tetrahydrofurane (b), N,N–dimethylformamide (c) and acetonitrile (d). 25

Absorption properties - TDDFT calculations 25

Table S7. The energies and characters of spin-allowed electronic transitions assigned to the lowest wavelength absorption bands of **1** and **2**. 25

Figure S25. Experimental absorption spectra of **2** alongside vertical lines presenting singlet-singlet transitions with corresponding oscillator strengths. 26

Figure S26. Percentage composition of molecular orbitals for **1** and **2** (in CHCl$_3$) 26

Figure S27. Percentage composition of molecular orbitals for **1** and **2** (in CH$_3$CN). 27

Table S8. Selected molecular orbitals of **1** 27

Table S9. Selected molecular orbitals of **2** 29

Photoluminescence studies 31

Figure S28. Normalized emission spectra of **1** and **2** in comparison to those for free ligands 31

Figure S29. Normalized luminescence spectra of **1**–**4** in deaerated chloroform (a), tetrahydrofurane (b), N,N–dimethylformamide (c) and acetonitrile (d). 32

Table S10. Relevant photophysical parameters of **1** and **2** in comparison to those for [ReCl(CO)$_3$(C$_6$H$_5$-terpy-κ^{2}N)] (**3**) and [ReCl(CO)$_3$(Me$_2$N-C$_6$H$_4$-terpy-κ^{2}N)] (**4**). **Error! Bookmark not defined.**

Figure S30. TCSPC decay curves for **3** and **4** in different solvents. 33

Figure S31. Decay curves of **1** in deaerated CHCl$_3$, THF, DMF, CH$_3$CN at room temperature, in ethanol-methanol rigid-glass matrix (77 K) and solid state. 34

Figure S32. Decay curves of **2** in deaerated CHCl$_3$, THF, DMF, CH$_3$CN at room temperature, in ethanol-methanol rigid-glass matrix (77 K) and solid state. 35

Figure S33. Normalized emission spectra of the free ligands and their Re(I) complexes in ethanol-methanol rigid-glass matrix (77 K). The 36

Table S11. Calculated phosphorescence emission energies (DFT/PBE1PBE/def2-TZVPD/def2-TZVP) of **1** and **2**, compared to the experimental values recorded in acetonitrile solution. 36

Figure S34. Representative isodensity surface plots of the LSOMO and HSOMO for **2**. 37

Femtosecond transient absorption spectroscopy 38

Figure S35. The results of fluence dependence of **1** and **2**. 40

Figure 36. TA spectra at selected time delays and time traces at several wavelength for **1** and **2** in chloroform and acetonitrile. 42

Figure 37. The fsTA 2D maps (a) and TA spectra at selected time delays (b and f)) decay associated spectra (DAS$_i$) (c), species associated spectra (SAS$_i$) (d) and time traces at several wavelength (e) for **4**. 46

Figure 38. The fsTA 2D maps (a) and TA spectra at selected time delays (b and f)) decay associated spectra (DAS$_i$) (c), species associated spectra (SAS$_i$) (d) and time traces at several wavelength (e). 50

X-Ray analysis

Table S1. Crystal data and structure refinement for **1**.

	**1**
Empirical formula	$C_{29}H_{25}ClN_5O_3Re$
Formula weight	713.19
T, K	295.0(2)
Wavelength, Å	0.71073
Crystal system	Monoclinic
Space group	$P2_1/c$
Unit cell dimensions, Å and °	
a	15.2721(6)
b	11.5579(6)
c	32.1005(16)
β	102.434(5)
V, Å^3	5533.3(5)
Z	8
D_c, g cm^{-3}	1.712
Absorption coefficient, mm^{-1}	4.529
F(000)	2800.0
Crystal size, mm	0.26×0.13×0.03
θ range for data collection, °	3.43 to 29.47
Index ranges	$-18 \leq h \leq 18$
	$-13 \leq k \leq 10$
	$-38 \leq l \leq 33$
Reflections collected	24779
Independent reflections	9760 [$R_{int} = 0.0396$]
Completeness to 2θ	99.7
Min. and max. transm.	0.170 and 1.000
Data / restraints / parameters	9760/0/705
Goodness-of-fit on F^2	0.982
Final R indices [$I > 2\sigma(I)$]	
R_1	0.0337
wR_2	0.0643
R indices (all data)	
R_1	0.0518
wR_2	0.0698
Largest diff. peak and hole, e Å^{-3}	0.83 and –0.61
CCDC number	2205501

Table S2. Bond lengths [Å] and angles [°] for **1**.

**Bond lengths [Å]**		**Bond angels [°]**	
Re(1)–C(1)	1.893(6)	C(2)–Re(1)–C(1)	89.7(2)
Re(1)–C(2)	1.926(6)	C(3)–Re(1)–C(1)	86.0(2)
Re(1)–C(3)	1.927(6)	C(3)–Re(1)–C(2)	91.0(2)
Re(1)–N(1)	2.205(4)	C(1)–Re(1)–N(1)	168.86(16)
Re(1)–N(2)	2.171(4)	C(2)–Re(1)–N(1)	92.7(2)
Re(1)–Cl(1)	2.4889(13)	C(3)–Re(1)–N(1)	102.38(19)
Re(2)–C(30)	1.870(7)	C(1)–Re(1)–N(2)	96.94(19)
Re(2)–C(31)	1.902(6)	C(2)–Re(1)–N(2)	92.7(2)
Re(2)–C(32)	1.926(6)	C(3)–Re(1)–N(2)	175.30(19)
Re(2)–N(6)	2.199(4)	N(1)–Re(1)–N(2)	74.26(14)
Re(2)–N(7)	2.171(4)	C(1)–Re(1)–Cl(1)	91.57(18)
Re(2)–Cl(2)	2.4981(13)	C(2)–Re(1)–Cl(1)	177.71(18)
C(1)–O(1)	1.158(6)	C(3)–Re(1)–Cl(1)	90.94(17)
C(2)–O(2)	1.105(7)	N(1)–Re(1)–Cl(1)	81.05(10)

C(3)–O(3)	1.146(6)	N(2)–Re(1)–Cl(1)	85.31(10)
C(30)–O(4)	1.167(7)	C(30)–Re(2)–C(31)	88.8(3)
C(31)–O(5)	1.143(6)	C(30)–Re(2)–C(32)	85.7(2)
C(32)–O(6)	1.140(6)	C(31)–Re(2)–C(32)	87.2(2)
		C(30)–Re(2)–N(6)	169.82(17)
		C(30)–Re(2)–N(7)	97.3(2)
		C(31)–Re(2)–N(6)	97.03(19)
		C(31)–Re(2)–N(7)	95.93(19)
		C(32)–Re(2)–N(6)	102.88(18)
		C(32)–Re(2)–N(7)	175.68(18)
		N(6)–Re(2)–N(7)	73.82(14)
		N(6)–Re(2)–Cl(2)	81.54(10)
		N(7)–Re(2)–Cl(2)	83.77(10)
		C(30)–Re(2)–Cl(2)	92.6(2)
		C(31)–Re(2)–Cl(2)	178.57(17)
		C(32)–Re(2)–Cl(2)	93.03(19)

Table S3. Short intramolecular contacts detected in the structure of **1**.

D—H•••A	D—H	H•••A	D•••A [Å]	D—H•••A [°]
1				
C(15)–H(15)•••Cl(2)[a]	0.93	2.80	3.717(5)	169.00
C(20)–H(20)•••Cl(2)[a]	0.93	2.81	3.558(5)	138.00
C(49)–H(49))•••Cl(1)[b]	0.93	2.81	3.663(5)	153.00

Symmetry transformations used to generate equivalent atoms: (a) = 1+*x*, *y*, *z*; (b) = –1+*x*, *y*, *z*.

Table S4. Short π•••π interactions for **1**.

Cg(I)•••Cg(J)	Cg(I)•••Cg(J) [Å]	α[°]	β[°]	γ [°]	Cg(I)-Perp [Å]	Cg(J)-Perp [Å]
1						
Cg(1)•••Cg(2)[a]	3.977(3)	5.7(2)	33.99	29.71	-3.454(2)	3.297(2)

α = dihedral angle between *Cg*(I) and *Cg*(J); *Cg*(I)-Perp = Perpendicular distance of *Cg*(I) on ring J; *Cg*(J)-Perp = perpendicular distance of *Cg*(J) on ring I; β = angle *Cg*(I)→*Cg*(J) vector and normal to ring I; γ = angle *Cg*(I) →*Cg*(J) vector and normal to plane J;
Cg(1) = N(2)/C(14)/C(15)/C(16)/C(17)/C(18);
Cg(2) = C(48)/C(49)/C(50)/C(51)/C(52)/C(53);
Symmetry codes: (a) = 1+*x*, *y*, *z*.

Table S5. C—O•••Cg(J) (π-ring) interactions of **1**.

Y(I)–X(I)•••Cg(J)	Y(I)-H(I)•••Cg(J) [Å]	X(I)-Perp [Å]	γ [°]	Y(I)–X(I)•••Cg(J) [°]
1				
C(3)–O(3)•••Cg(3)	3.320(5)	3.289	7.88	85.6(4)
C(32)-O(6)•••Cg(4)	3.195(5)	-3.131	11.45	84.9(4)

γ = angle X(I)→Cg(J) vector and normal to plane J.
Cg(3) =N(8)/C(38)/C(39)/C(40)/C(41)/C(42);
Cg(4) = N(3)/C(9)/C(10)/C(11)/C(12)/C(13).

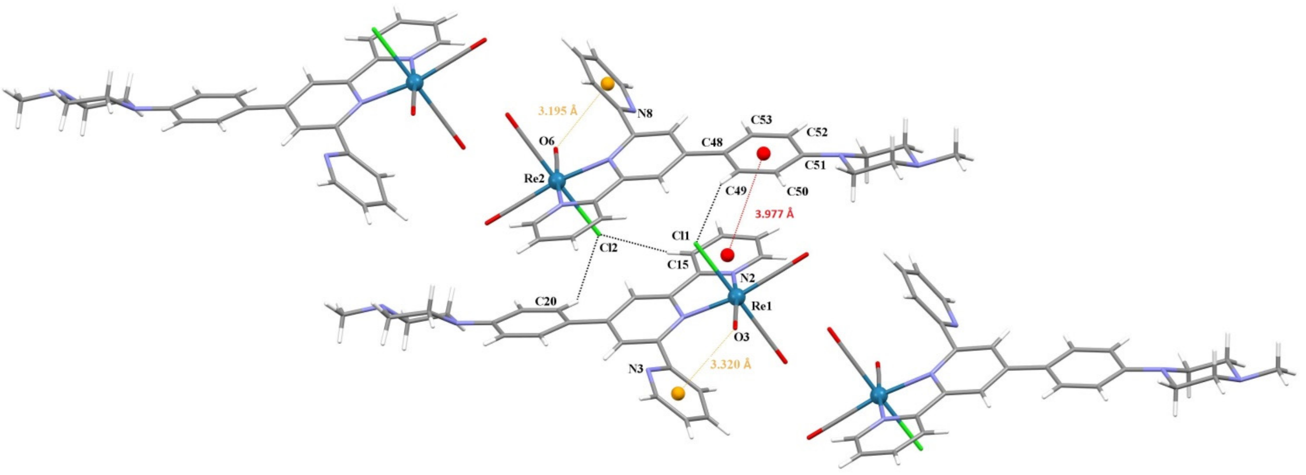

Figure S1. View of supramolecular packing of **1** arising from weak π•••π type   and C–O•••π interactions (red and yellow dashed line) and C–H•••Cl short contacts (black dashed line).

IR spectra

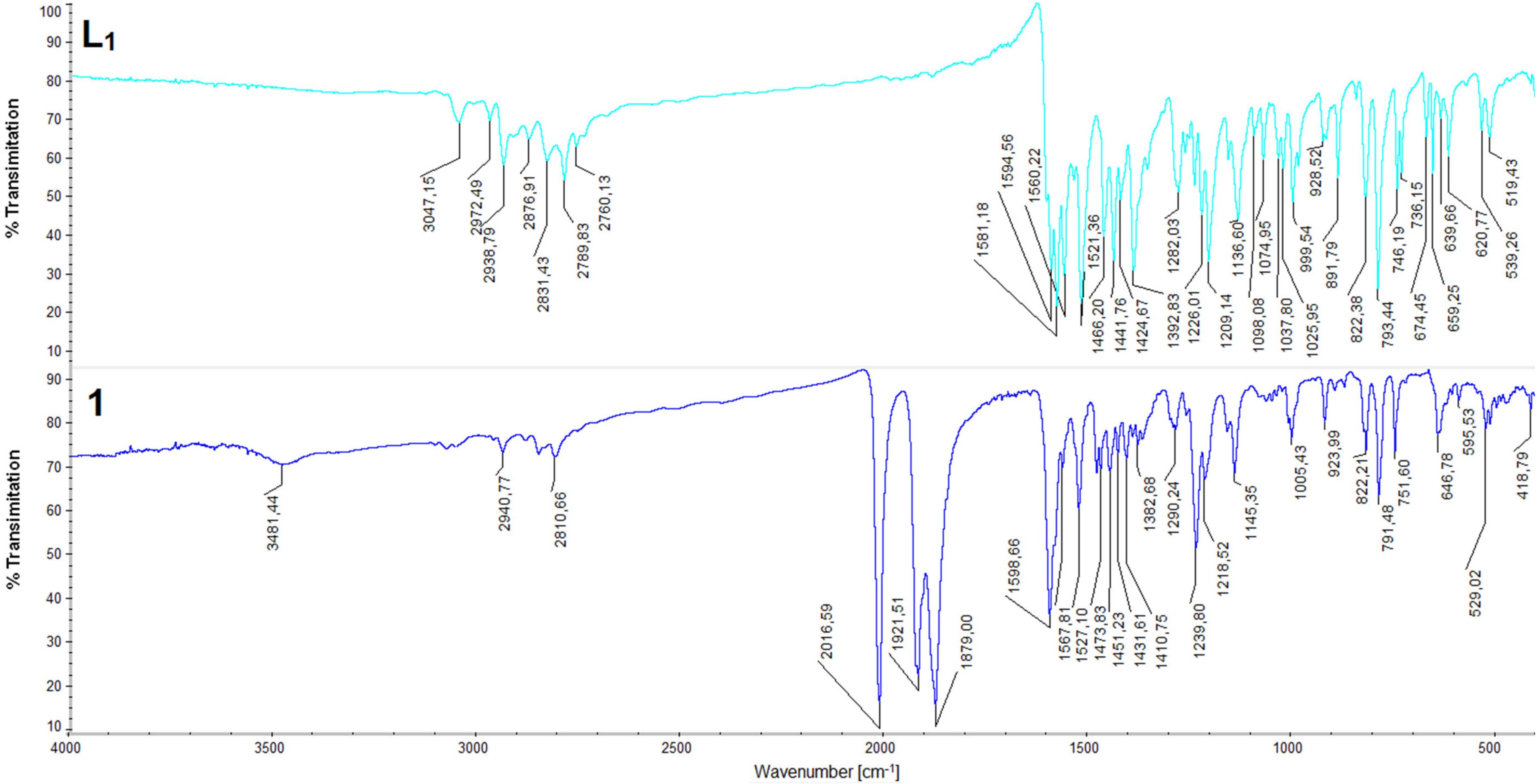

Figure S2. FT-IR spectrum of **1** along with FT-IR spectrum of the free ligand.

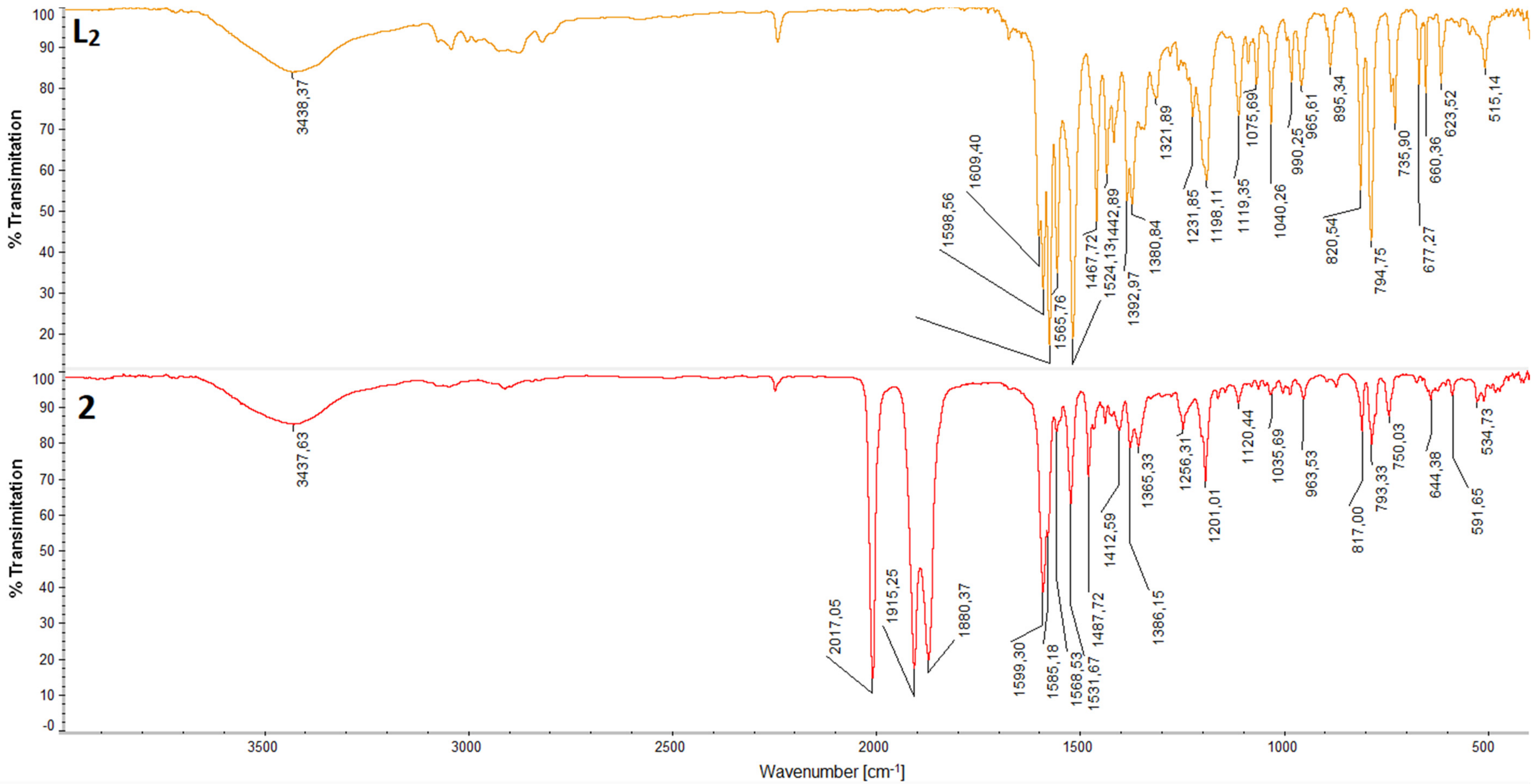

Figure S3. FT-IR spectrum of **2** along with FT-IR spectrum of the free ligand.

NMR spectroscopy

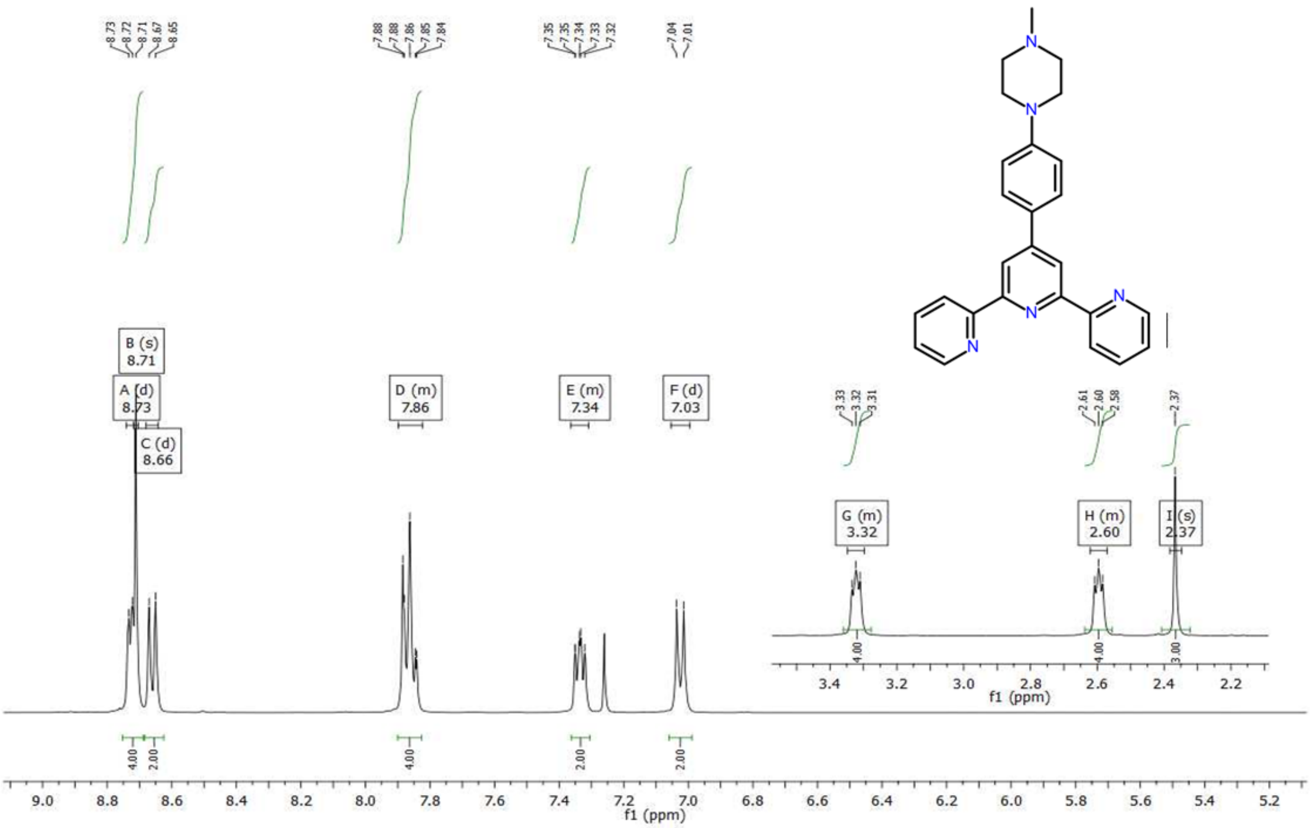

Figure S4. ^{1}H NMR spectrum of L_1.

^{1}H NMR (400 MHz, $CDCl_3$) δ 8.73 (d, J = 4.8 Hz, 2H), 8.71 (s, 2H), 8.66 (d, J = 7.9 Hz, 2H), 7.90 – 7.82 (m, 4H), 7.36 – 7.31 (m, 2H), 7.03 (d, J = 8.8 Hz, 2H), 3.35 – 3.30 (m, 4H), 2.62 – 2.57 (m, 4H), 2.37 (s, 3H).

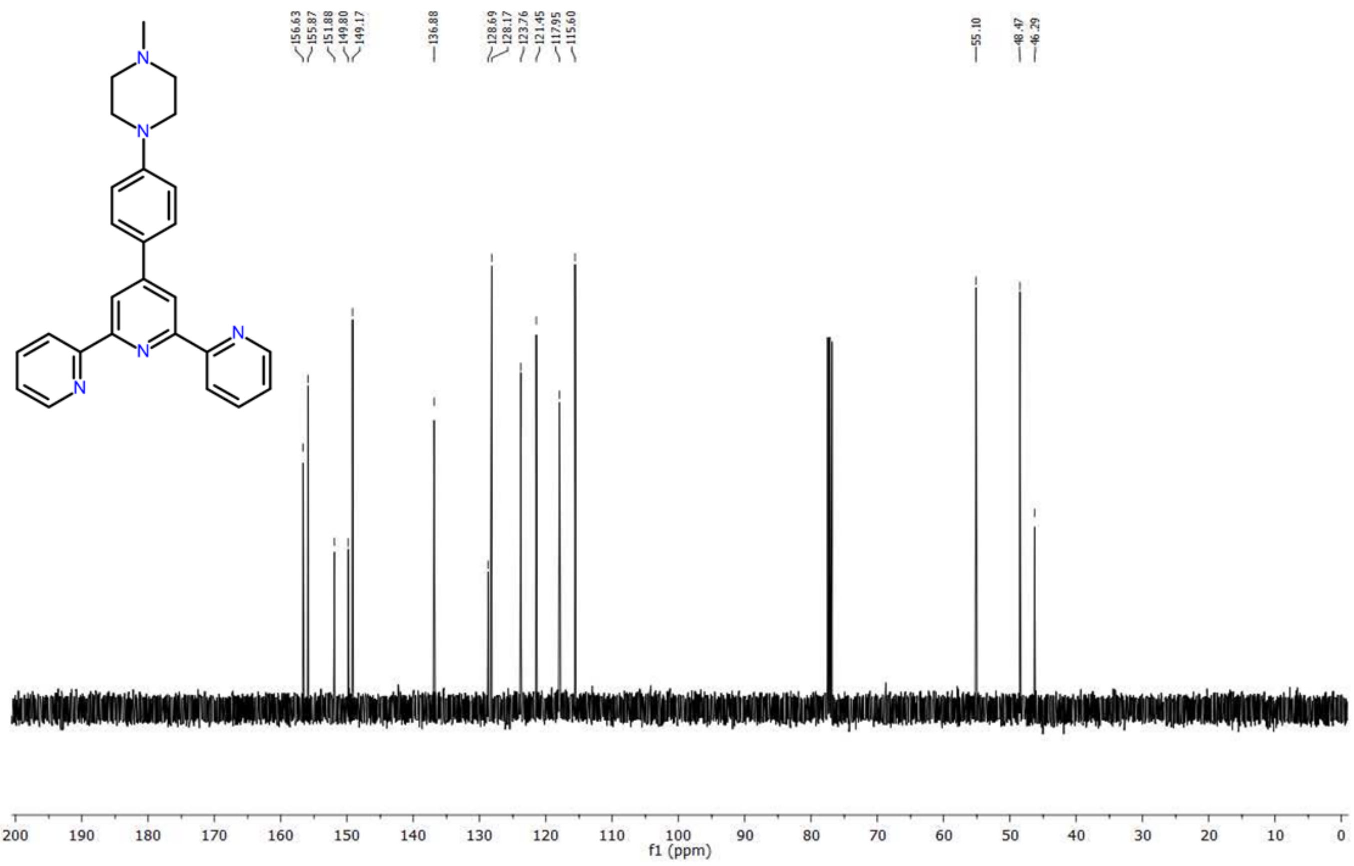

Figure S5. ^{13}C NMR spectrum of L_1.

^{13}C NMR (100 MHz, CDCl$_3$) δ 156.63, 155.87, 151.88, 149.80, 149.17, 136.88, 128.69, 128.17, 123.76, 121.45, 117.95, 115.60, 55.10, 48.47, 46.29.

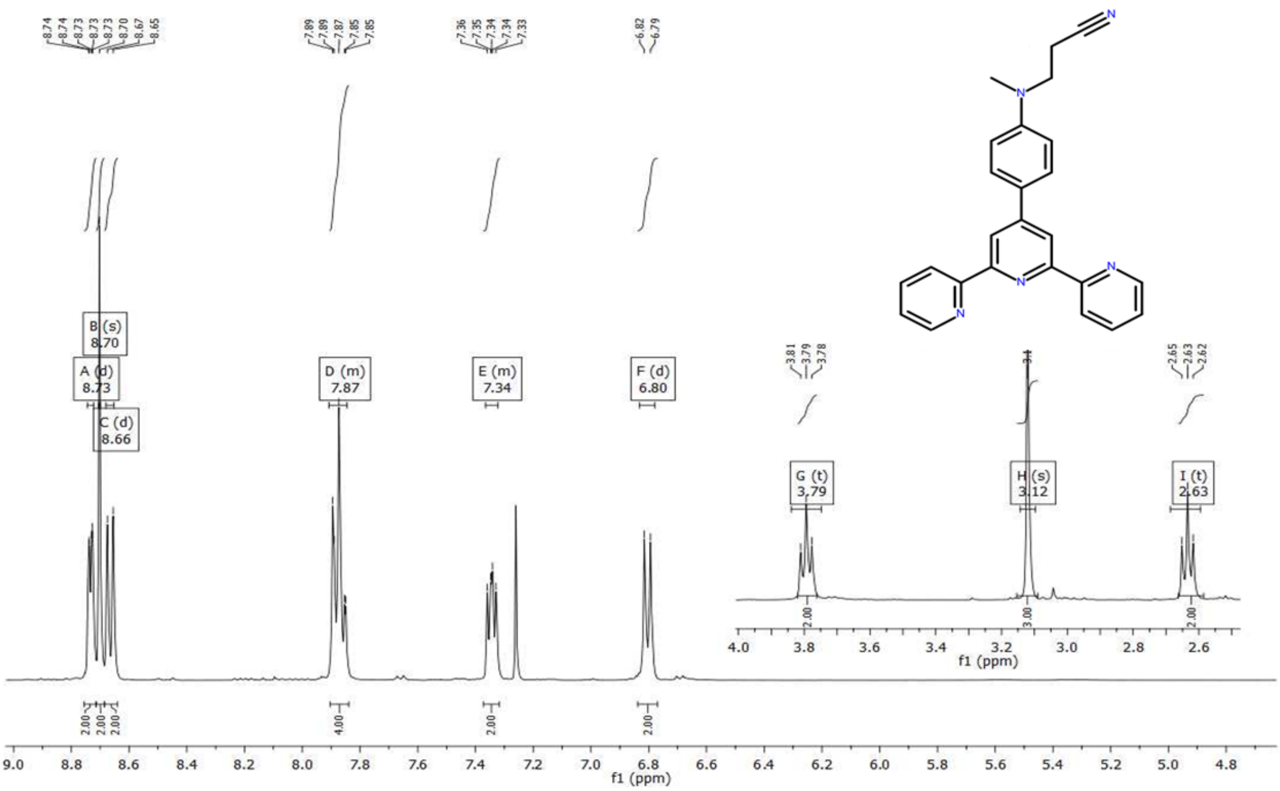

Figure S6. ^{1}H NMR spectrum of L_2.

^{1}H NMR (400 MHz, $CDCl_3$) δ 8.73 (d, J = 5.5 Hz, 2H), 8.70 (s, 2H), 8.66 (d, J = 8.0 Hz, 2H), 7.91 – 7.84 (m, 4H), 7.37 – 7.32 (m, 2H), 6.80 (d, J = 8.4 Hz, 2H), 3.79 (t, J = 6.8 Hz, 2H), 3.12 (s, 3H), 2.63 (t, J = 6.8 Hz, 2H). **^{13}C NMR** (100 MHz, $CDCl_3$) δ 156.57, 155.86, 149.66, 149.15, 148.33, 136.90, 128.52, 127.07, 123.78, 121.43, 118.35, 117.72, 112.49, 48.79, 38.81, 15.49.

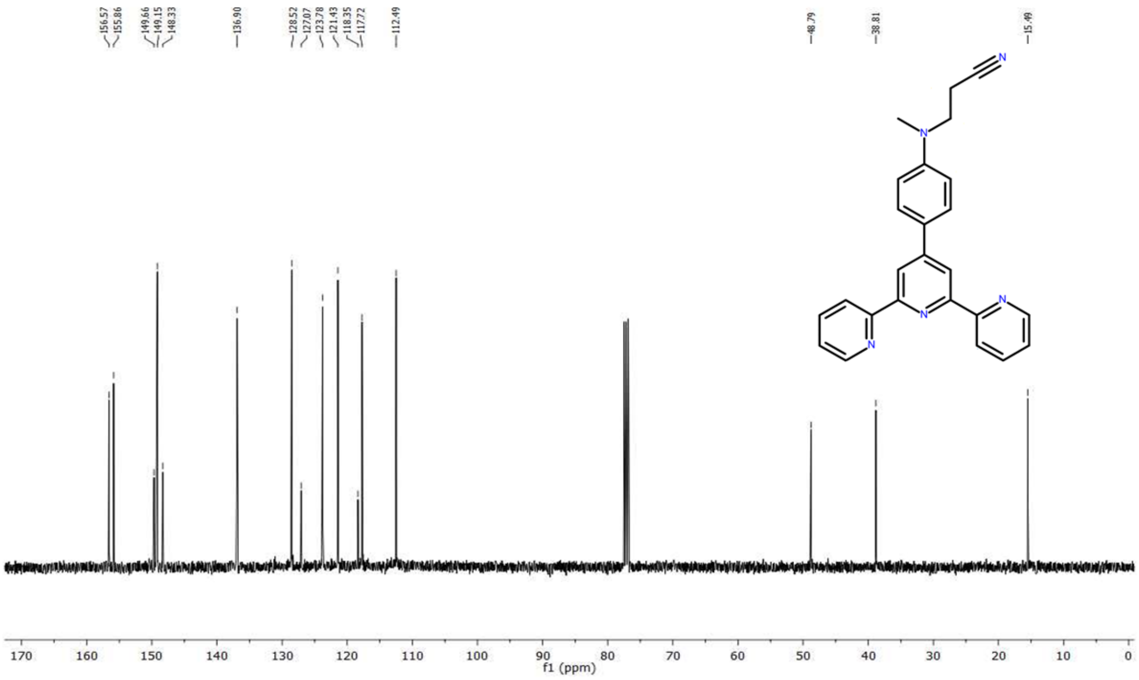

Figure S7. ^{13}C NMR spectrum of L_2.

^{13}C NMR (100 MHz, $CDCl_3$) δ 156.57, 155.86, 149.66, 149.15, 148.33, 136.90, 128.52, 127.07, 123.78, 121.43, 118.35, 117.72, 112.49, 48.79, 38.81, 15.49.

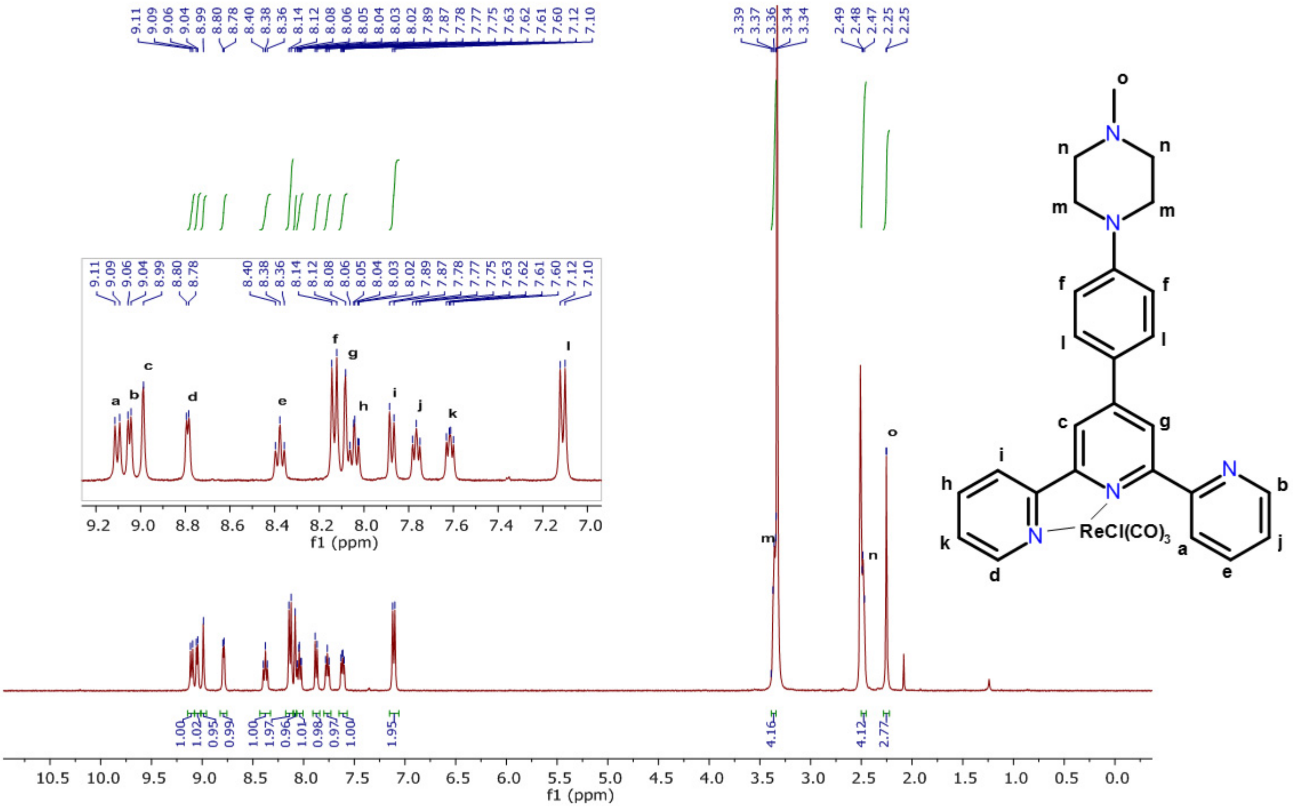

Figure S8. ^{1}H NMR spectrum of **1**.

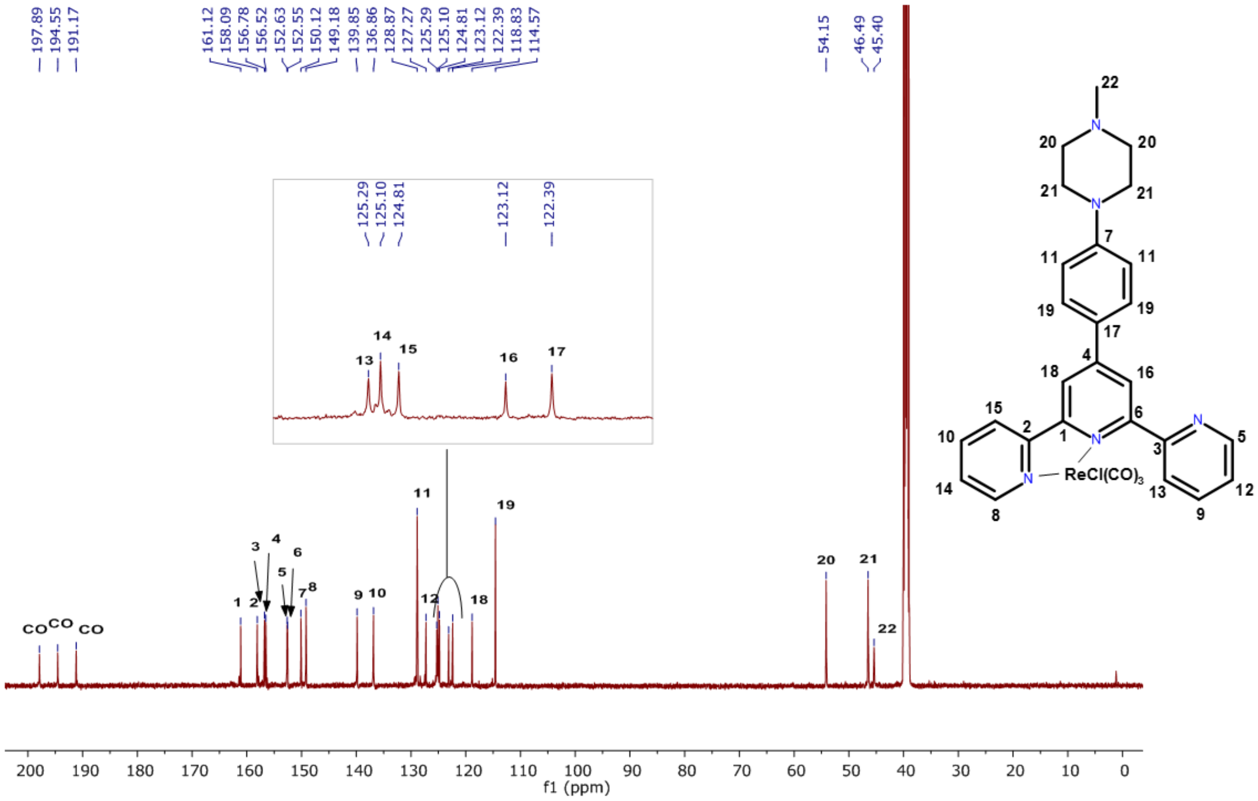

Figure S9. ^{13}C NMR spectrum of **1**.

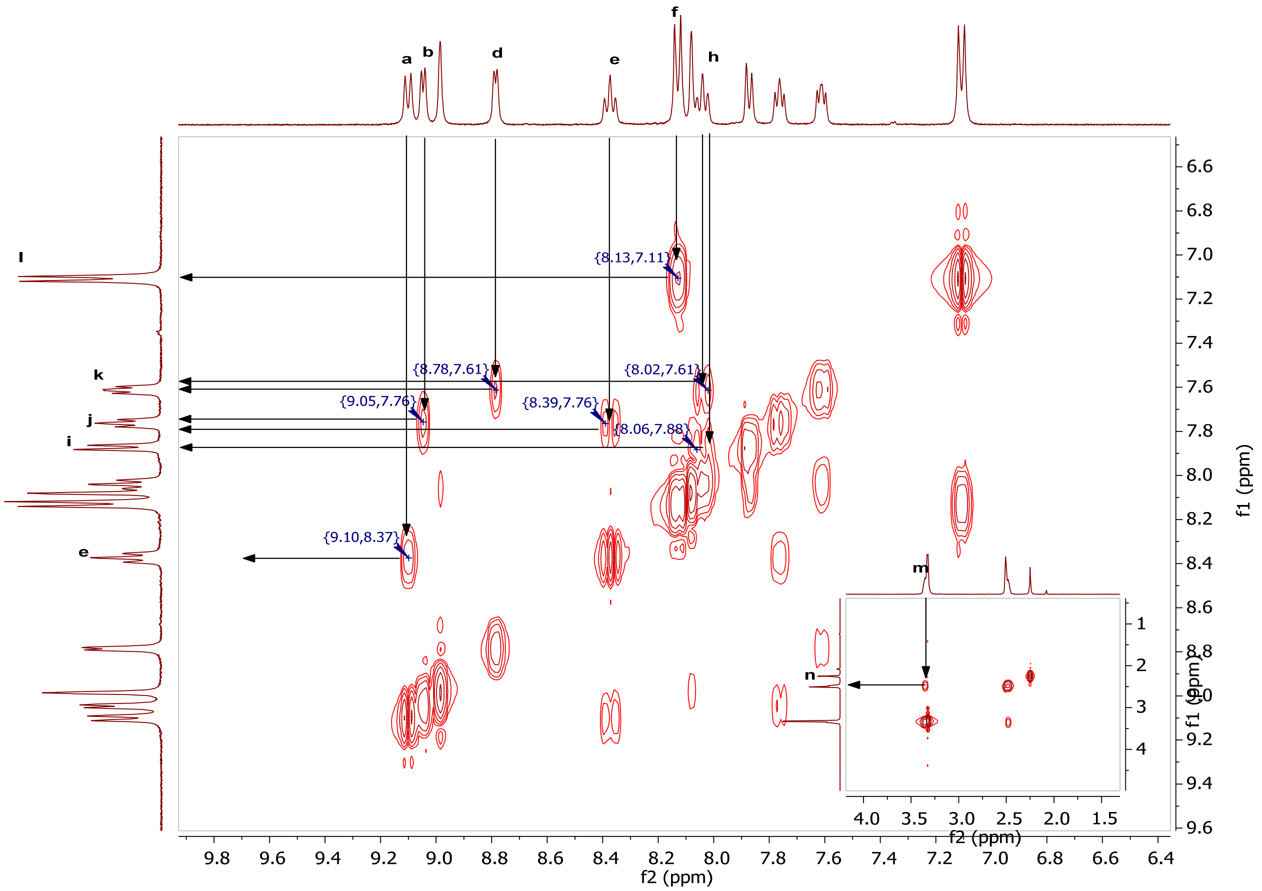

Figure S10. ^{1}H-^{1}H COSY NMR spectrum of **1.**

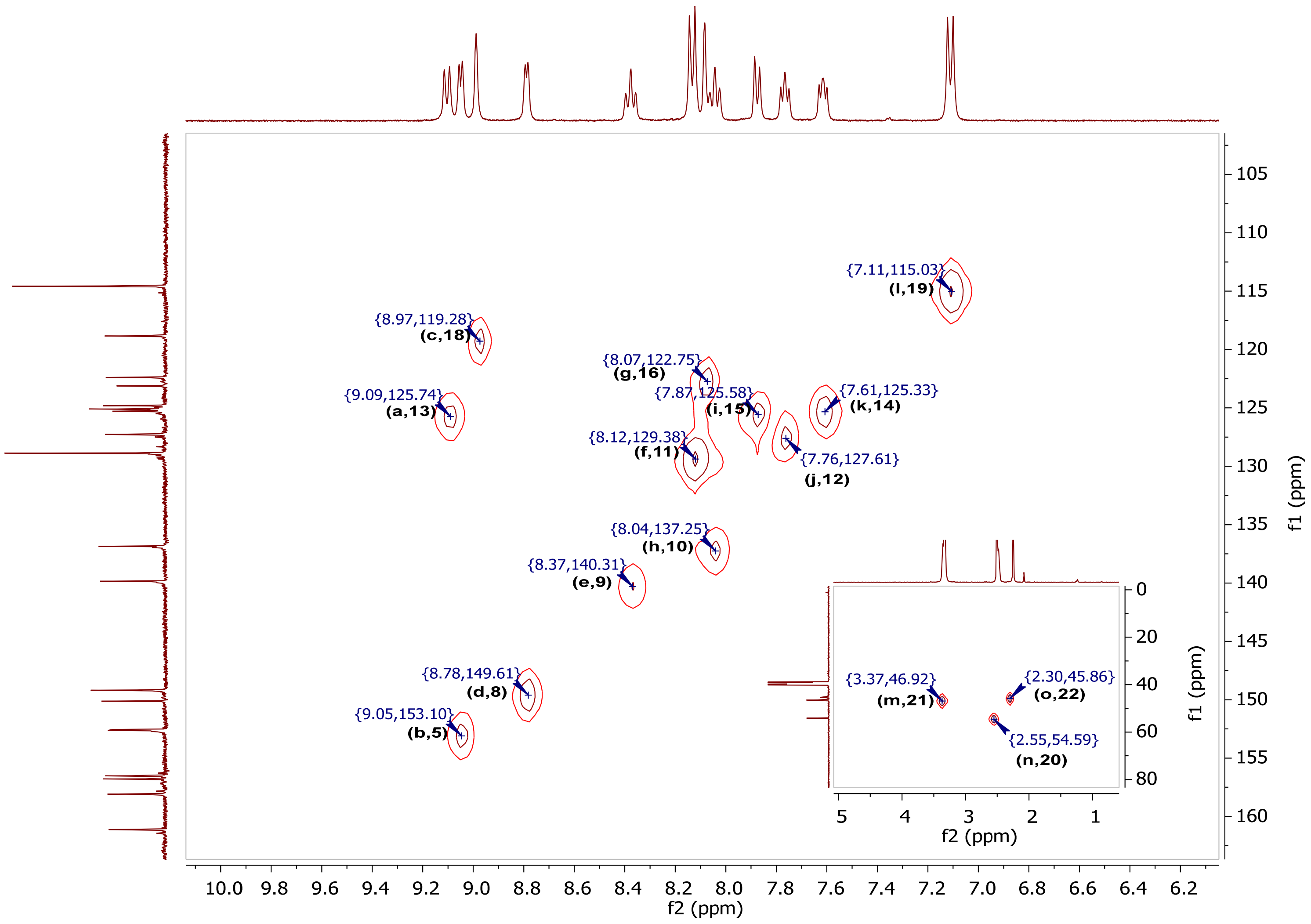

Figure S11. ^{1}H-^{13}C HMQC NMR spectrum of **1**.

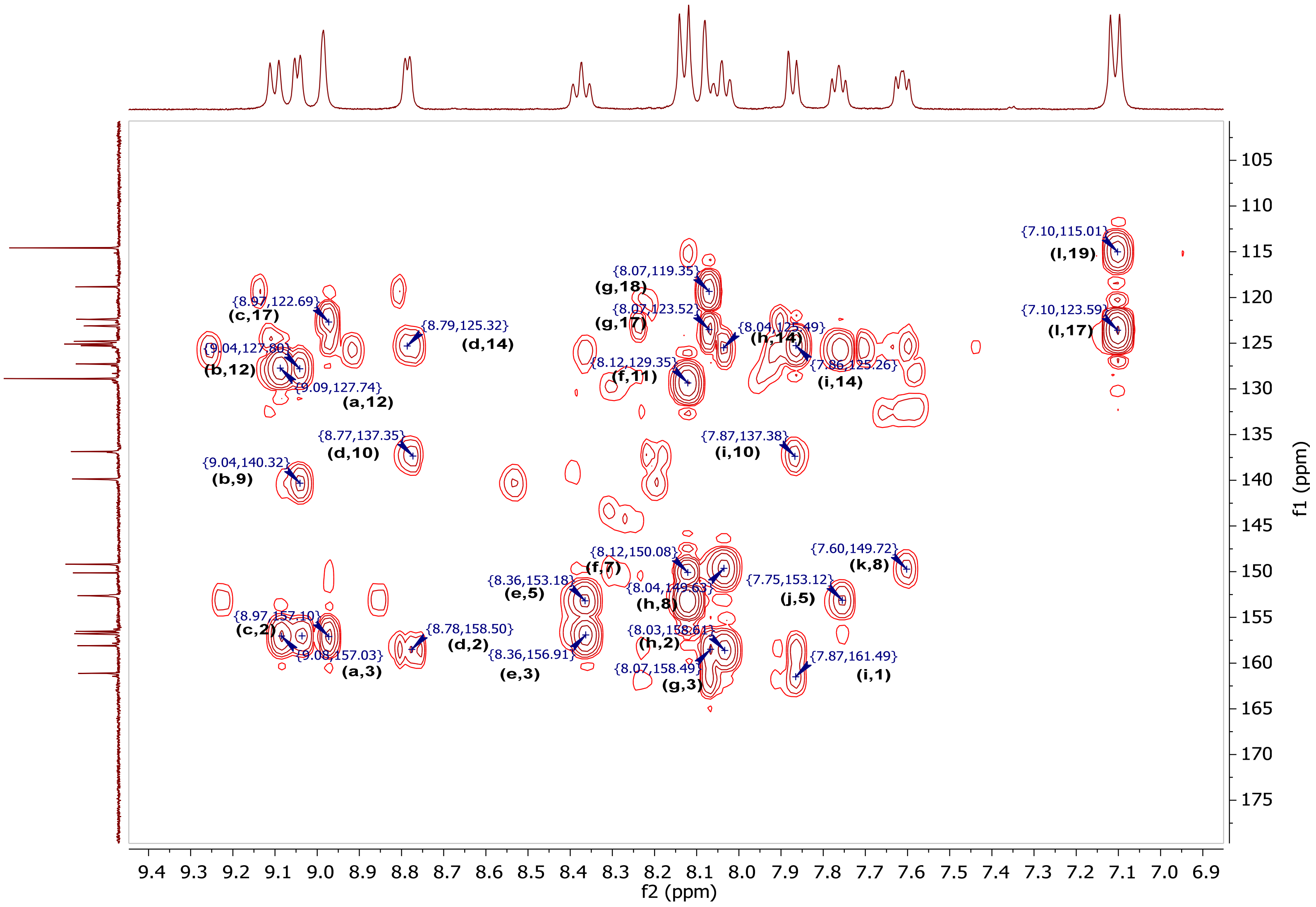

Figure S12. ^{1}H-^{13}C HMBC NMR spectrum of **1.**

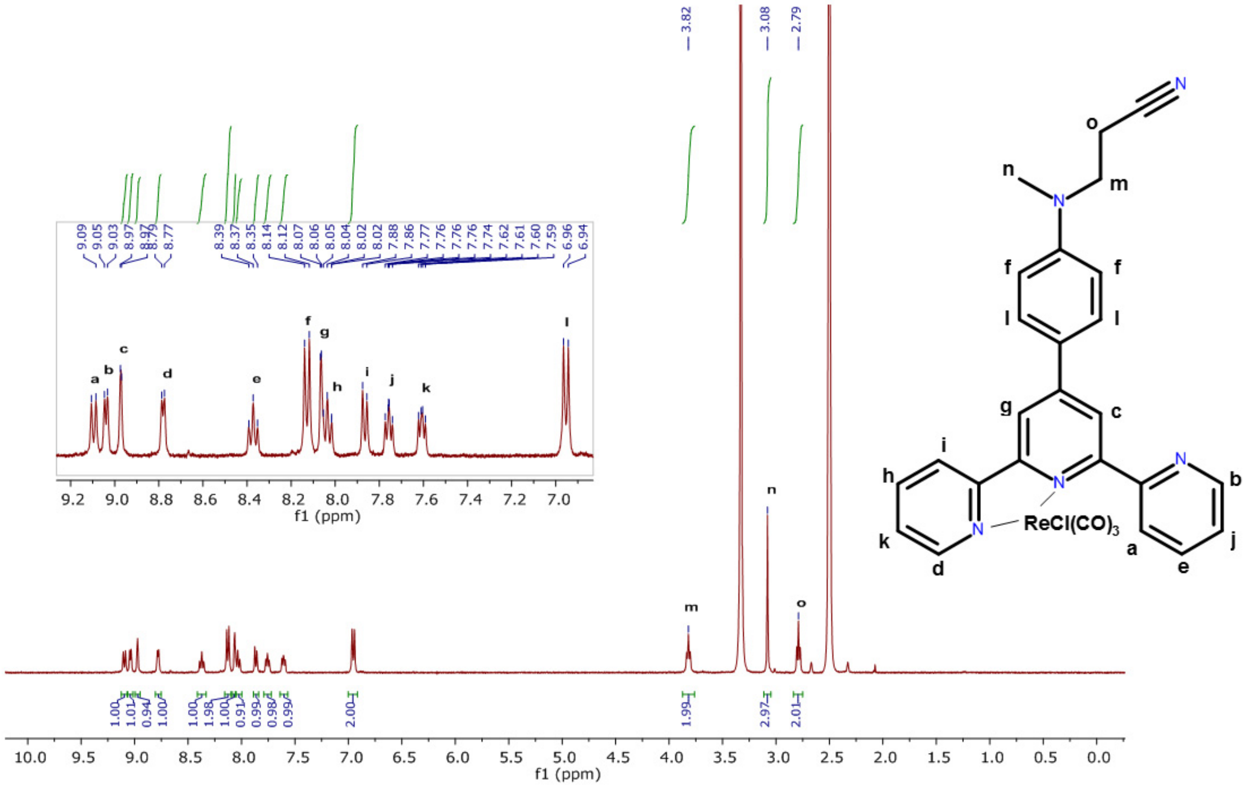

Figure S13. ^{1}H NMR spectrum of **2.**

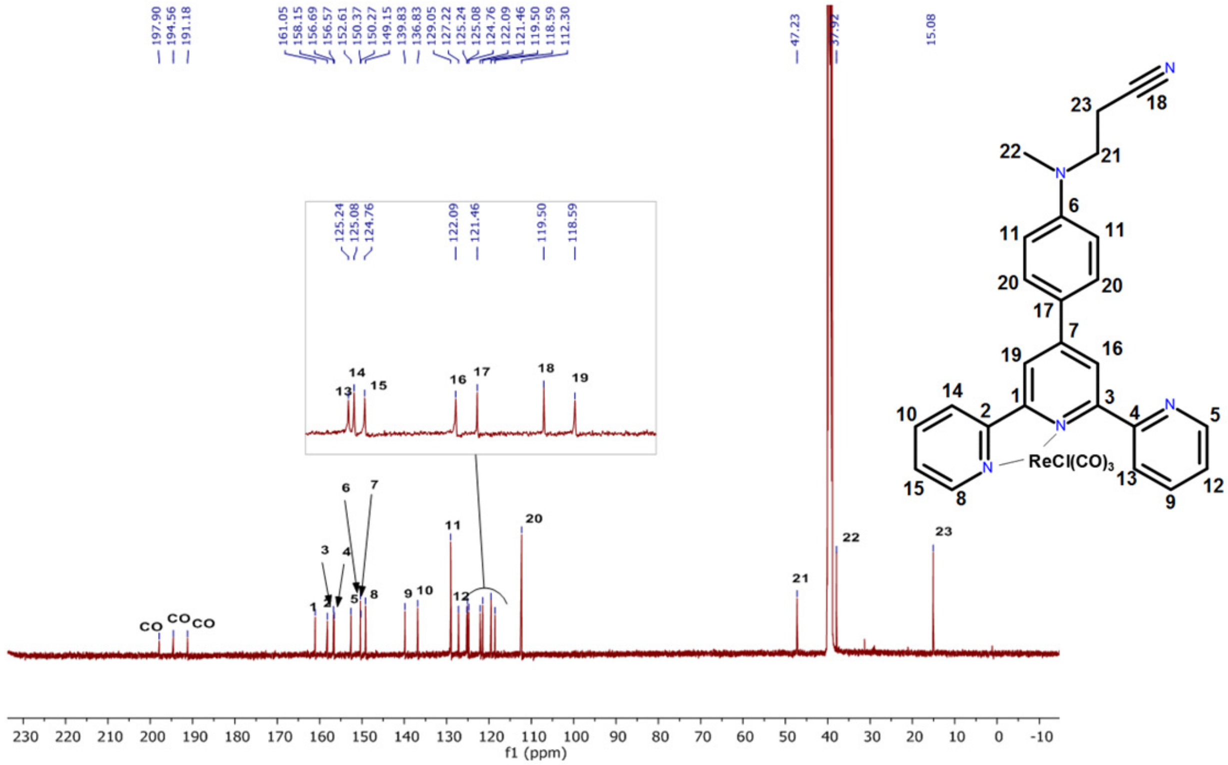

Figure S14. ^{13}C NMR spectrum of **2.**

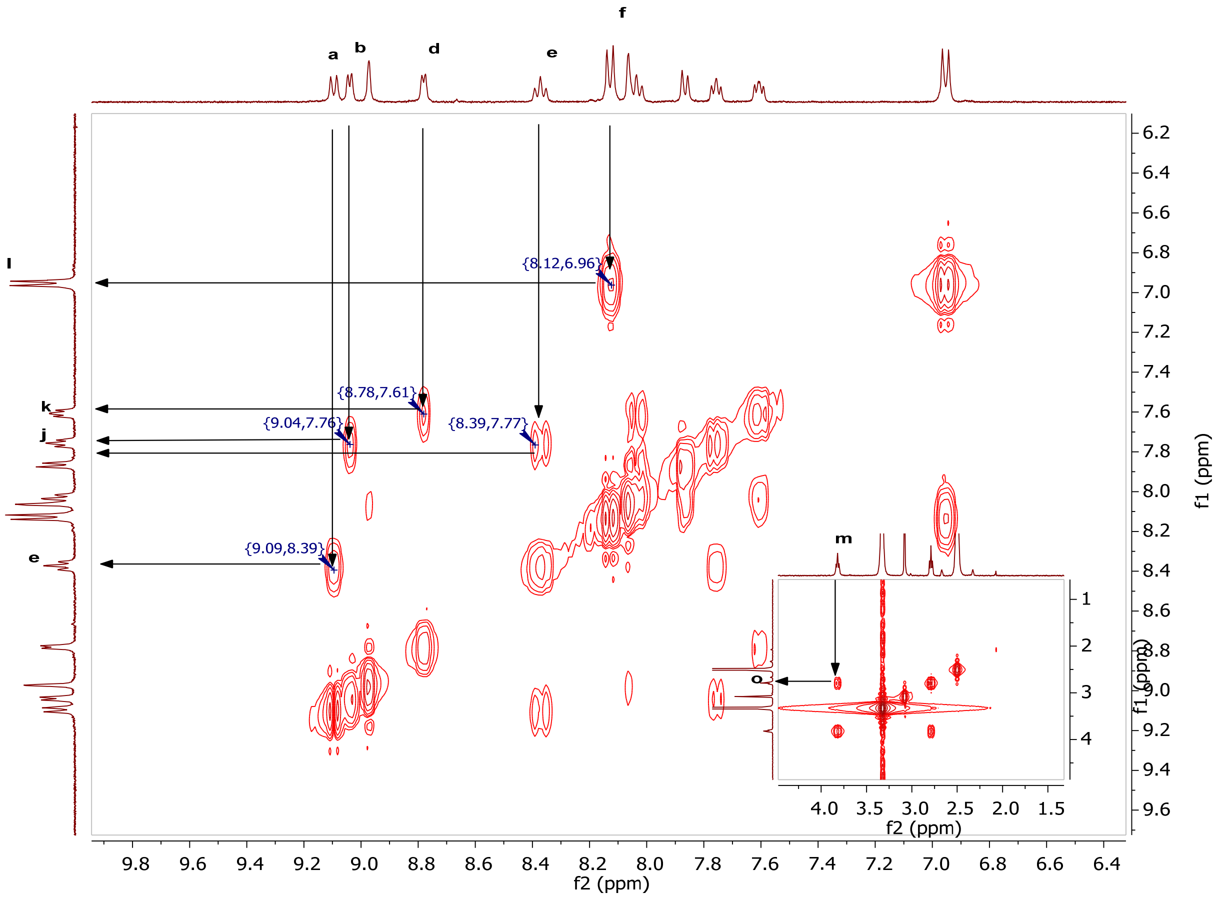

Figure S15. ^{1}H-^{1}H COSY NMR spectrum of **2.**

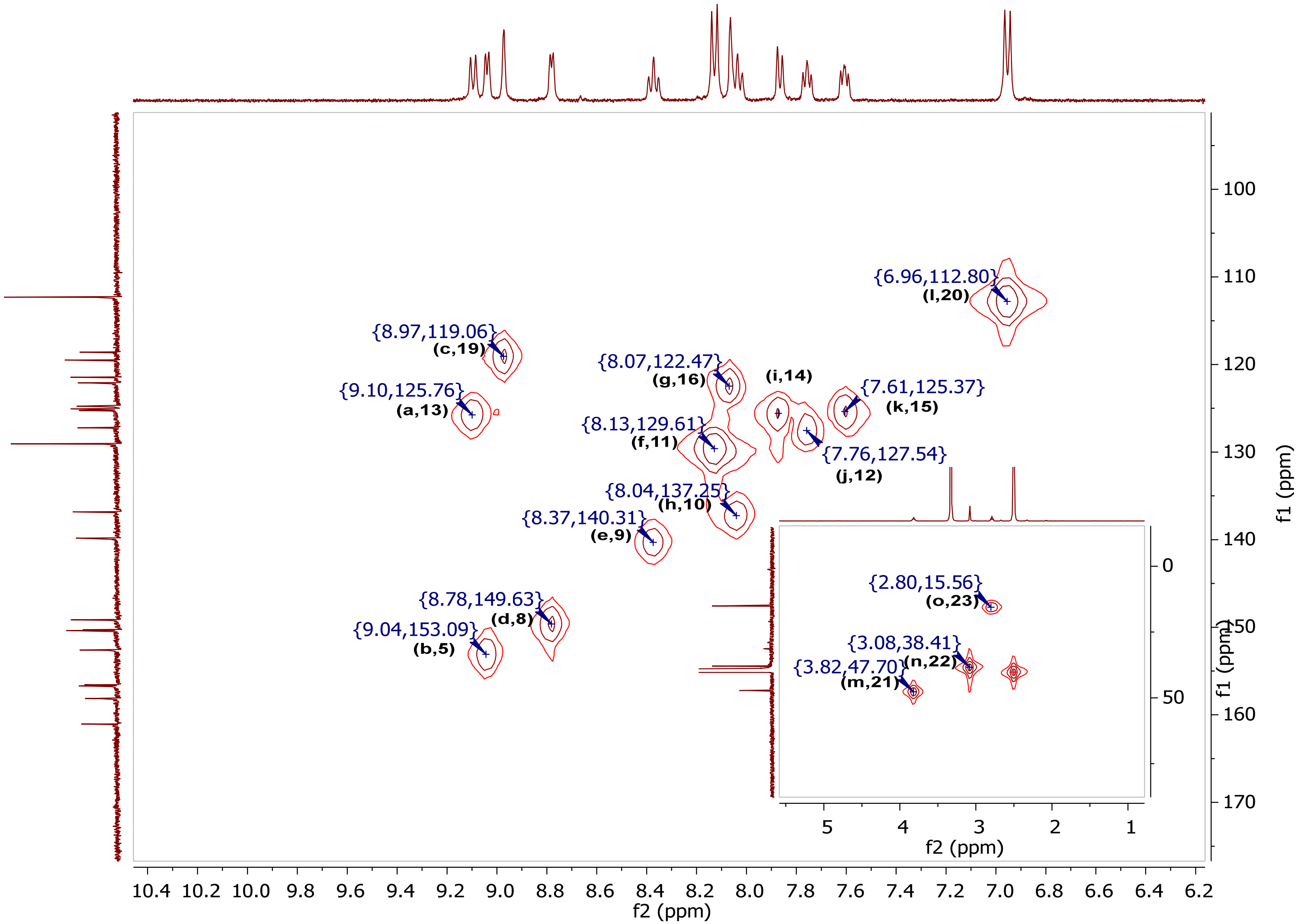

Figure S16. ^{1}H-^{13}C HMQC NMR spectrum of **2**.

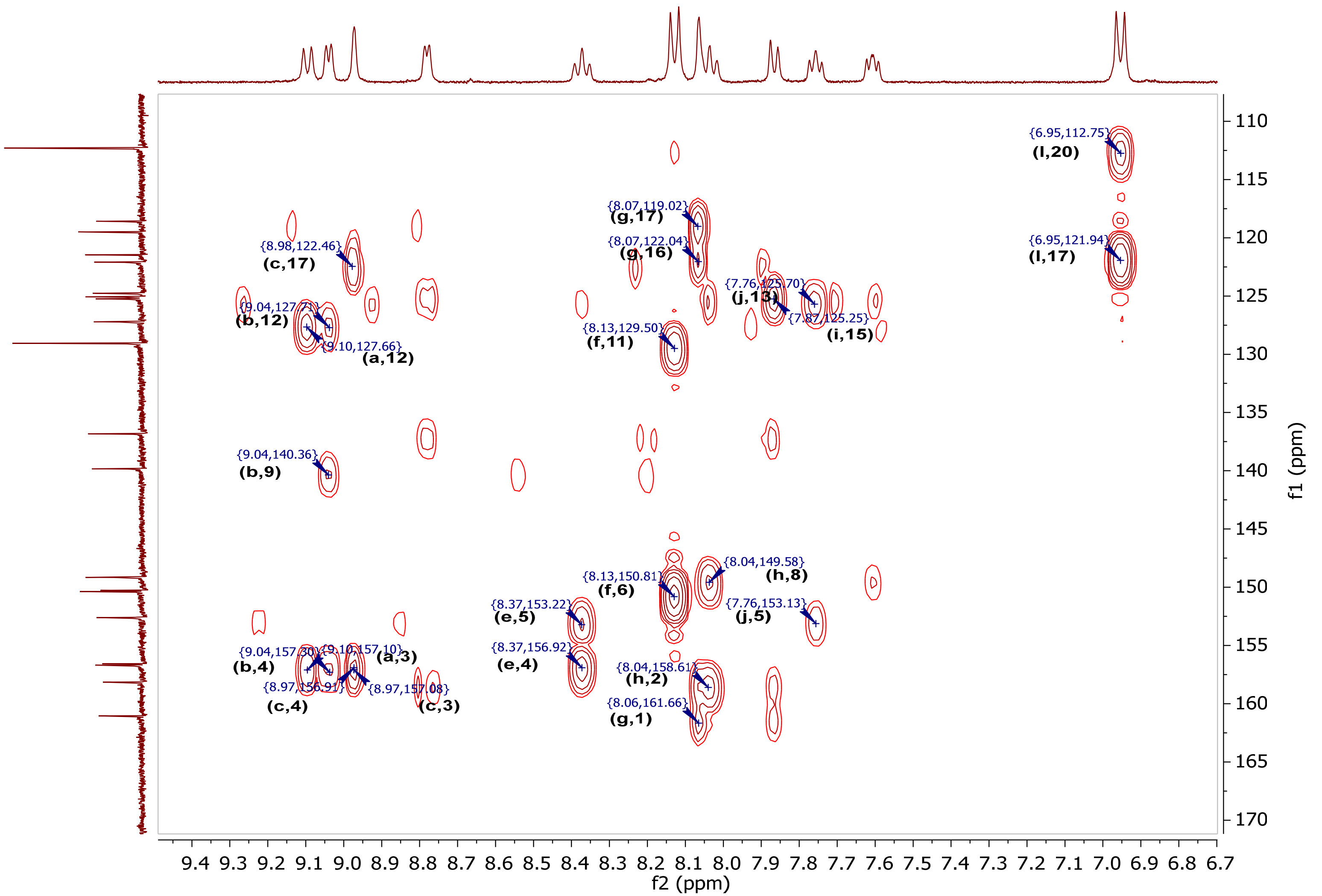

Figure S17. ^{1}H-^{13}C HMBC NMR spectrum of **2.**

HMRS spectroscopy

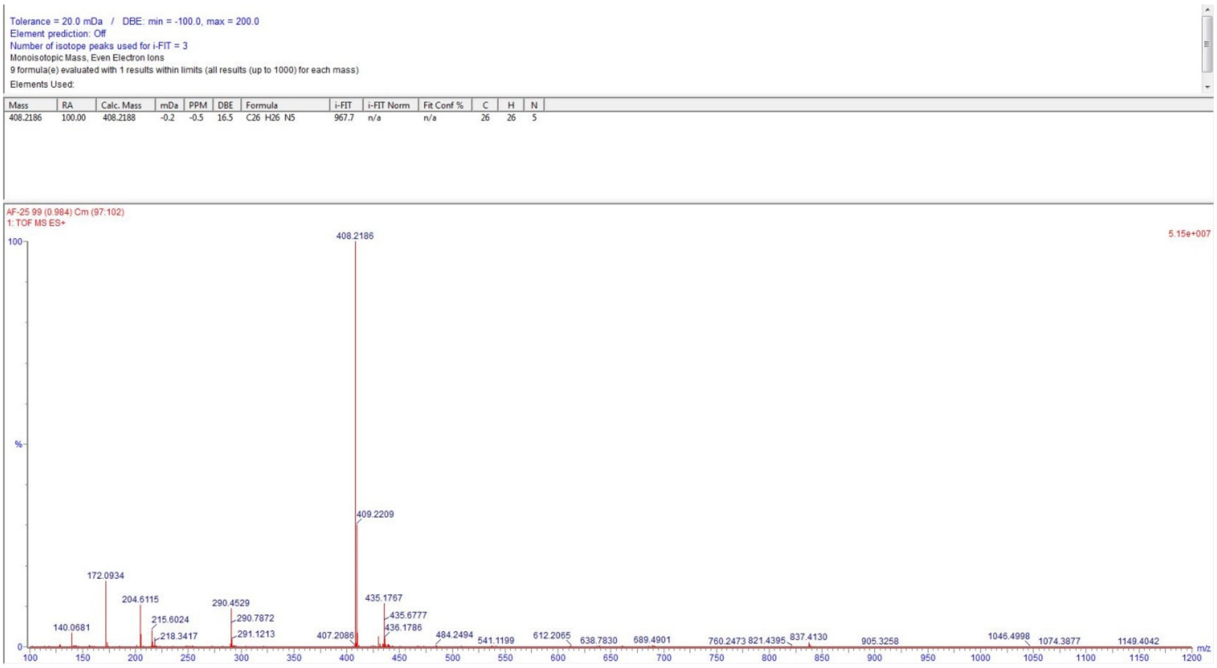

Figure S18. HMRS spectrum of **L_1.**

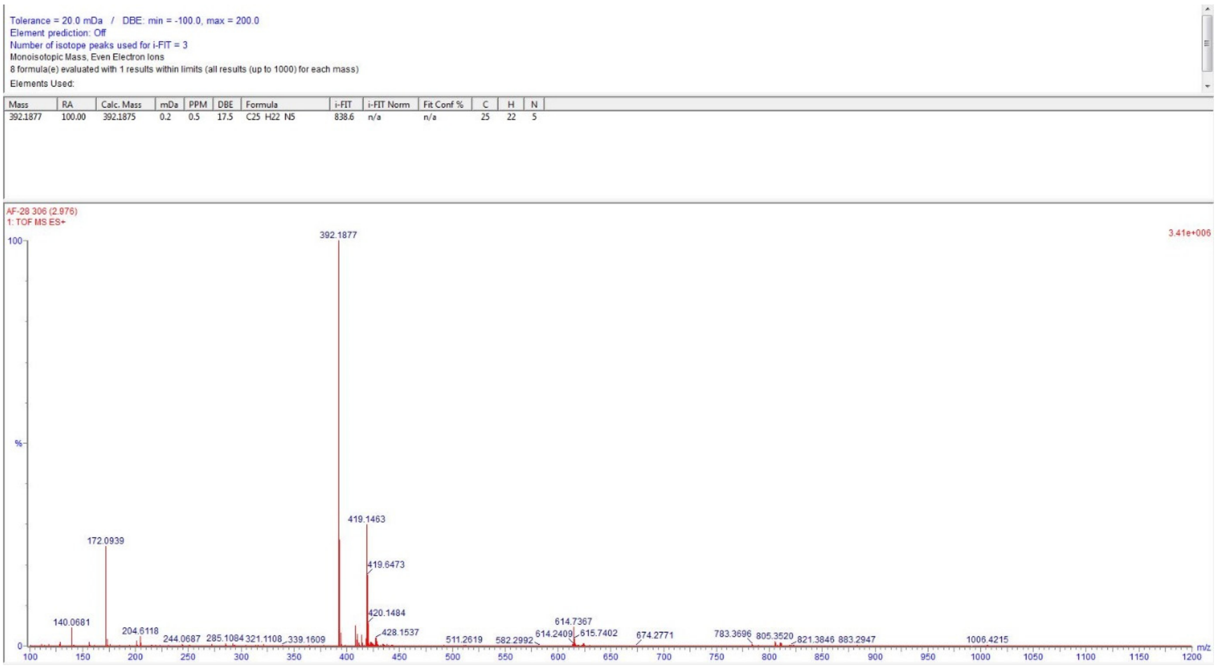

Figure S19. HMRS spectrum of **L_2.**

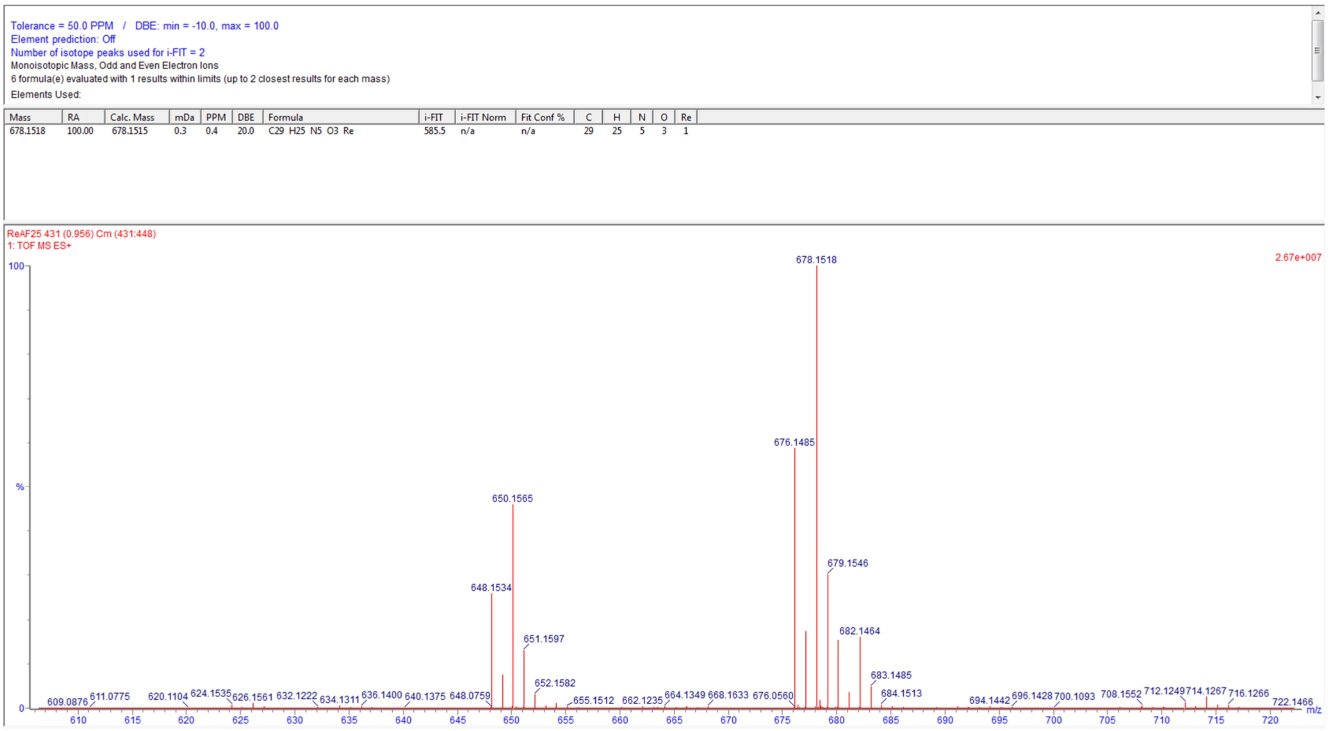

Figure S20. HMRS spectrum of **1.**

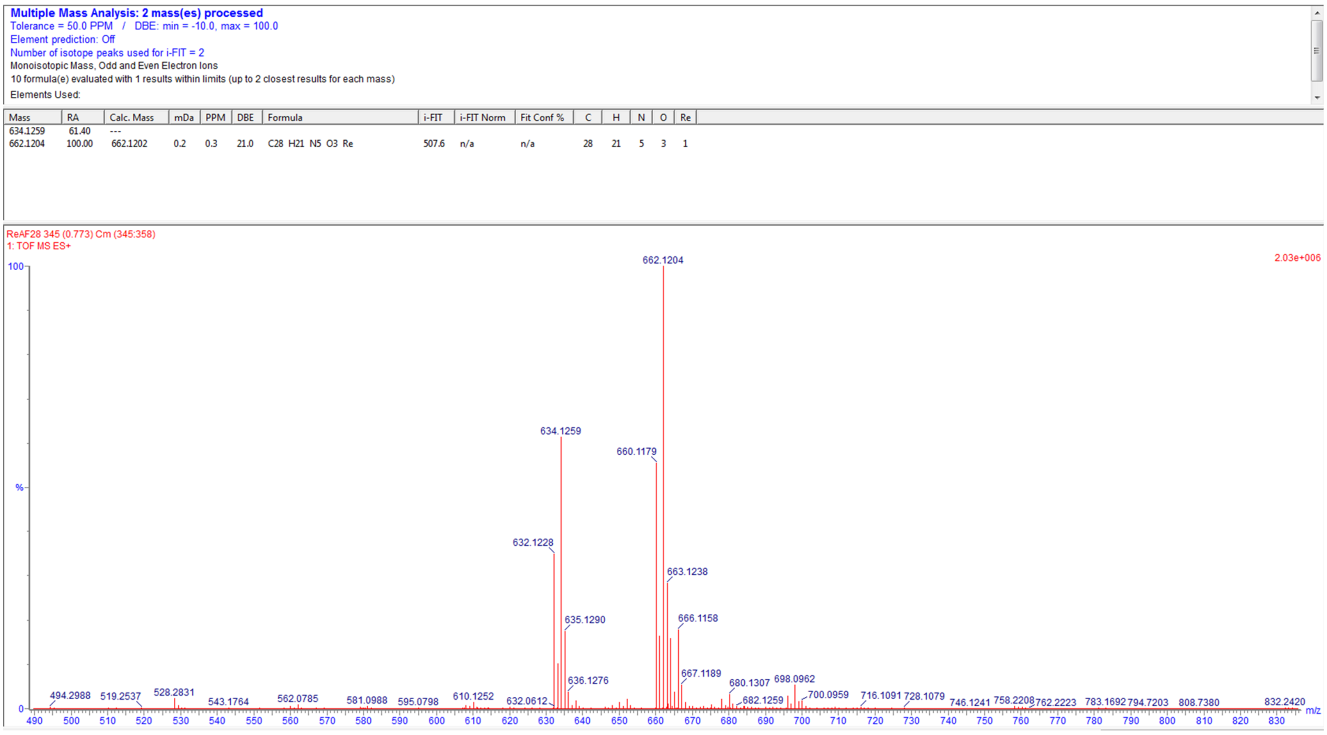

Figure S21. HMRS spectrum of **2.**

UV-VIS studies

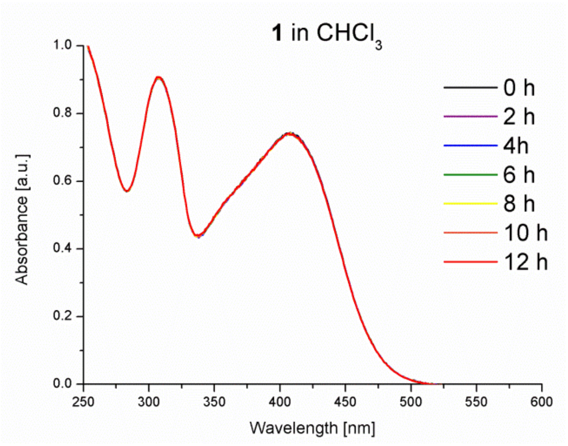

(a)

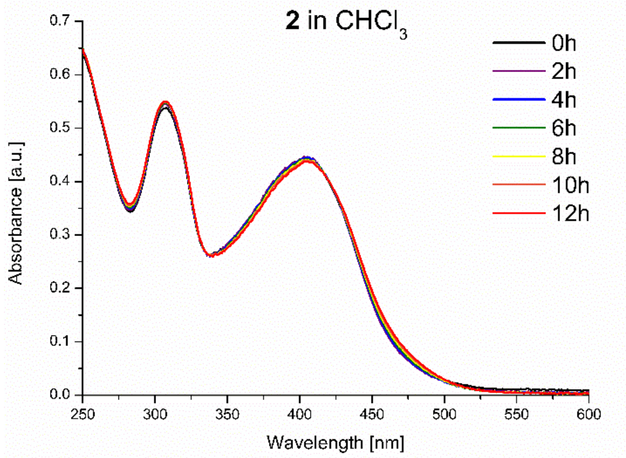

(b)

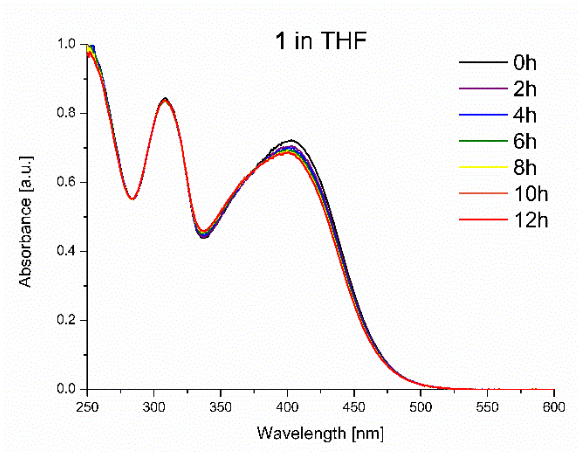

(c)

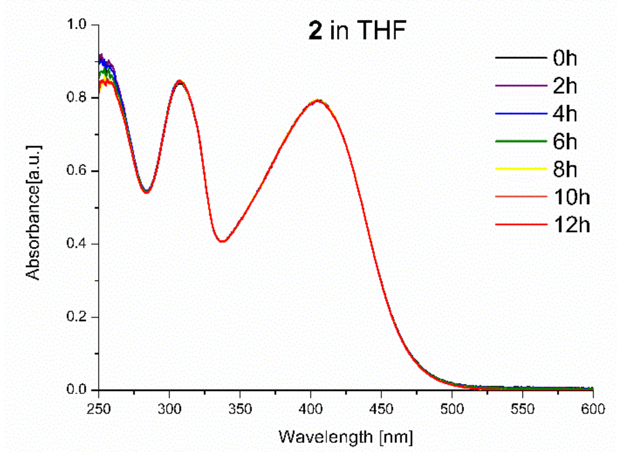

(d)

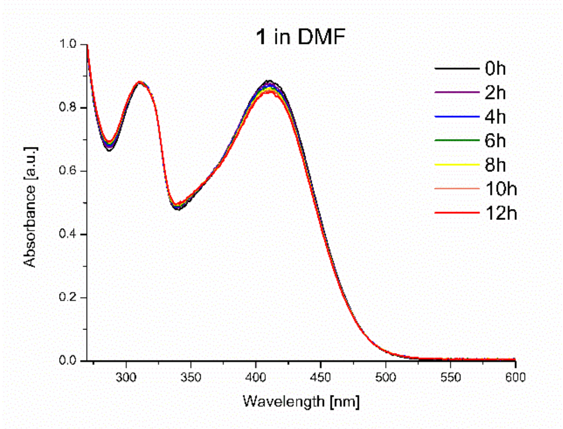

(e)

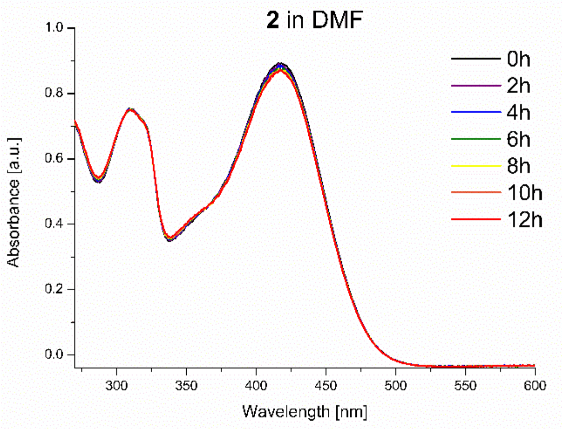

(f)

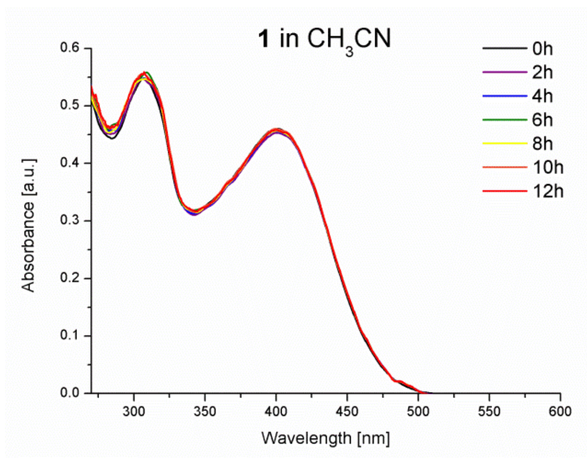	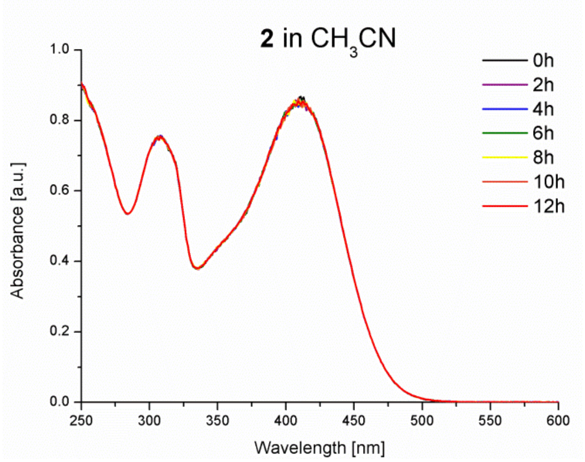
**(g)**	**(h)**

Figure S22. UV-Vis spectra of **1** and **2** in chloroform (a, b), tetrahydrofurane (c, d), N,N–dimethylformamide (e, f) and acetonitrile (g, h), recorded once every two hours over 12h at room temperature.

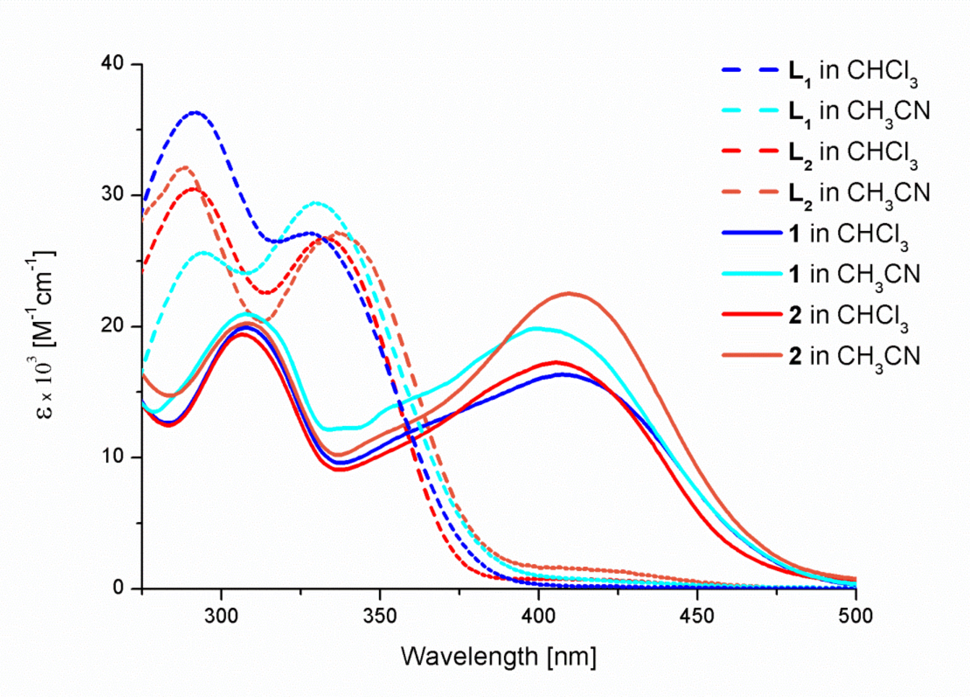

Figure S23. UV−Vis spectra of **1** and **2** in comparison to those for free ligands.

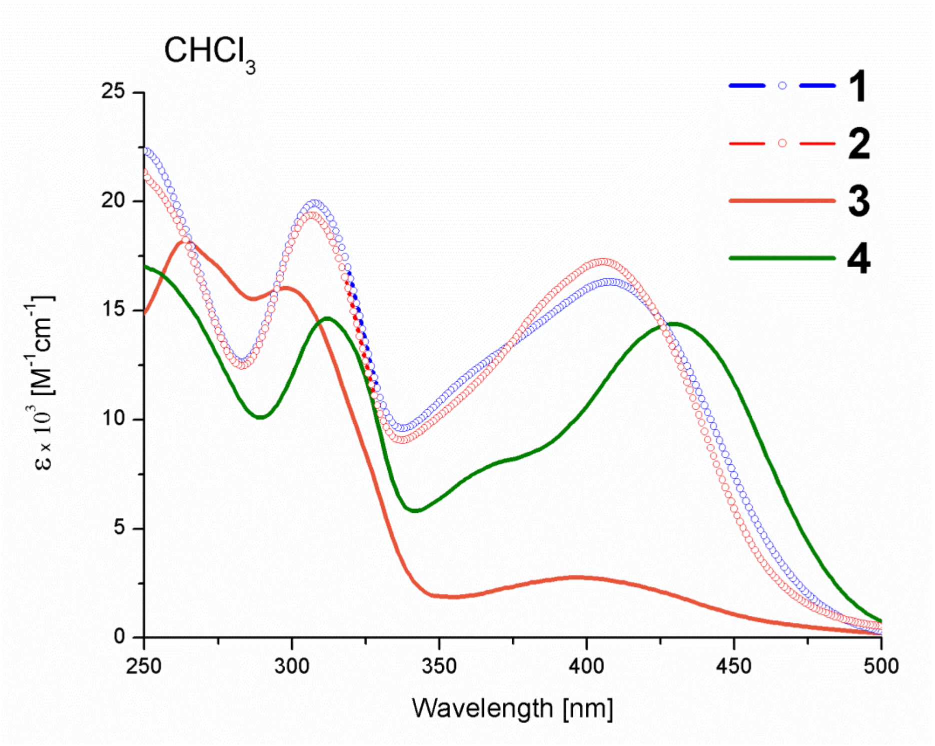

(a)

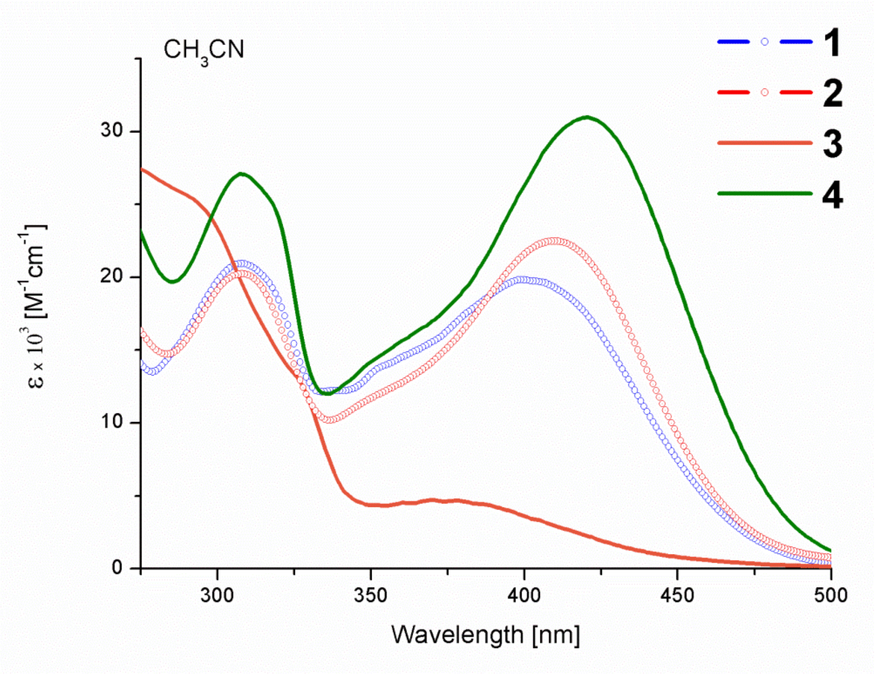

(b)

Figure S24. UV−Vis spectra of **1** and **2** in comparison to those for [ReCl(CO)$_3$(C$_6$H$_5$-terpy-κ^{2}N)] (**3**) and [ReCl(CO)$_3$(Me$_2$N-C$_6$H$_4$-terpy-κ^{2}N)] (**4**) in CHCl$_3$ (a) and CH$_3$CN (b).

Table S6. The absorption maxima and molar extinction coefficient for **1** and **2** with spectral data for [ReCl(CO)$_3$(C$_6$H$_5$-terpy-κ^{2}N)] (**3**) and [ReCl(CO)$_3$(Me$_2$N-C$_6$H$_4$-terpy-κ^{2}N)] (**4**) chloroform (a), tetrahydrofurane (b), N,N–dimethylformamide (c) and acetonitrile (d).

Compound	λ/nm (10^4 ε/dm^3·mol^{-1}·cm^{-1})			
	CHCl$_3$	THF	MeCN	DMF
**1**	405 (1.63)	405 (1.75)	402 (1.93)	409 (1.66)
**2**	405 (1.72)	406 (1.78)	405 (2.24)	417 (2.26)
**3**	398 (0.27)	400 (0.31)	375 (0.46)	385 (0.51)
**4**	430 (1.44)	415 (2.69)	419 (1.65)	425 (2.34)

Absorption properties - TDDFT calculations

Table S7. The energies and characters of spin-allowed electronic transitions assigned to the lowest wavelength absorption bands of complexes **1** and **2** computed at the DFT/PBE1PBE/def2-TZVPD/def2–TZVP level.

Compound (medium)	Experimental absorption λ; nm (10^4 ε; M^{-1}cm^{-1})	Calculated transitions				
		Major contribution (%)	Predominant character	E [eV]	λ [nm]	Oscillator strength
**1** (CHCl$_3$)	405 (1.89)	H–1 → LUMO (55%)<br>HOMO → LUMO (39%)	MLCT<br>ILCT/IL	2.78	446.6	0.1234 S_1
		H–1 → LUMO (44%)<br>HOMO → LUMO (45%)	MLCT<br>ILCT/IL	2.88	430.8	0.3347 S_2
		H–2 → LUMO (83%)<br>HOMO → LUMO (15%)	MLCT<br>ILCT/IL	3.09	401.0	0.0496 S_3
		H–4 → LUMO (98%)	MLCT	3.32	373.6	0.0038 S_4
		HOMO → L+1 (95%)	ILCT/IL	3.41	363.9	0.3076 S_5
		H–1→L+1 (96%)	MLCT	3.55	348.9	0.0042 S_6
**1** (CH$_3$CN)	402 (2.87)	HOMO → LUMO (90%)	ILCT/IL	2.83	437.6	0.4426 S_1
		H–1 → LUMO (94%)	MLCT	2.99	414.0	0.0710 S_2
		H–3 → LUMO (84%)<br>H–2 → LUMO (11%)	ILCT/IL<br>MLCT	3.22	384.8	0.0108 S_3
		HOMO → L+1 (96%)	ILCT/IL	3.41	363.3	0.3291 S_4
		H–4 → LUMO (97%)	MLCT	3.44	360.1	0.0045 S_5
**2** (CHCl$_3$)	405 (1.57)	H–1 → LUMO (65%)<br>HOMO → LUMO (30%)	MLCT<br>ILCT/IL	2.78	446.4	0.0649 S_1
		HOMO → LUMO (48%)<br>H–1 → LUMO (34%)<br>H–2 → LUMO (17%)	ILCT/IL<br>MLCT<br>MLCT	2.90	427.8	0.3358 S_2
		H–2 → LUMO (76%)<br>HOMO → LUMO (21%)	MLCT<br>ILCT/IL	3.12	397.4	0.1006 S_3
		H–3 → LUMO (98%)	MLCT	3.31	374.4	0.0039 S_4
		HOMO → L+1 (90%)	ILCT/IL	3.45	359.7	0.2728 S_5
		H–1 → L+1 (93%)	MLCT	3.55	349.7	0.0155 S_6
**2** (CH$_3$CN)	410 (2.69)	HOMO → LUMO (88%)	ILCT/IL	2.86	434.1	0.4142 S_1
		H–1 → LUMO (92%)	MLCT	3.00	413.0	0.0896 S_2
		H–2 → LUMO (94%)	MLCT	3.23	383.6	0.0108 S_3

		HOMO → L+1 (95%)	ILCT/IL	3.43	361.6	0.3188 S_4
		H–3 → LUMO (96%)	MLCT	3.45	359.8	0.0084 S_5

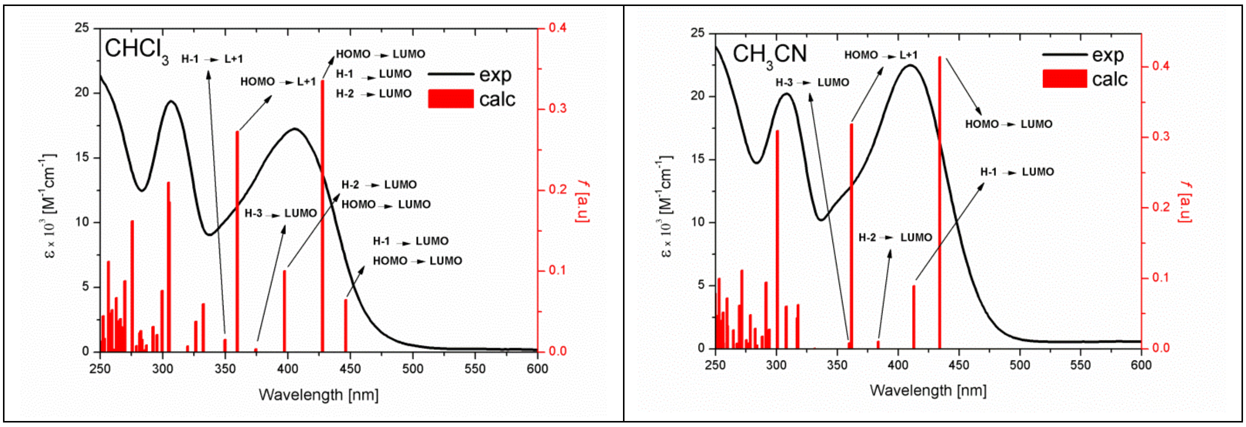

Figure S25. Experimental (black line) absorption spectra of **2** alongside red vertical lines presenting singlet-singlet transitions with corresponding oscillator strengths, computed at TD-DFT/PCM/PBE1PBE/def2-TZVPD/def2-TZVP level with the use of the PCM model at polarities corresponding to MeCN and $CHCl_3$.

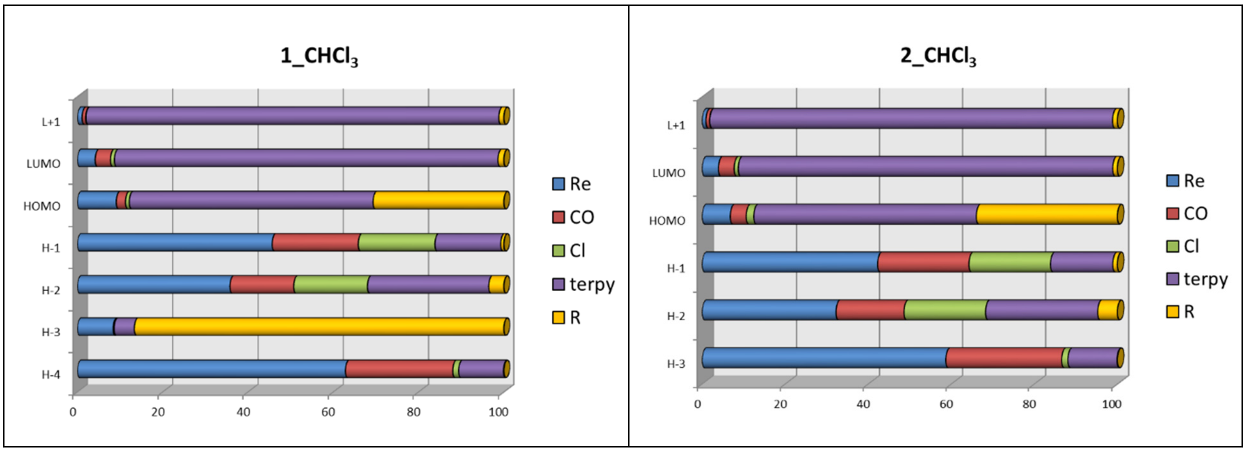

Figure S26. Percentage composition of molecular orbitals for **1** and **2** (in $CHCl_3$) computed at the DFT/PBE1PBE/def2-TZVPD/def2–TZVP level.

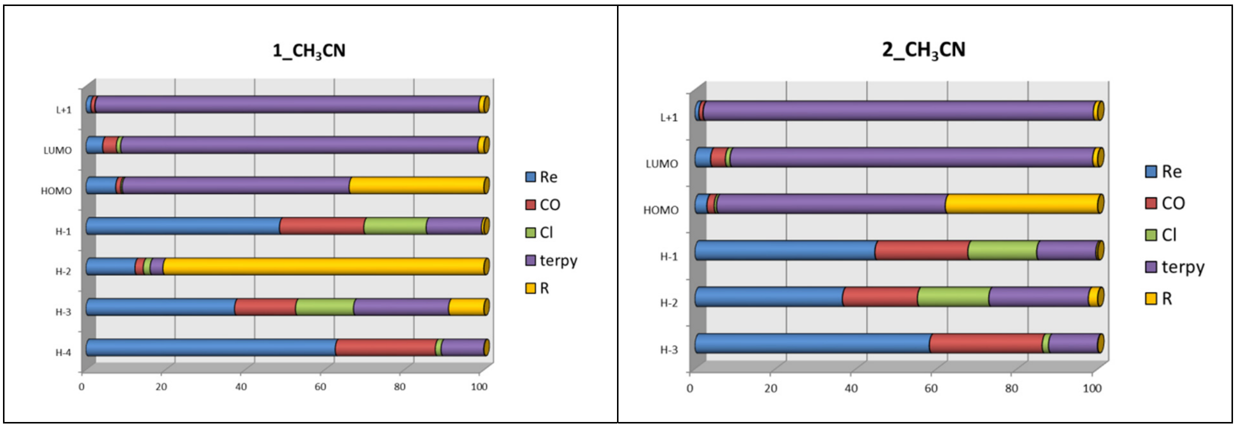

Figure S27. Percentage composition of molecular orbitals for **1** and **2** (in CH_3CN) computed at the DFT/PBE1PBE/def2-TZVPD/def2–TZVP level.

Table S8. Selected molecular orbitals of **1** computed at the DFT/PBE1PBE/def2-TZVPD/def2–TZVP level.

	$CHCl_3$	CH_3CN
H–4	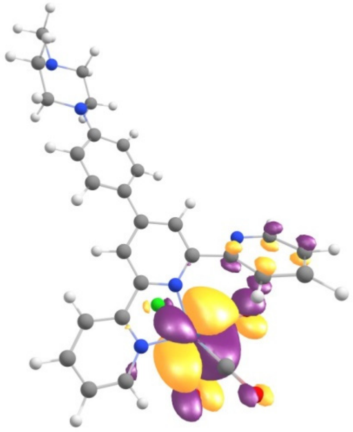	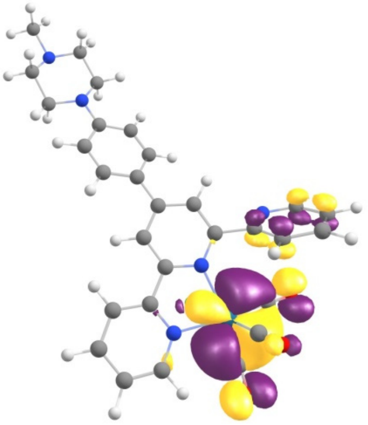
H–3	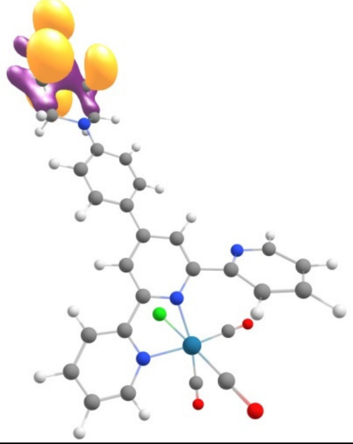	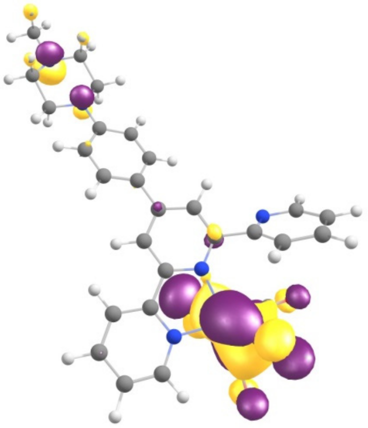

H–2	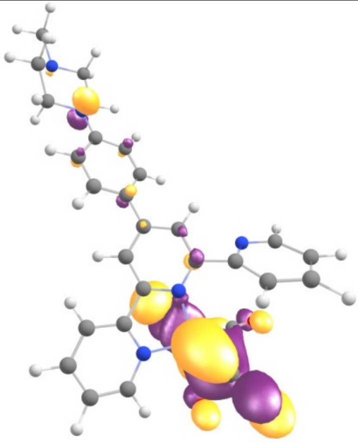	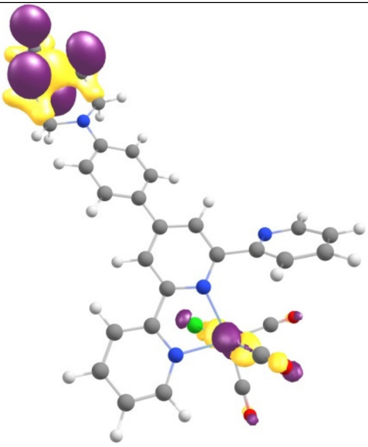
H–1	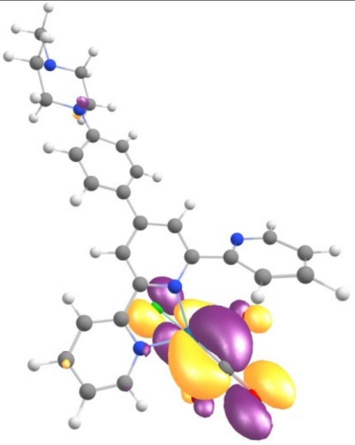	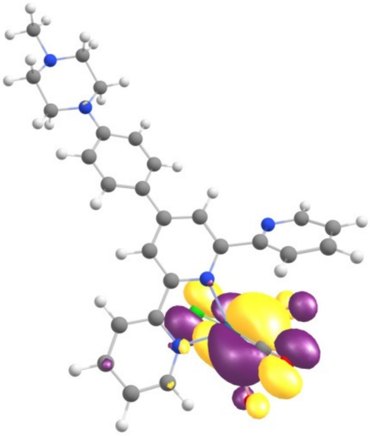
HOMO	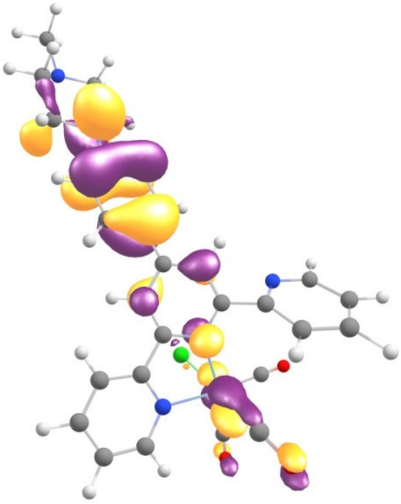	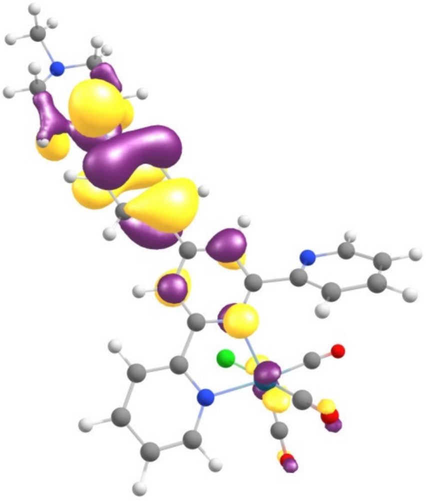

LUMO	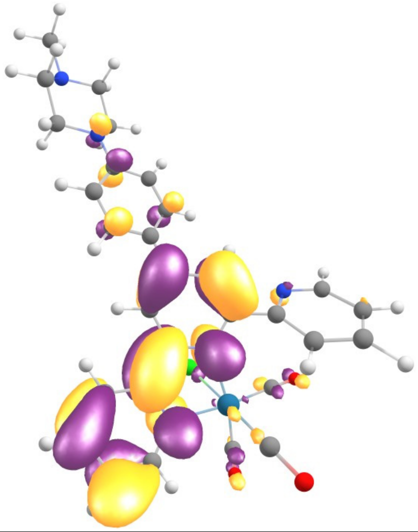	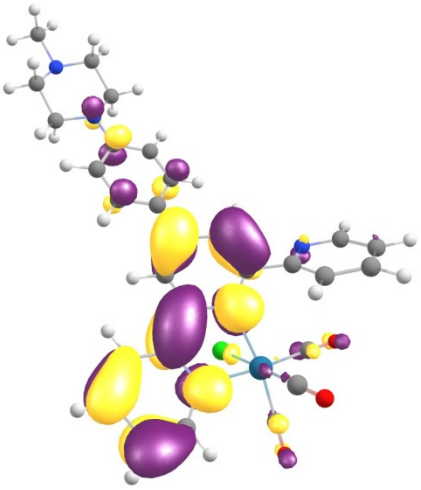
L+1	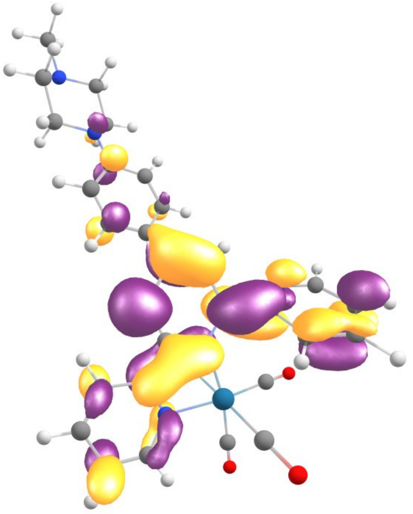	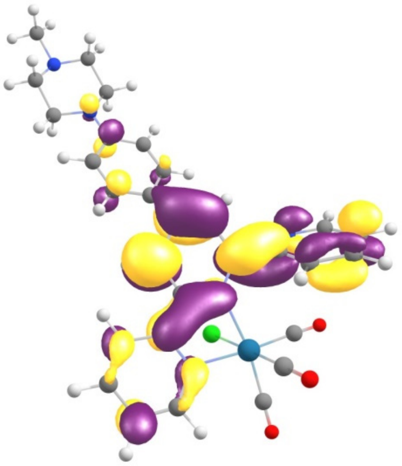

Table S9. Selected molecular orbitals of **2** computed at the DFT/PBE1PBE/def2-TZVPD/def2–TZVP level.

	$CHCl_3$	CH_3CN
H–3	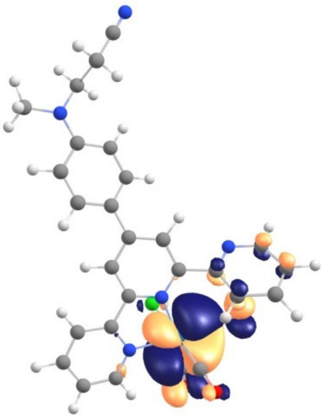	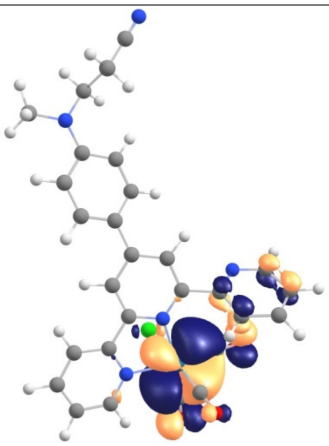

H–2	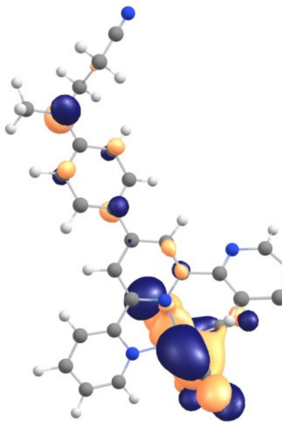	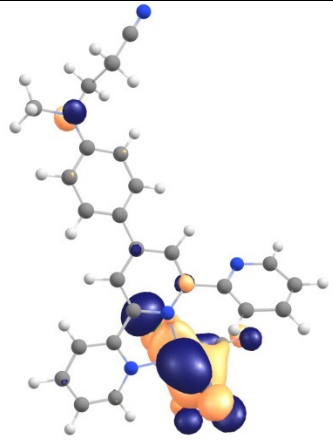
H–1	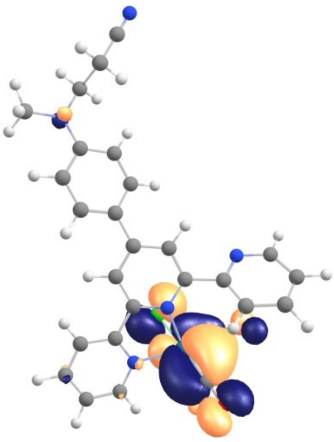	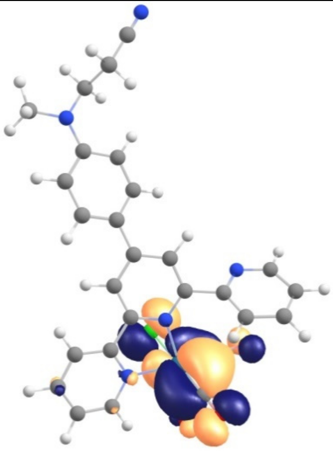
HOMO	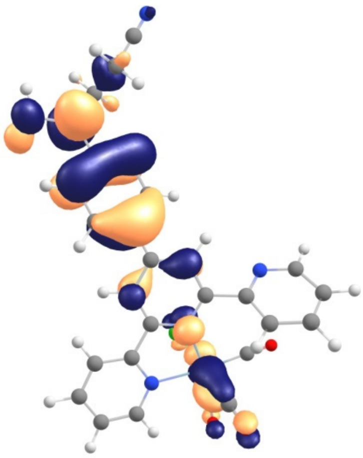	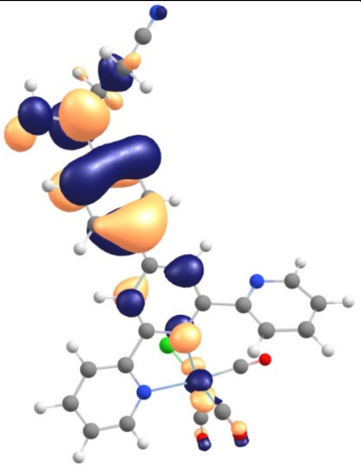
LUMO	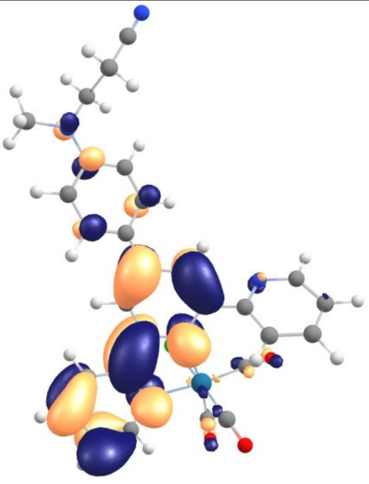	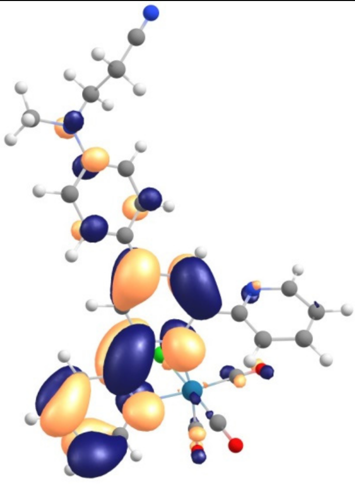

L+1	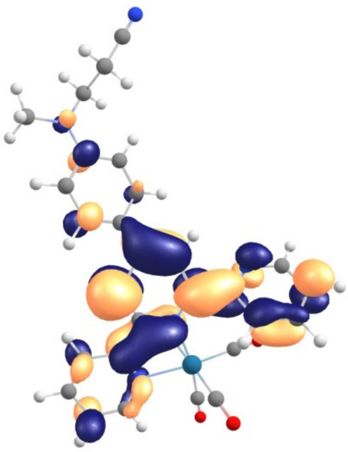	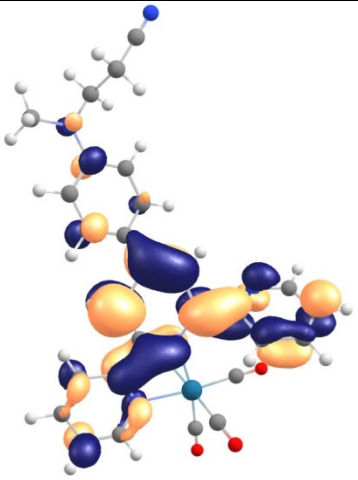

Photoluminescence studies

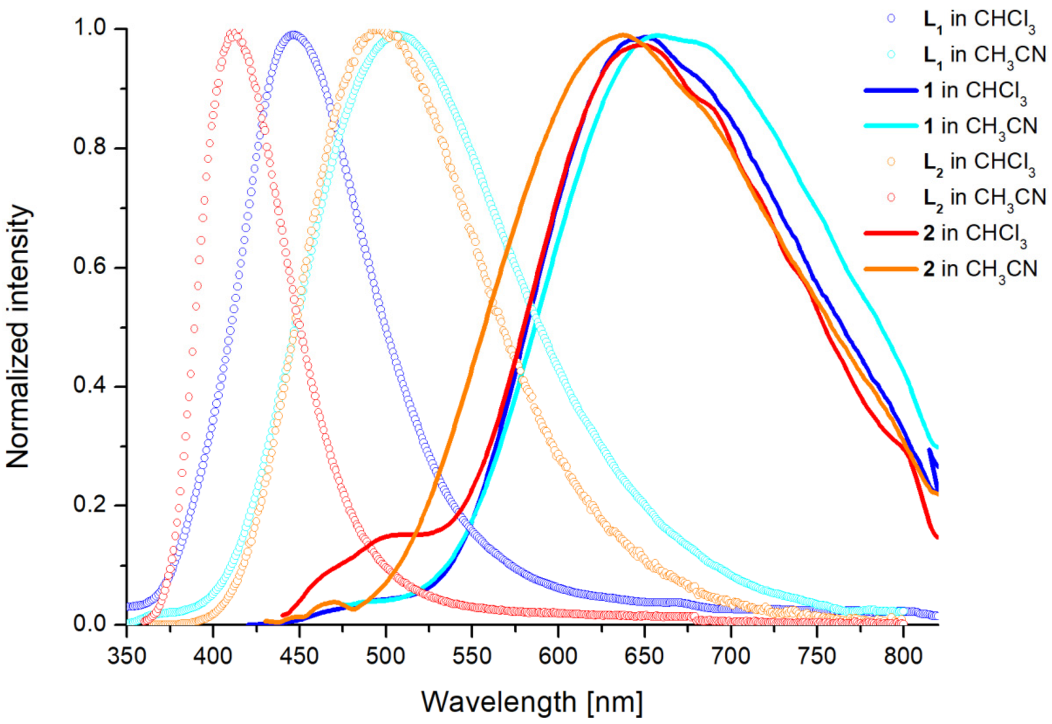

Figure S28. Normalized emission spectra of **1** and **2** in comparison to those for free ligands

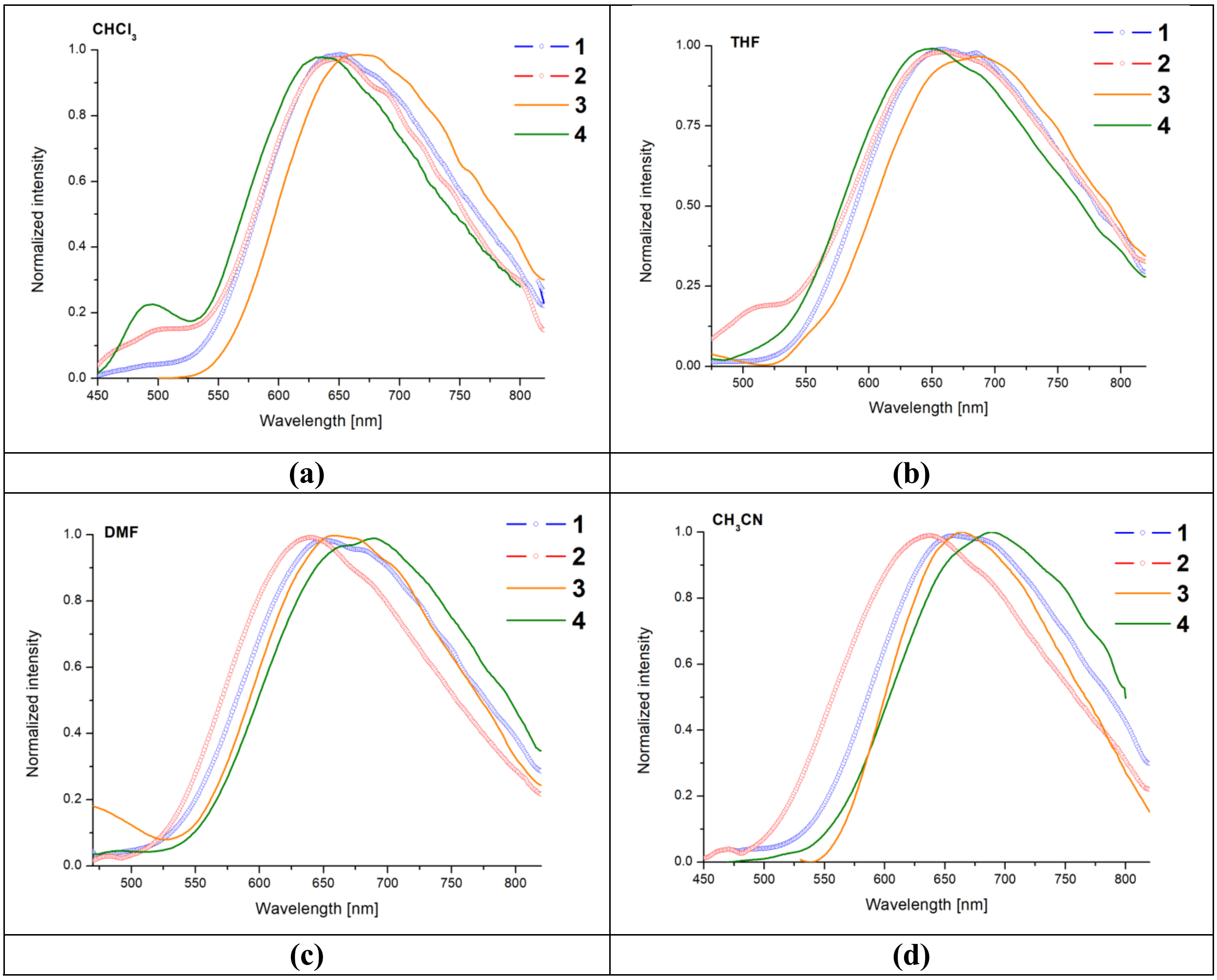

Figure S29. Normalized luminescence spectra of **1–4** in deaerated chloroform (a), tetrahydrofurane (b), N,N–dimethylformamide (c) and acetonitrile (d).

Table S10. Relevant photophysical parameters of **1** and **2** in comparison to those for $[ReCl(CO)_3(C_6H_5\text{-terpy-}\kappa^2N)]$ (**3**) and $[ReCl(CO)_3(Me_2N\text{-}C_6H_4\text{-terpy-}\kappa^2N)]$ (**4**).

Compound	Medium	λ_{ex} [nm]	λ_{em} [nm]	Stokes shift [cm^{-1}]	τ	τ_{av}	Φ
**1**	$CHCl_3$	405	645	9190	8.10 ns	8.10 ns	0.009
	THF	405	665	9840	4.72 ns (43.81%) 7.12 ns (56.19)	6.07 ns	0.01
	DMF	409	650	9060	9.51 ns (7.08%), 266.39 ns (92.92%)	248.20 ns	0.001
	CH_3CN	405	665	9650	9.00 ns (15.06%), 170.42 ns (84.94%)	146.11 ns	<0.001
	77K (EtOH-MeOH)	420	560	5950	80.75 µs (32.16%), 223.81 µs (67.84%)	177.80 µs	–
	solid	475	613	4740	346.71 ns (47.39%), 738.59 ns (52.61%)	552.88 ns	0.071
**2**	$CHCl_3$	430	643	7700	7.36 ns (98.0%), 30.49 ns (1.96 %)	7.81 ns	0.026
	THF	445	665	7430	5.40 ns (97.52%), 29.01 ns (2.48)	5.99 ns	0.027

	DMF	420	640	8180	100.69 ns (19.67%), 696.39 ns (80.33%)	579.22 ns	0.003
	CH_3CN	415	640	8470	11.27 (18.65%), 100.42 (76.38%)	78.80 ns	0.003
	77K (EtOH-MeOH)	410	564	6660	75.46 μs (28.46%), 211.490 μs (71.54%)	172.76 μs	–
	solid	450	560,589,649	6810	326.31 ns (39.61%), 1157.5 ns (60.39%)	828.27 ns	0.003
[ReCl(CO)$_3$(Ph-terpy)] (**3**)	$CHCl_3$	400	667	10010	3.27 ns (71.01%), 28.45ns (28.99%)	10.57 ns	0.004*
	THF	400	676	10210	1.98 ns (61.56%), 19.52 ns (38.44%)	8.72 ns	0.002
	DMF	385	665	10940	1.77 ns (56.75%), 16.61 ns (43.25%)	7.18 ns	0.002
	CH_3CN	380	666	11300	2.18 ns (71.42%), 16.01 ns (28.58%)	6.13 ns	0.002
	77K (EtOH-MeOH)	384	543	7630	2.28 μs (22.41%), 5.88 μs (77.59%)	5.07 μs	–
	solid	442	592	5730	30.04 ns (65.78%), 94.89 ns (34.22%)	52.23 ns	0.012
[ReCl(CO)$_3$((CH$_3$)$_2$-N-C$_6$H$_4$-terpy)] (**4**)	$CHCl_3$	425	637	7830	17.4 ns (94.4%), 63.9 ns (5.6%)	20.00 ns	0.013
	THF	415	650	8710	23.1 ns (42.54%), 64.08 ns(57.46%)	46.64 ns	40.045
	DMF	425	680	7520	27.44 ns (1.28%), 1696.08 ns (98.72%)	1.67 μs	<0.001
	CH_3CN	456	687	7370	2.65ns (10.03%), 197.12 ns (89.97%)	177.60 ns	0.020
	77K (EtOH-MeOH)	444	580	5280	263.0 μs (77.9%), 103.0 μs (22.1%)	227.64 μs	–
	solid	494	636	4520	0.3 μs (61.8%), 3.1 μs (38.2%)	1.37 μs	0.007

* Klemens, T.; Świtlicka, A.; Szlapa-Kula, A.; Krompiec, S.; Lodowski, P.; Chrobok, A.; Godlewska, M.; Kotowicz, S.; Siwy, M.; Bednarczyk, K.; et al. Experimental and Computational Exploration of Photophysical and Electroluminescent Properties of Modified 2,2′:6′,2″-Terpyridine, 2,6-Di(Thiazol-2-Yl)Pyridine and 2,6-Di(Pyrazin-2-Yl)Pyridine Ligands and Their Re(I) Complexes. *Appl. Organomet. Chem.* **2018**, *32*, e4611, doi:10.1002/aoc.4611.

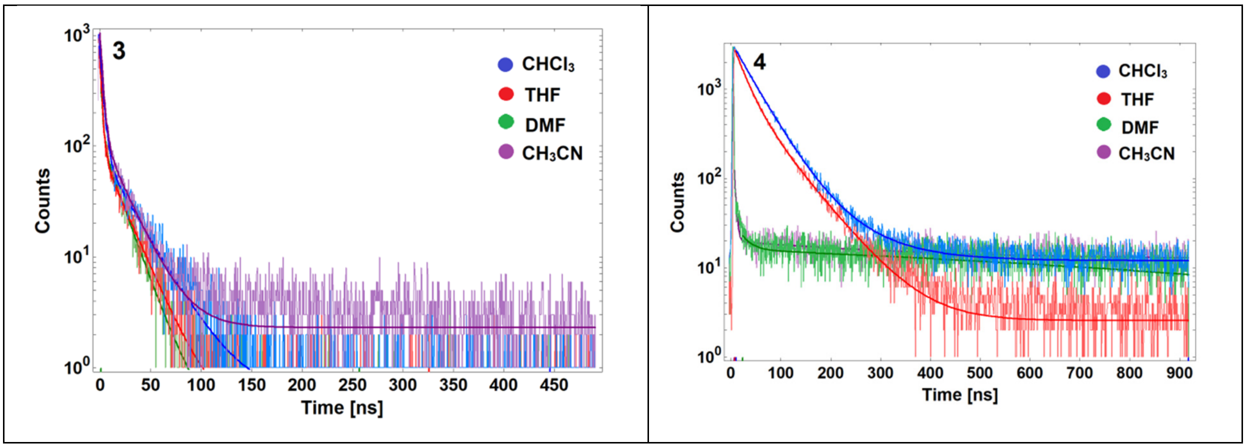

Figure S30. TCSPC decay curves for **3** and **4** in different solvents.

$CHCl_3$	THF
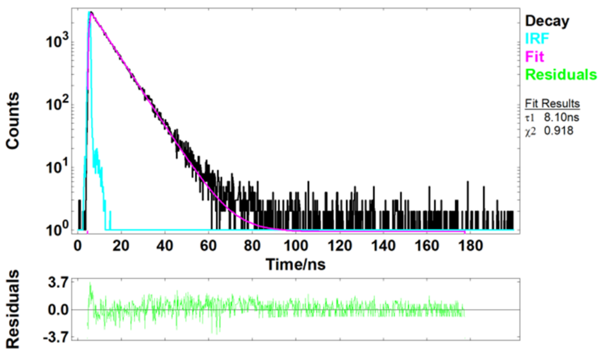	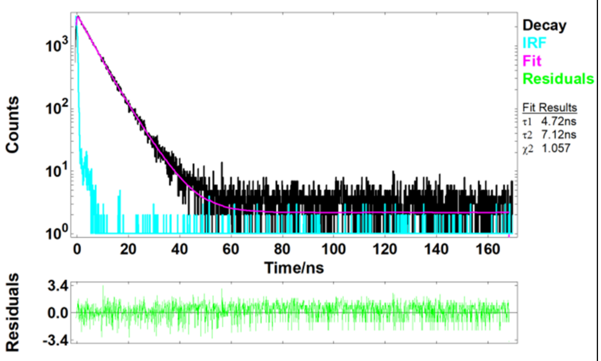
Fit results: τ_1 = 8.10 ns ± 0.02 ns	Fit results: τ_1 = 4.72 ns ± 0.33ns (43.81 %)<br>τ_2 = 7.12 ns ± 0.31 ns (56.19 %)
**DMF**	**CH_3CN**
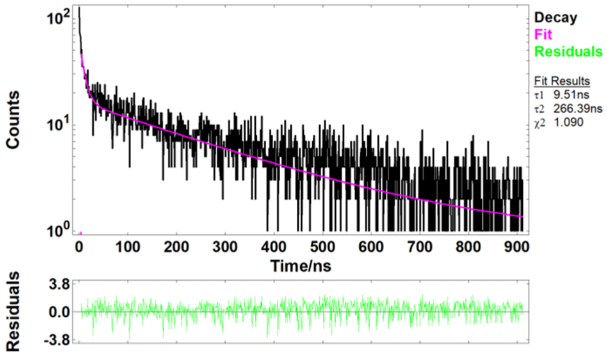	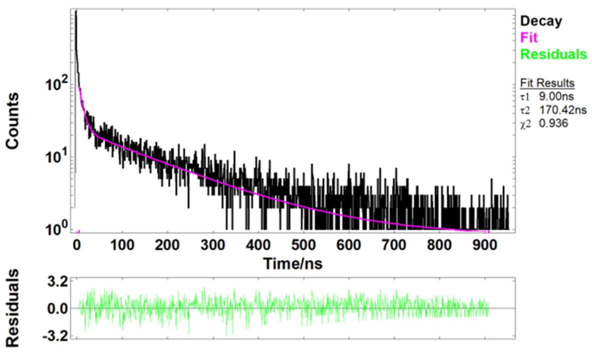
Fit results: τ_1 = 9.51 ns ± 1.53 ns (7.08 %)<br>τ_2 = 266.39 ns ± 17.08 ns (92.92 %)	Fit results: τ_1 = 9.00 ns ± 0.86 ns (15.06 %)<br>τ_2 = 170.42 ns ± 6.80 ns (84.94 %)
**77K**	**Solid state**
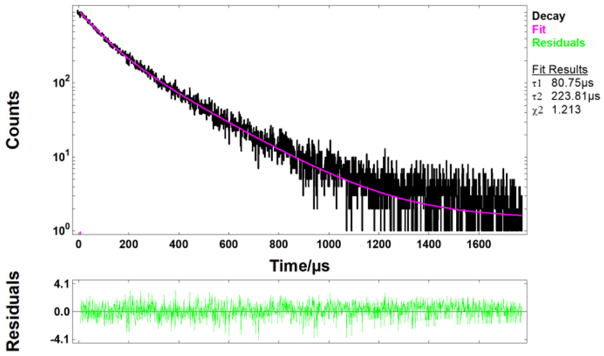	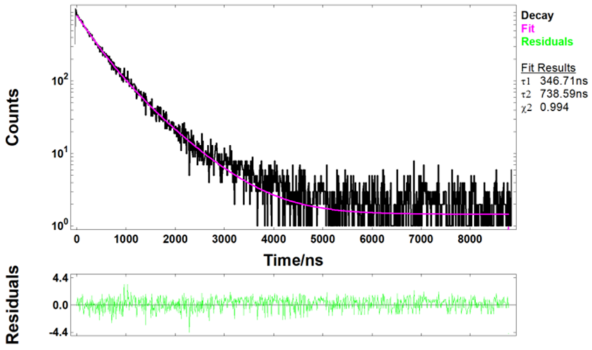
Fit results: τ_1 = 80.75 μs ± 2.57 μs (32.16 %)<br>τ_2 = 223.81 μs ± 3.27 μs (67.84 %)	Fit results: τ_1 = 346.71 ns ± 19.50 ns (47.39 %)<br>τ_2 = 738.59 ns ± 33.08 ns (52.61 %)

Figure S31. Decay curves of **1** in deaerated $CHCl_3$, THF, DMF, CH_3CN at room temperature, in ethanol-methanol rigid-glass matrix (77 K) and solid state.

$CHCl_3$

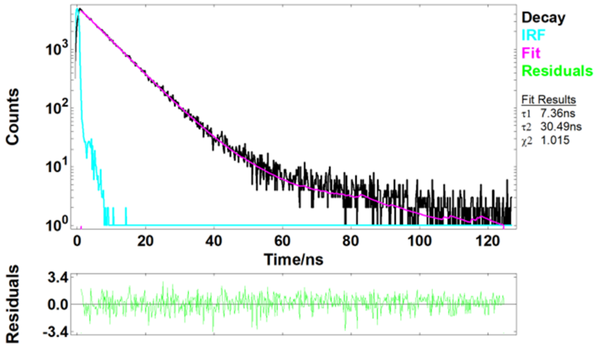

Fit results: τ_1 = 7.36 ns ± 0.04 ns (98.04 %)
τ_2 = 30.49 ns ± 5.95 ns (1.96%)

THF

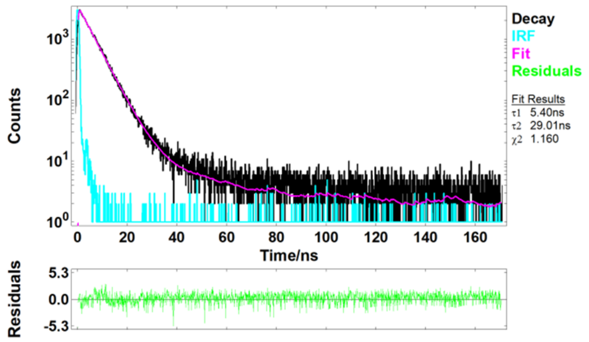

Fit results: τ_1 = 5.40 ns ± 0.02 ns (97.52 %)
τ_2 = 29.01 ns ± 2.85 ns (2.48 %)

DMF

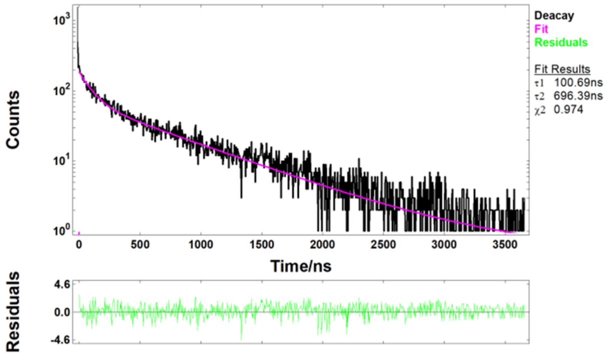

Fit results: τ_1 = 100.69 ns ± 7.30 ns (19.67 %)
τ_2 = 696.39 ns ± 16.07 ns (80.33 %)

CH_3CN

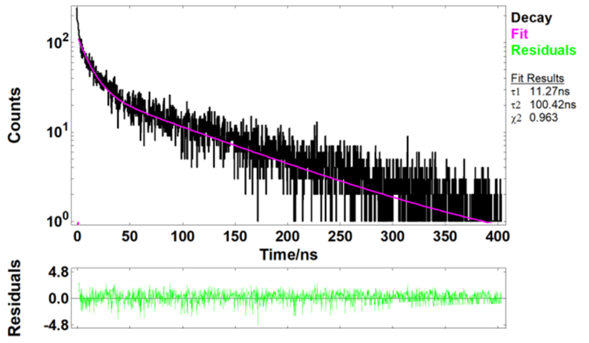

Fit results: τ_1 = 11.27 ns ± 0.55 ns (18.65 %)
τ_2 = 100.42 ns ± 2.87 ns (76.38 %)

77K

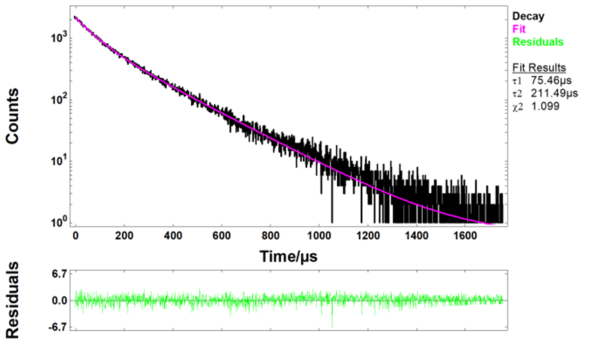

Fit results: τ_1 = 75.46 μs ± 1.61 μs (28.46 %)
τ_2 = 211.49 μs ± 1.55 μs (71.54 %)

Solid state

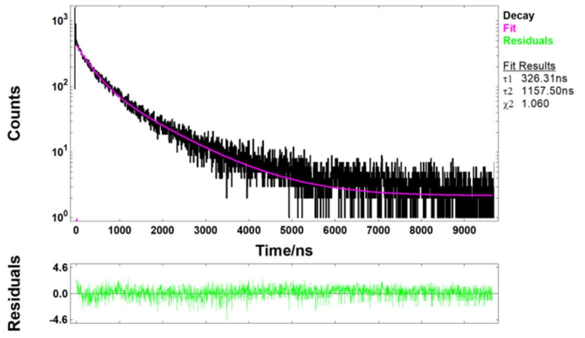

Fit results: τ_1 = 326.31 ns ± 10.35 ns (39.61 %)
τ_2 = 1157.50 ns ± 25.22 ns (60.39 %)

Figure S32. Decay curves of **2** in deaerated $CHCl_3$, THF, DMF, CH_3CN at room temperature, in ethanol-methanol rigid-glass matrix (77 K) and solid state.

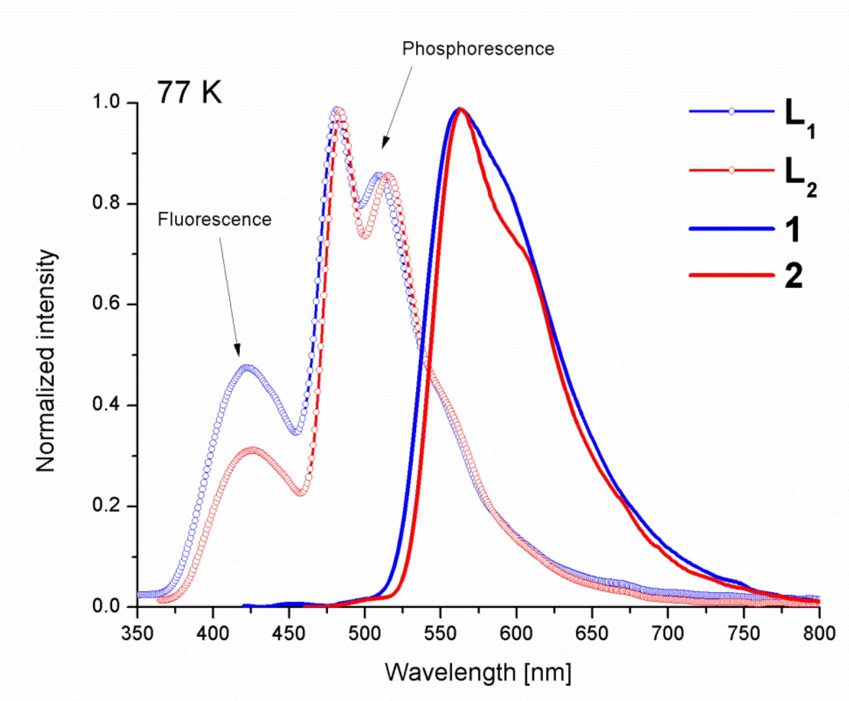

Figure S33. Normalized emission spectra of the free ligands and their Re(I) complexes in ethanol-methanol rigid-glass matrix (77 K). The emission spectra of the free ligands were recorded with the addition of 10% ethyl iodide. The highest energy band in the emission spectra of the free ligands corresponds to the unquenched fluorescence.

Table S11. Calculated phosphorescence emission energies (DFT/PBE1PBE/def2-TZVPD/def2-TZVP) of **1** and **2**, compared to the experimental values recorded in acetonitrile solution.

Compound	TD-DFT				λ_{exp} [eV/nm]
	Major contribution	E [eV]	λ_{cal} [nm]	Character	
**1** $CHCl_3$	H→L (84%)	1.88	659	ILCT/IL and MLCT	1.93/642
**1** CH_3CN	H→L (86%)	1.89	657	ILCT/IL and MLCT	1.86/665
**2** $CHCl_3$	H→L (94%)	1.73	718	ILCT/IL and MLCT	1.93/643
**2** CH_3CN	H→L (85%)	1.96	631	ILCT/IL and MLCT	1.94/640

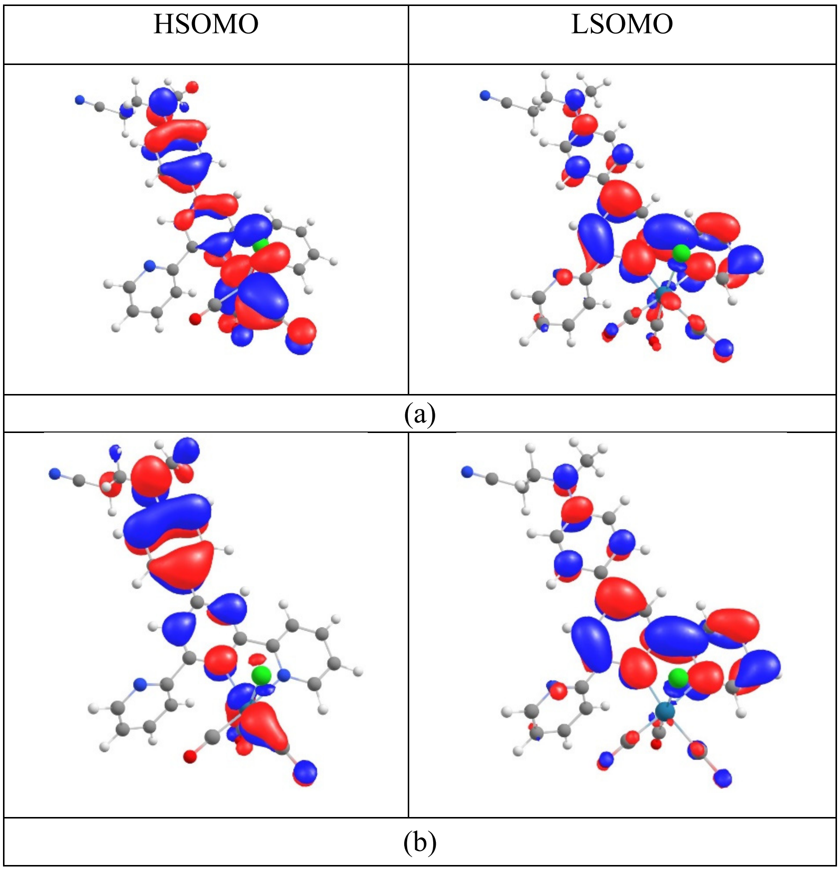

Figure S34. Representative isodensity surface plots of the LSOMO and HSOMO for complex **2** (TD-DFT at T_1 state geometry, red and blue colors show regions of excess α spin density and excess β spin density values, respectively), as well as the spin density surface plots (DFT, T_1 state geometry; green and blue colors show regions of excess α spin density and excess β spin density values, respectively) simulated with the PCM model in chloroform (a) and acetonitrile (b).

Femtosecond transient absorption spectroscopy

1 in CH_3CN

Fluence test was performed at 20 ps time delay, in the range 40 to 220 µW. Compound **1** showed a linear regime within range 40–100 µW. The fsTA measurement of 1 was carried out at 70 µW, approaching the highest value of power pump within the linear range.

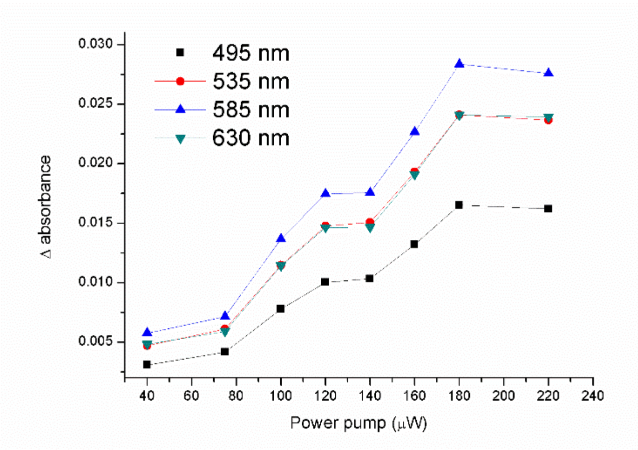

1 in $CHCl_3$

Fluence test was performed at 20 ps time delay, in the range 40 to 250 µW. Compound **1** showed a linear regime within range 40-90 µW. The fsTA measurement of **1** was performed at a value of 70 µW, which is in the linear range.

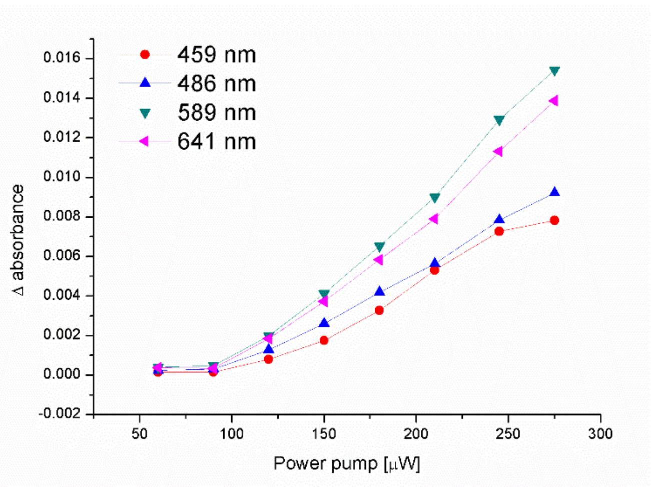

2 in CH_3CN

Fluence test was performed at 20 ps time delay, in the range 70 to 220 μW. Compound **2** showed a linear regime within range 70-160 μW. The fsTA measurement of **2** was performed at a value of 120 μW, which is in the linear range

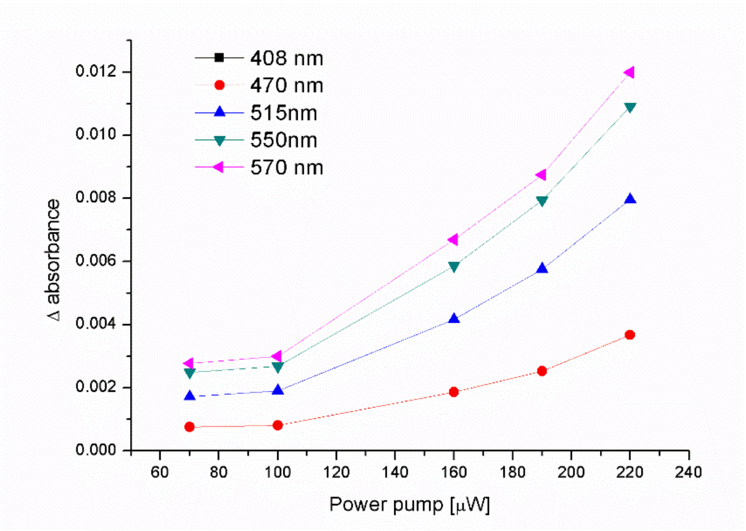

2 in $CHCl_3$

Fluence test was performed at 20 ps time delay, in the range 70 to 220 μW. Compound **2** showed a linear regime within range 70-130 μW. The fsTA measurement of **2** was performed at a value of 130 μW, which is in the linear range.
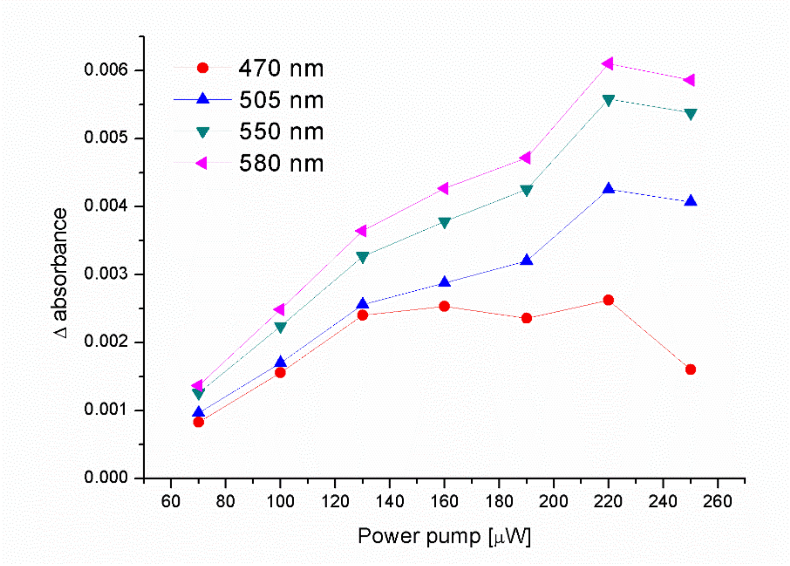

Figure S35. The results of fluence dependence of **1** and **2**.

1 in CH_3CN	2 in CH_3CN

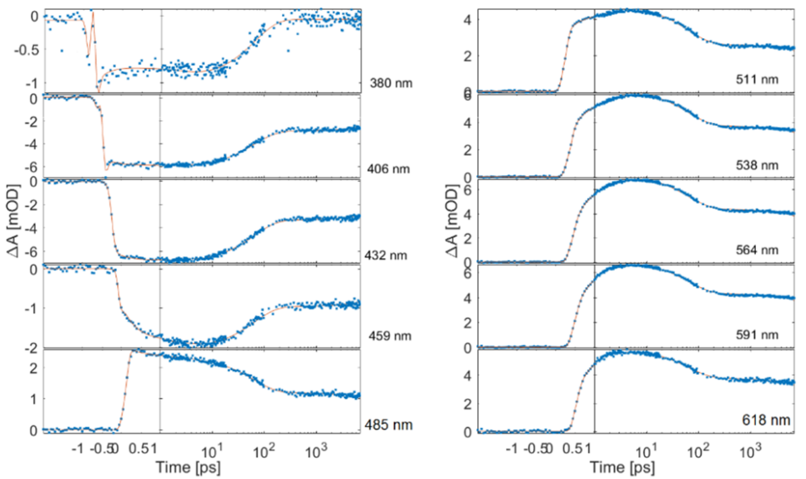

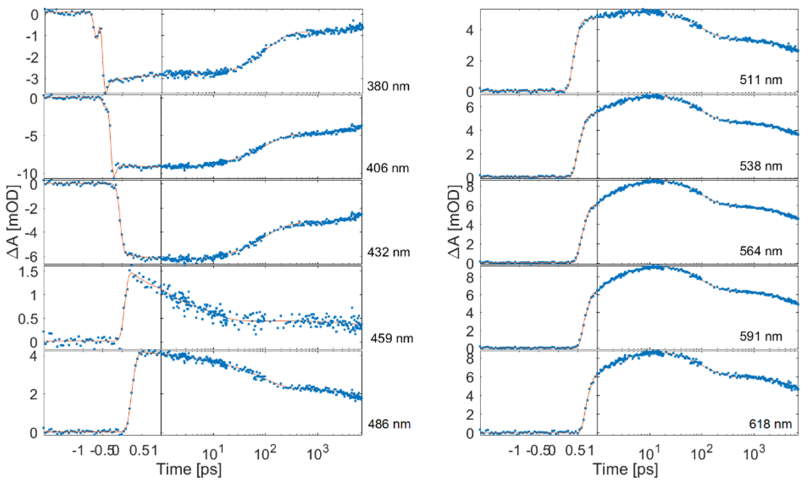

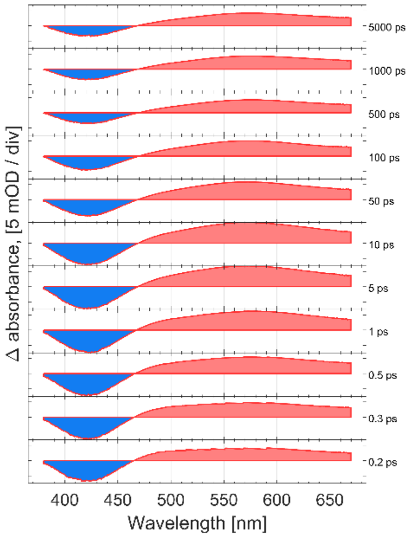

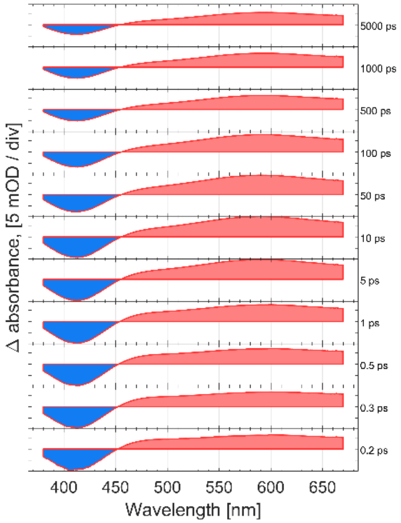

1 in $CHCl_3$	2 in $CHCl_3$

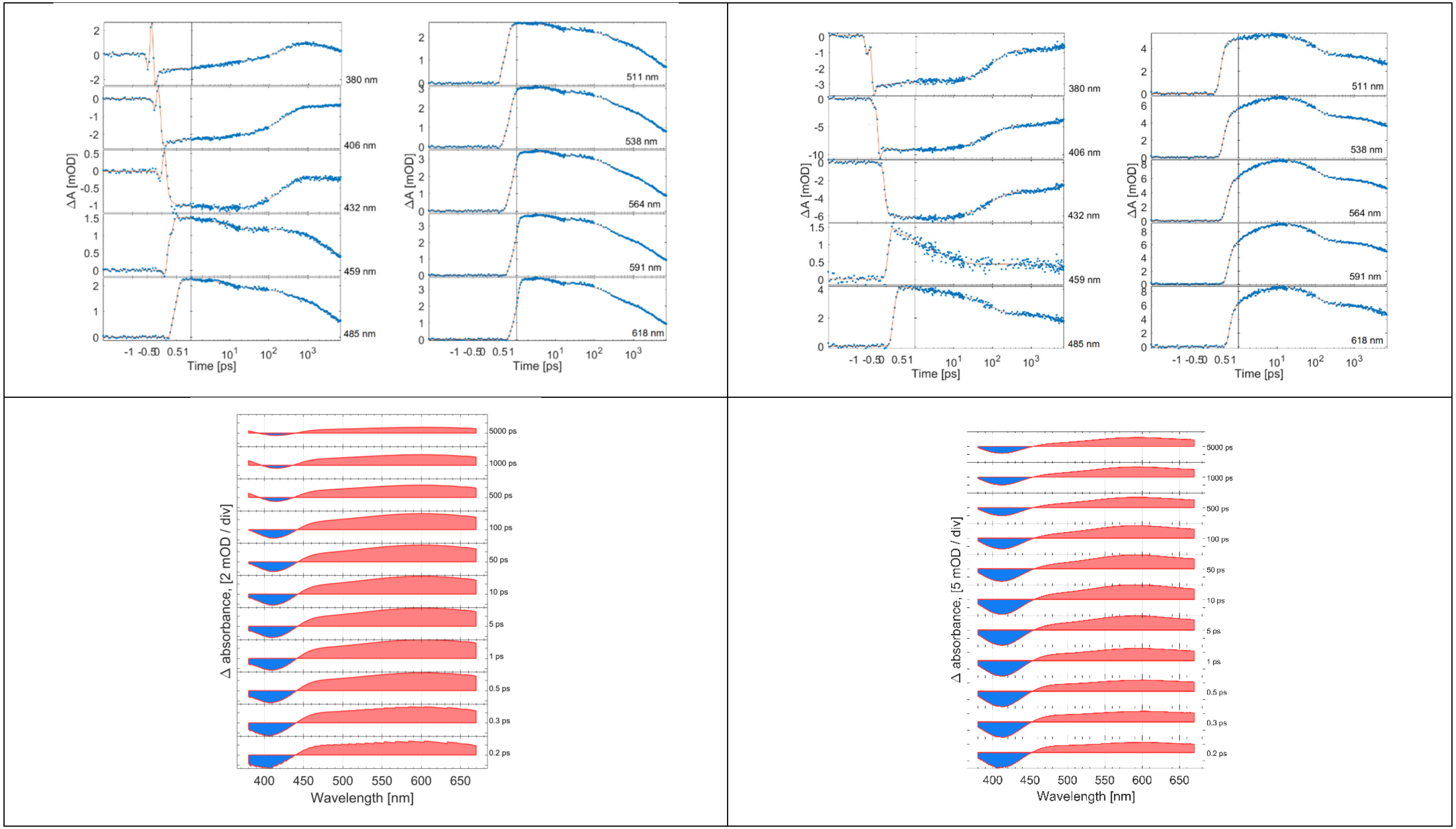

Figure 36. TA spectra at selected time delays and time traces at several wavelength for **1** and **2** in chloroform and acetonitrile.

4 in $CHCl_3$ | **4 in CH_3CN**

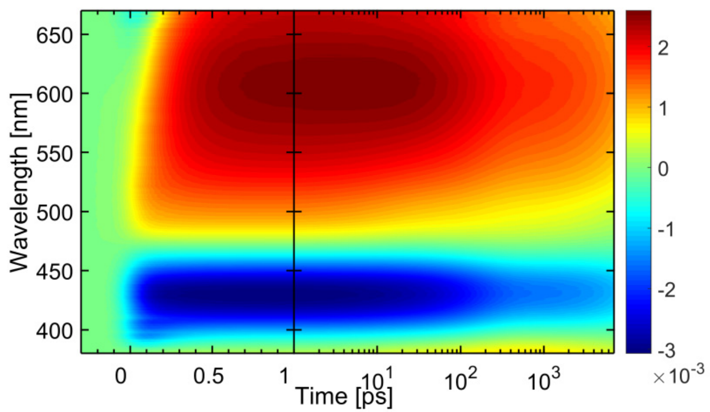

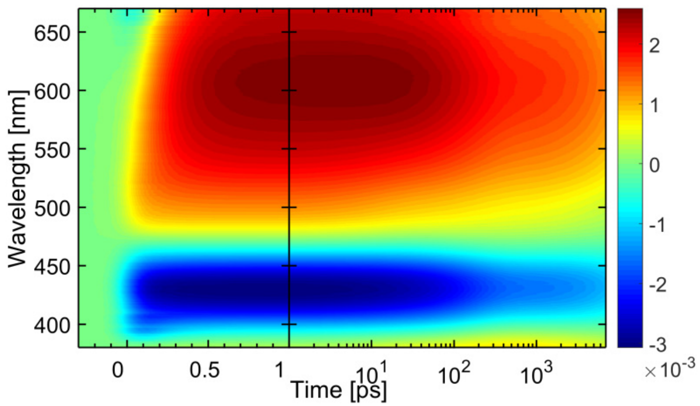

(a)

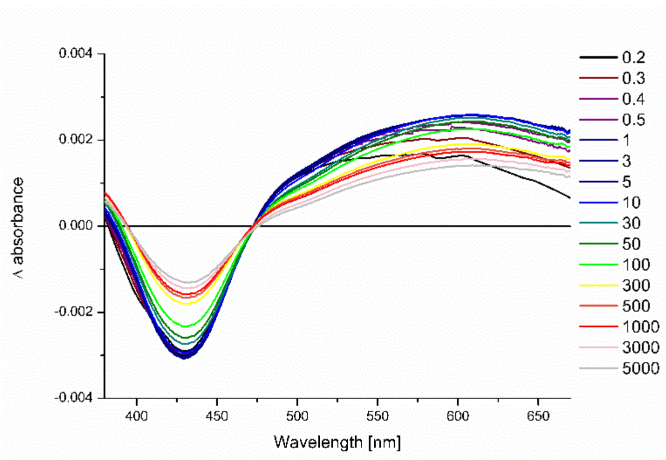

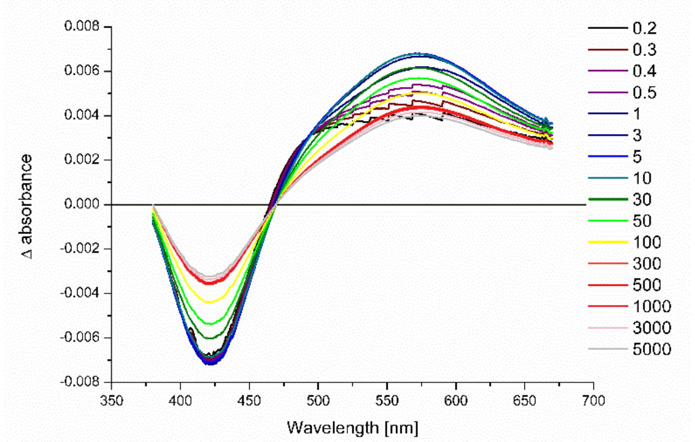

(b)

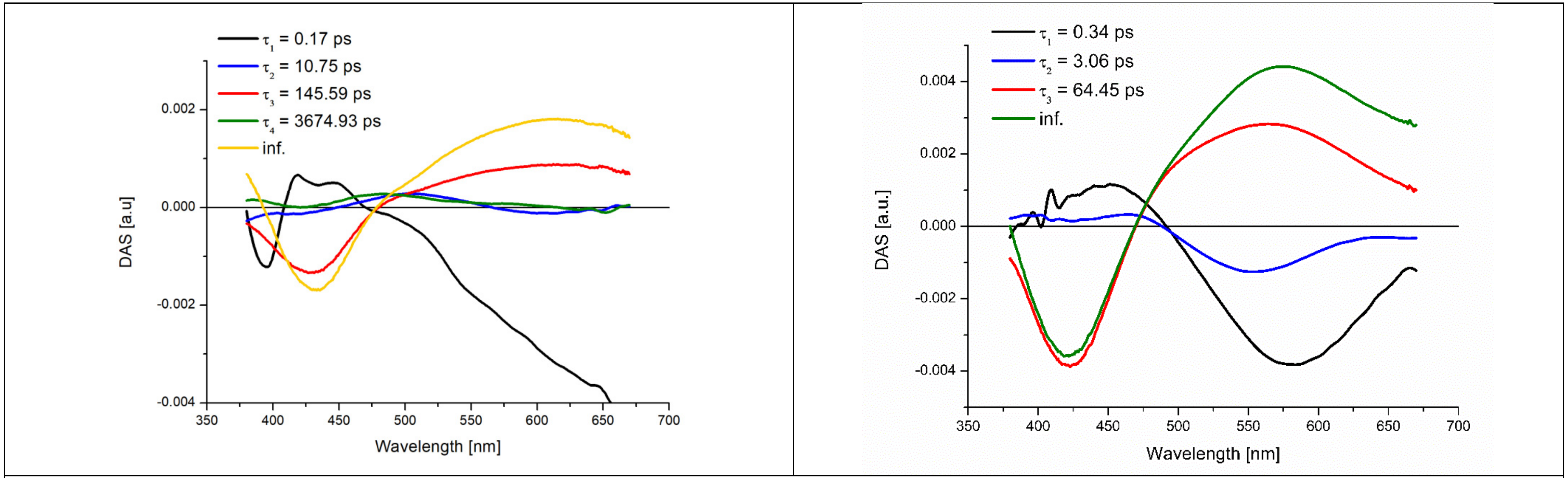
τ1 = 0.17 ps
τ2 = 10.75 ps
τ3 = 145.59 ps
τ4 = 3674.93 ps
inf.
DAS [a.u]
Wavelength [nm]
τ1 = 0.34 ps
τ2 = 3.06 ps
τ3 = 64.45 ps
inf.
DAS [a.u.]
Wavelength [nm]

(c)

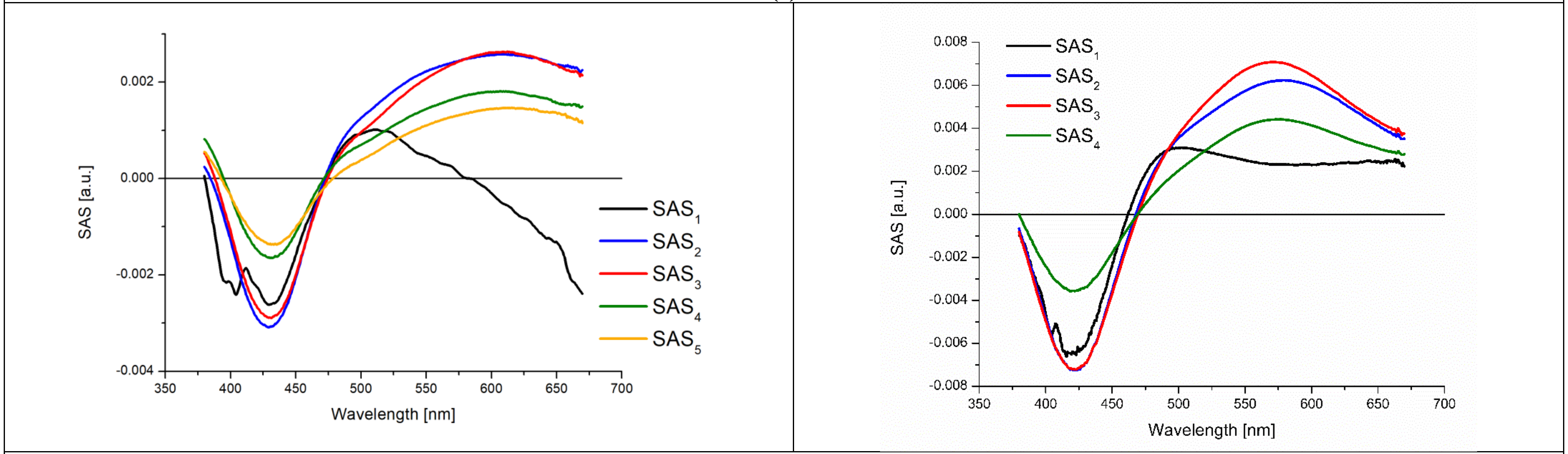
SAS1
SAS2
SAS3
SAS4
SAS5
SAS [a.u.]
Wavelength [nm]
SAS1
SAS2
SAS3
SAS4
SAS [a.u.]
Wavelength [nm]

(d)

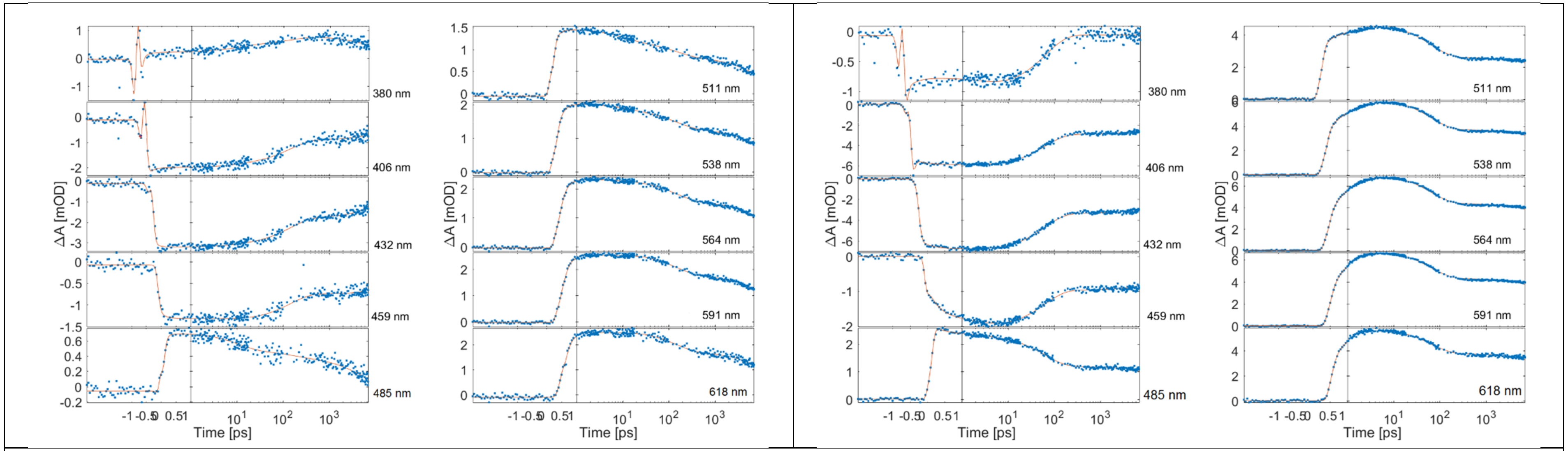

ΔA [mOD]
Time [ps]
380 nm
406 nm
432 nm
459 nm
485 nm
511 nm
538 nm
564 nm
591 nm
618 nm

(e)

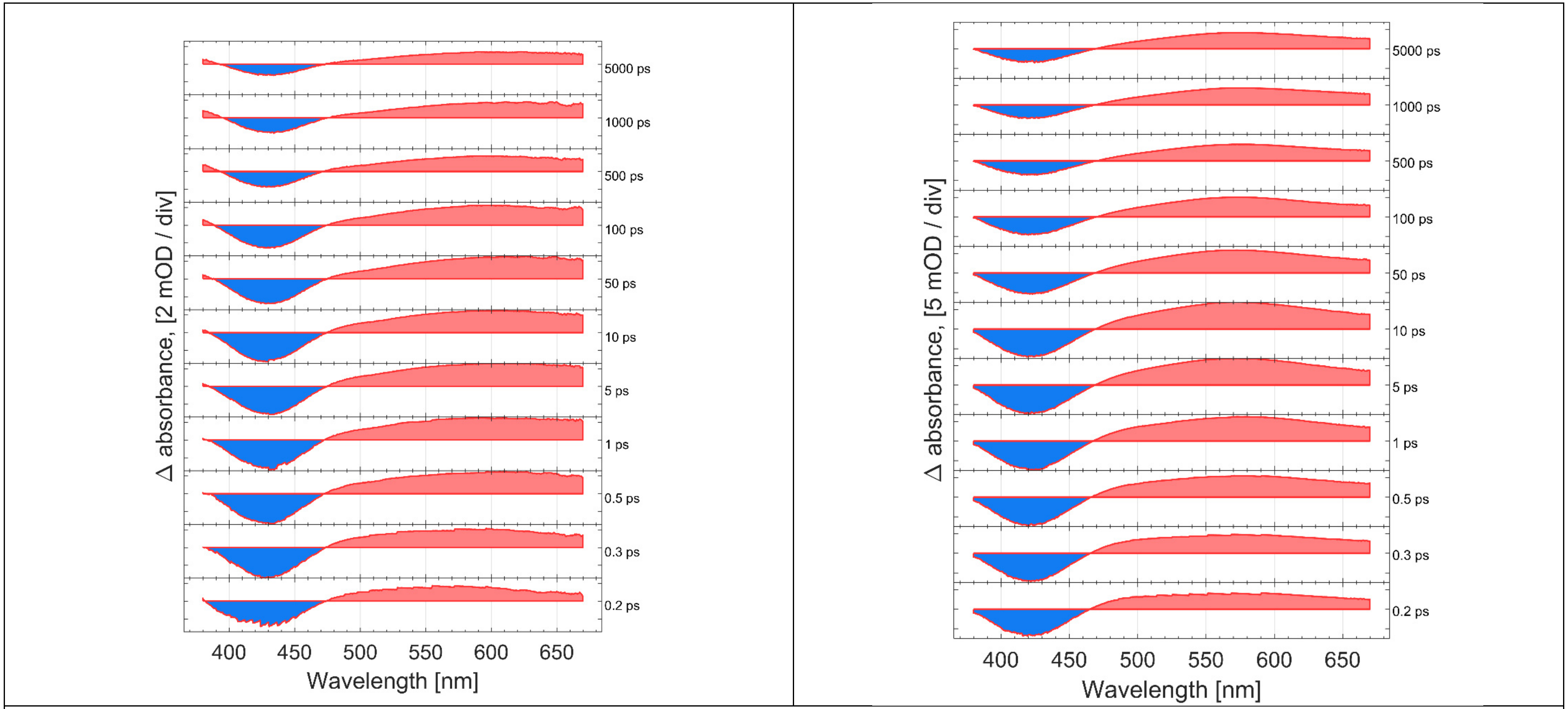

(f)

Figure 37. The fsTA 2D maps (a) and TA spectra at selected time delays (b and f)) decay associated spectra (DAS_i) (c), species associated spectra (SAS_i) (d) and time traces at several wavelength (e) for **4** in chloroform and acetonitrile.

3 in $CHCl_3$	3 in CH_3CN
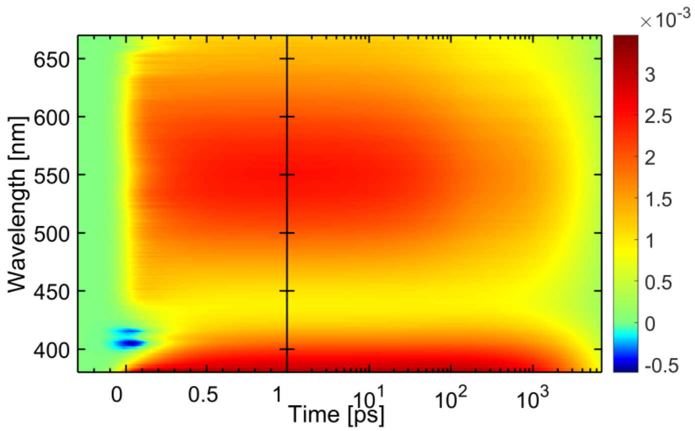	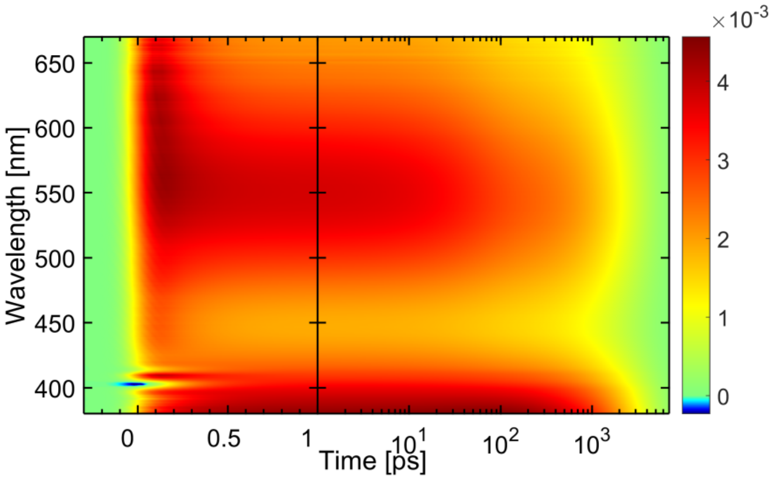
(a)	
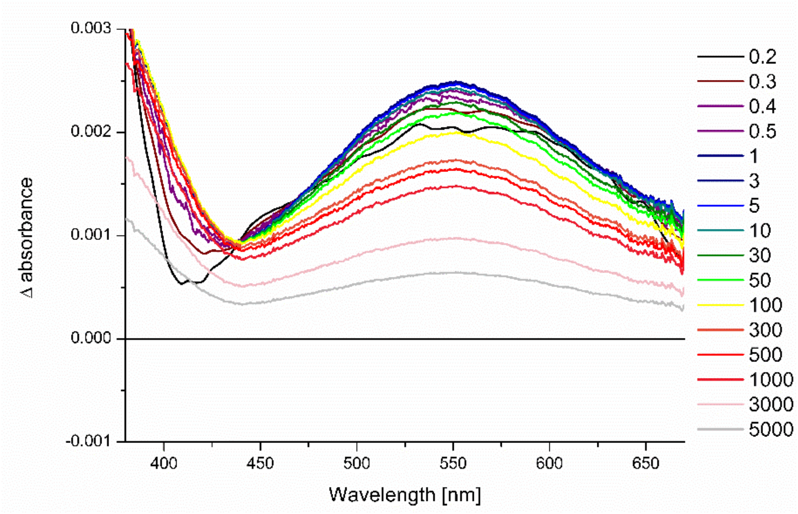	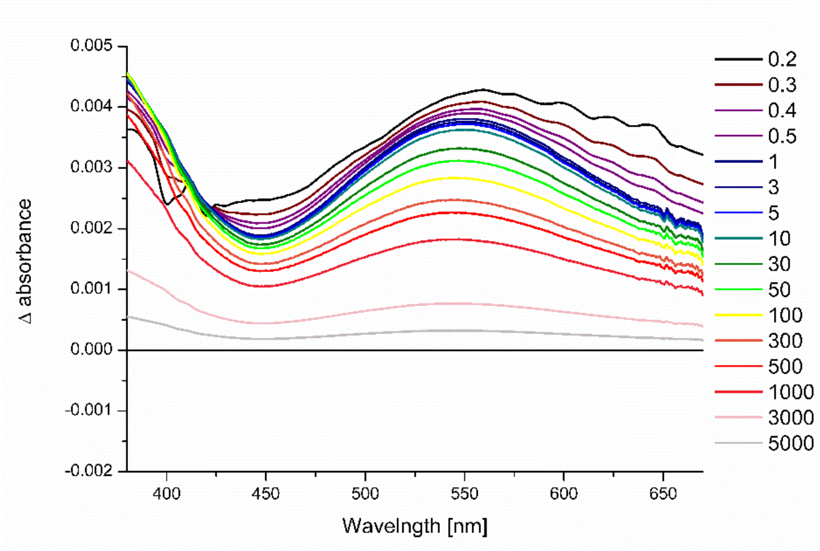
(b)	

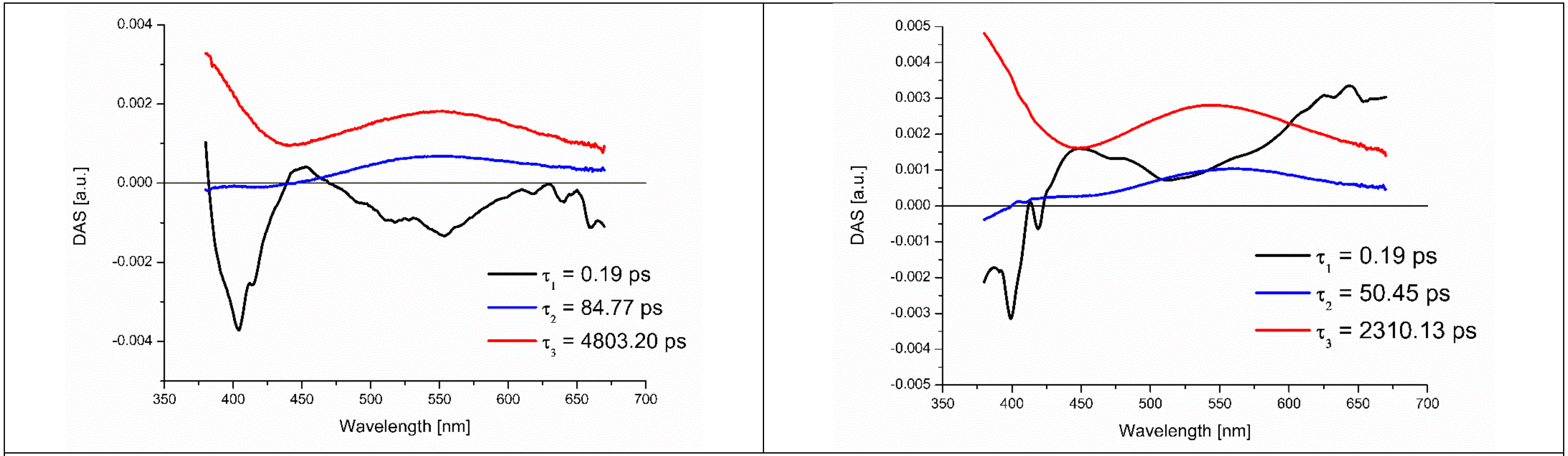
DAS [a.u.]
Wavelength [nm]
τ1 = 0.19 ps
τ2 = 84.77 ps
τ3 = 4803.20 ps
τ1 = 0.19 ps
τ2 = 50.45 ps
τ3 = 2310.13 ps

(c)

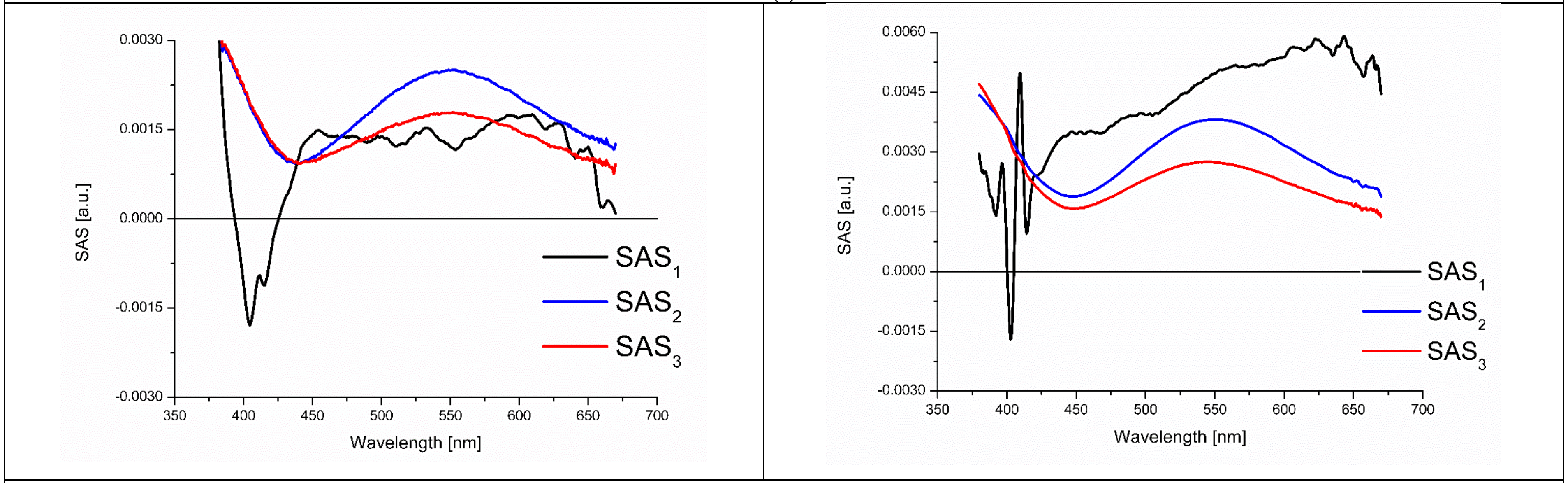
SAS [a.u.]
Wavelength [nm]
SAS1
SAS2
SAS3

(d)

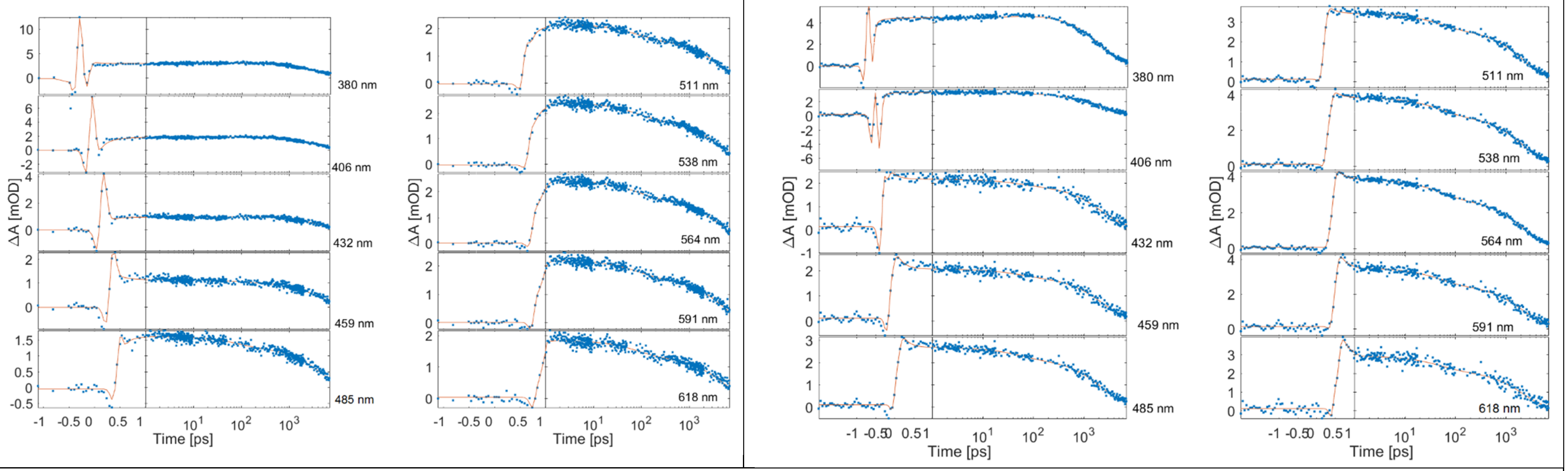
380 nm
406 nm
432 nm
459 nm
485 nm
511 nm
538 nm
564 nm
591 nm
618 nm
ΔA [mOD]
Time [ps]

(e)

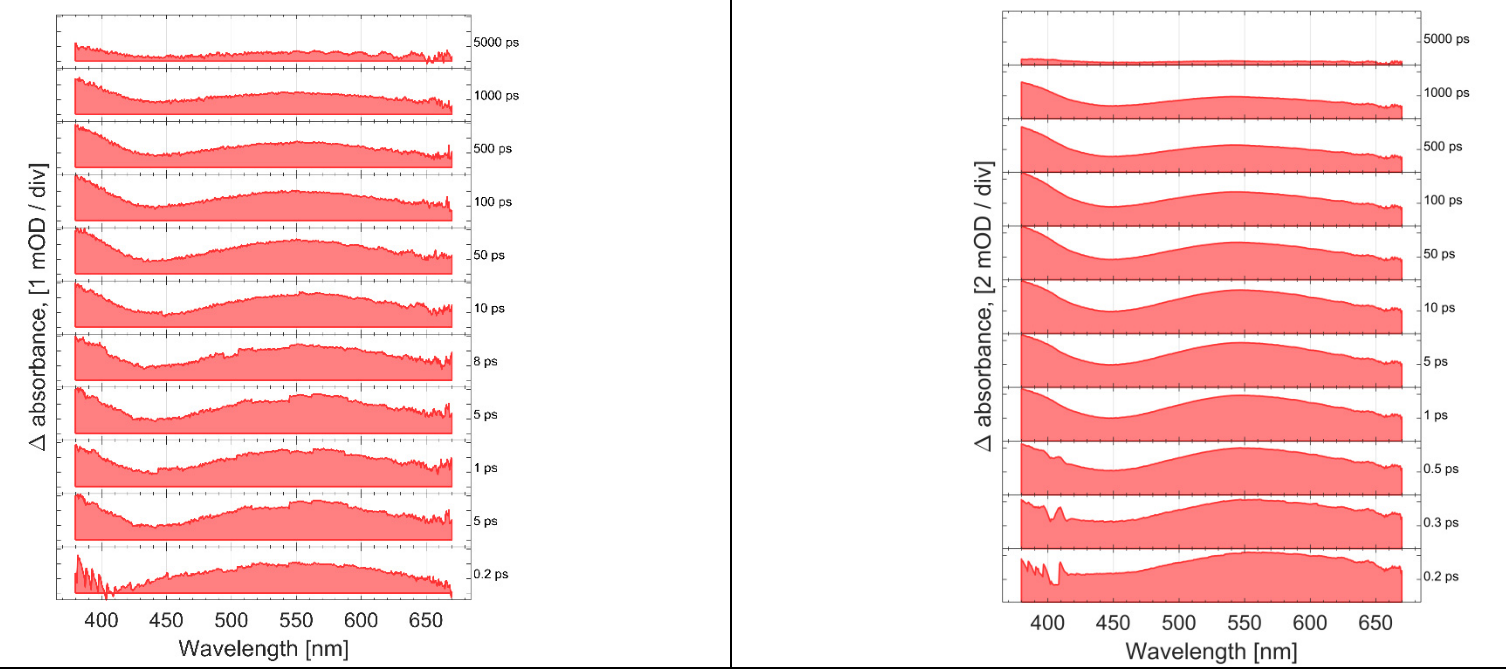

(f)

Figure 38. The fsTA 2D maps (a) and TA spectra at selected time delays (b and f)) decay associated spectra (DAS_i) (c), species associated spectra (SAS_i) (d) and time traces at several wavelength (e) for **3** in chloroform and acetonitrile.
